# Directed C(sp^3^)–H arylation of tryptophan: transformation of the directing group into an activated amide†
†Electronic supplementary information (ESI) available. CCDC 1903900 and 1903901. For ESI and crystallographic data in CIF or other electronic format see DOI: 10.1039/c9sc03440d


**DOI:** 10.1039/c9sc03440d

**Published:** 2019-08-08

**Authors:** Lennart Nicke, Philip Horx, Klaus Harms, Armin Geyer

**Affiliations:** a Philipps-Universität Marburg , Fachbereich Chemie , Hans Meerwein Straße , 35032 Marburg , Germany . Email: geyer@staff.uni-marburg.de

## Abstract

The 8-aminoquinoline (8AQ) directed C(sp^3^)–H functionalization was applied in the synthesis of β-arylated tryptophan derivatives. The laborious protecting group reorganization towards α-amino acids compatible for solid phase peptide synthesis (SPPS) was cut short by the transformation of the directing group into an activated amide, which was either used directly in peptide coupling or in the gram scale synthesis of storable Fmoc-protected amino acids for SPPS. In this work, directed C–H activation and nonplanar amide chemistry complement each other for the synthesis of hybrids between phenylalanine and tryptophan with restricted side chain mobility.

## Abstract 2

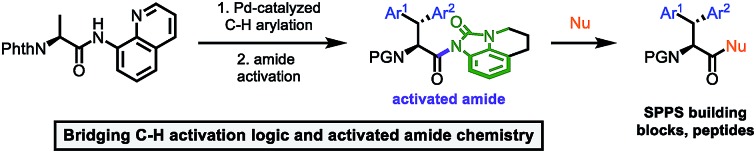
The aminoquinoline-directed C–H activation was used to synthezise unnatural tryptophans for solid phase peptide synthesis for the first time.

## Introduction

**Fig. 1:**
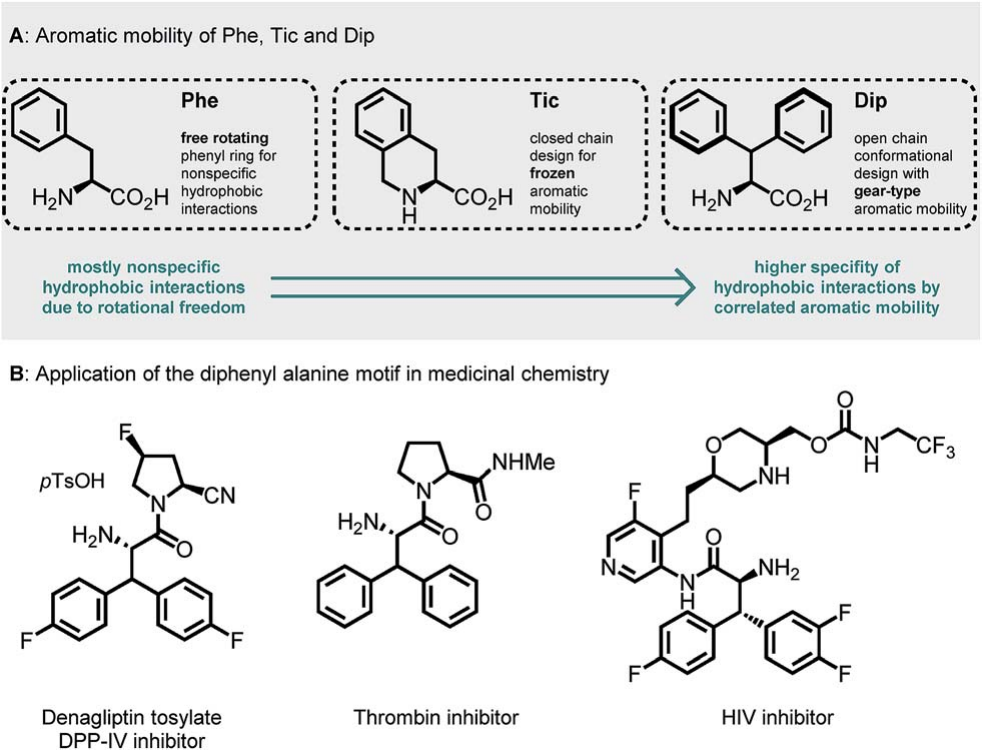
(A) Aromatic mobility and conformational design of Phe, Tic (l-tetrahydroisoquinoline carboxylate) and Dip (l-diphenyl alanine) amino acids.^8^ (B) Therapeutic applications of the β,β-diaryl alanine motif.^7^

Unnatural β-branched α-amino acids are promising tools for the synthesis of peptide ligands with conformational constriction in a topologically designed structure.^1^ The increased hydrophobic surface of β-branched α-amino acids can mediate molecular recognitions processes between bioactive peptides and their target receptors.^2^ Full control of χ-space^3^ is achieved in bicyclic peptidomimetics, which strongly influence the properties of the target peptides and proteins.^4^ The conformational design of open-chain β-branched α-amino acids, however, serves as a valuable platform in peptide ligand design.^2^ Consequently, the investigation of reliable synthetic routes towards β-branched α-amino acids has experienced a renaissance in the past couple of years.^5^ In particular, great attention has been payed to the synthesis of β,β-diaryl α-amino acids due to unique hydrophobic interaction patterns^6^ and promising medicinal applications of therapeutics containing these structural motifs (Fig. 1).^7^

In our investigation of peptide-receptor interactions, focusing on the influence of altered indole presentation on biological activity, we were in requirement of a χ^1^-constricted, conformationally anchored tryptophan building block for standard Fmoc-SPPS. We assumed that β-phenylated tryptophan will be suitable for this purpose. While the synthesis of β,β-diaryl α-amino acids bearing two identical aromatic groups was widely elaborated in earlier studies,^9^ the synthesis of these amino acids containing different aromatic groups is a more challenging endeavor. The construction of β,β-diaryl α-amino acids with different aryl substituents, ensuring full control over two vicinal stereocenters, has stimulated numerous synthetic studies in the field of asymmetric alkylation/conjugate addition,^10^ asymmetric hydrogenation^11^ and directed C–H activation.^12^ Most of these methods focus on the efficient construction of the carbon skeletons and offer remarkable stereocontrol, but neglect the use of versatile removable directing groups to give adequately protected amino acids for peptide synthesis based on the approach of Fmoc/Boc strategy. Thus, to the best of our knowledge, there is no example of a synthetic peptide bearing a nonsymmetrical β,β-diaryl alanine motif.^13^,^14^ Inspired by the seminal work of Corey,12*a* as well as Daugulis,12*b* Yu12c,d and Chen,^15^ we envisioned that diastereomeric β-phenyl tryptophans could be accessed employing sequential C–H activation of alanine. In necessity of a strong, bidentate directing group, we chose 8-aminoquinoline (8AQ). Phth-Ala-8AQ and an N-protected 3-iodoindole could be starting materials in a palladium-catalyzed, directed C–H activation to give access to an enantiomerically enriched tryptophan derivative.15*b* These compounds have been shown to be competent substrates in β-alkynylation^16^ and might also undergo arylation. Given the sensitivity of indoles towards oxidative conditions,12*d* we evaluated a mild method of 8-aminoquinoline cleavage. This effort has proven to be an unnerving challenge^17^ and the practitioner must choose from protocols only suitable for a limited class of substances. In spite of numerous reactions employing aminoquinoline-directed C–H activation chemistry,^18^ examples of its use in natural product or functional molecule synthesis are rare.^19^ The removal of 8-aminoquinoline often demands harsh reaction conditions, which represents a major drawback in synthetic applications.^20^,12b Studies addressing this concern have been published by Maulide, using an ozonolysis approach,^21^ Ohshima, using a Nickel-chelate assisted methanolysis^22^ and Mashima, utilizing epoxide opening for tandem esterification.^23^ A recent report by Chen *et al.* utilized IBX in an oxidative protocol for 8AQ cleavage to give various α-amino acids as amides in high yields, however, Trp formed a *spiro*-fused compound and could not be deprotected accordingly.^24^ Standard procedures of amide activation using Boc_2_O to generate a labile imide, show limited applicability, because they only tolerate substrates with low steric hindrance.^25^ More sterically demanding substrates usually require strain-releasing steps using hazardous TfN_3_ to achieve amide activation and directing group removal.^26^ From our point of view, an applicable method for directing group removal has to fulfil the following premises:

- high functional group tolerance

- preservation of stereochemical integrity

- compatibility with standard SPPS protecting groups

- useful yields in large-scale applications

**Scheme 1:**
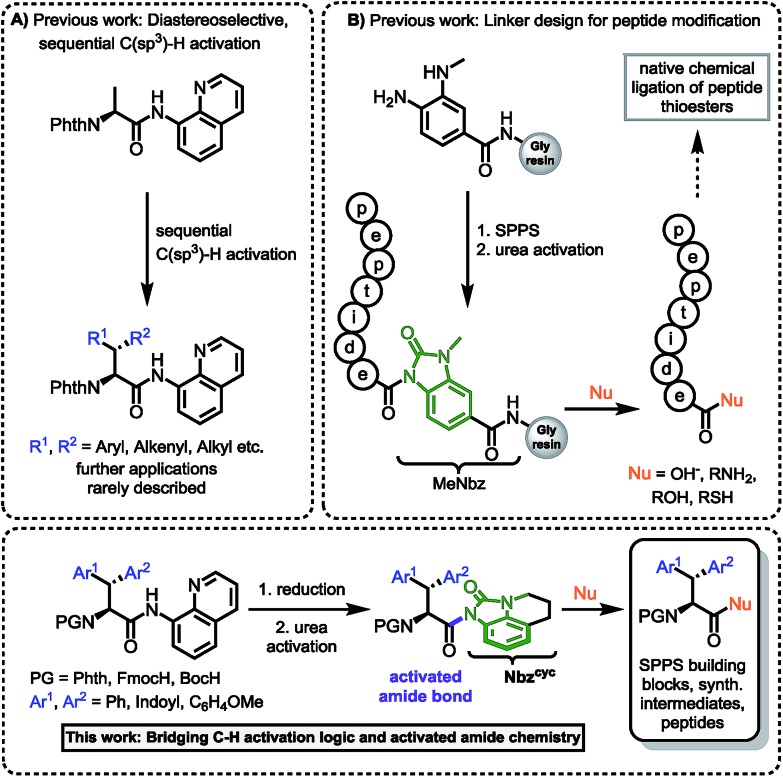
(A) Sequential C(sp^3^)–H activation.12a,b,^13^ (B) Linker design for C-terminal peptide modification.^27^ This work: Rationale of amidic weakening strategy applied for α-amino acids prepared by directed C–H activation.

Hence, we were intrigued by the structural similarity of 8-aminoquinoline amides and the widely used Dawson linker for C-terminal diversification of resin-bound peptides.^27^ A similar amide activation strategy could be feasible for 8-aminoquinoline amides, that opens new possibilities towards C-terminal amino acid modifications (Scheme 1).

## Results and discussion

**Scheme 2:**
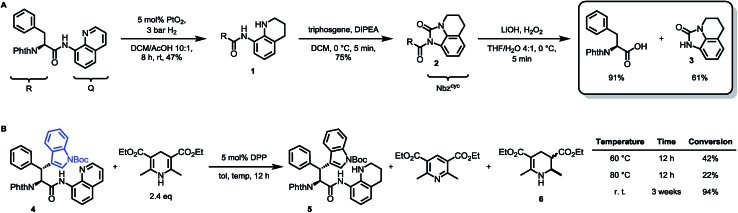
(A) Validation of 8-aminoquinoline cleavage by amidic weakening strategy. (B) Reaction outcome using Rueping's conditions.

To prove this concept, we subjected the common starting material Phth-Phe-8AQ to hydrogenation conditions to generate a 1,2,3,4-tetrahydroquinoline species. 5 mol% PtO_2_-catalyzed hydrogenation in DCM/AcOH gave rise to reduction product **1** in irreproducible yields with concomitant formation of non-identifiable byproducts (Scheme 2).

**Scheme 3:**
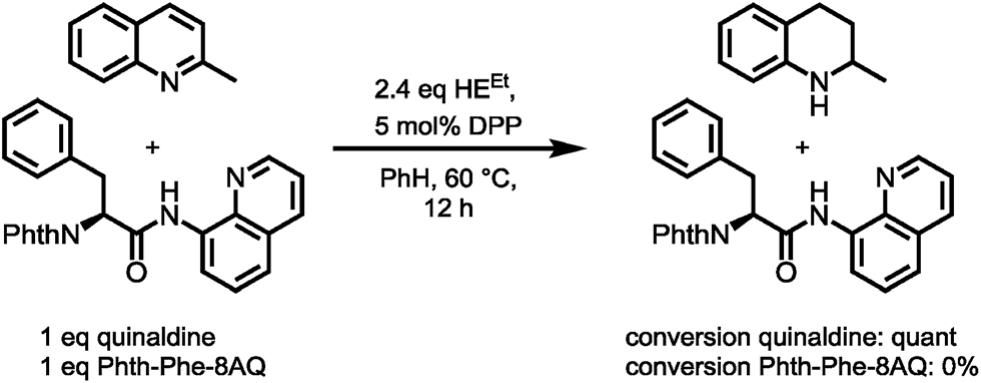
Competition experiment between quinaldine and Phth-Phe-8AQ.

However, enough starting material was generated to perform a triphosgene amide activation, which has previously been used in custom-made directing group removal.^28^ Treatment of tetrahydroquinoline **1** with triphosgene in DCM in the presence of Hünig's base provided the acylurea **2** in 75% yield. In analogy to Dawson's linker (MeNbz: *N*-acyl benzimidazolinone) for C-terminal peptide modification, we suggest Nbz^cyc^ (activated urea) as abbreviation to indicate a similar, cyclic (index: cyc) arrangement. We were pleased to find that exposure of the urea compound **2** to standard LiOH/H_2_O_2_ hydrolysis conditions gave a clean formation of Phth-Phe-OH without opening of the phthalimide protecting group or decarbonylation of the Nbz^cyc^ group. As a bonus, simple acid/base extraction was enough to separate the pure free carboxylic acid from the cleaved urea **3**. With this valid concept in hand, we started to develop an improved, chemoselective and high yielding procedure for quinoline reduction under conditions described by Rueping for the reduction of simpler quinolines using (chiral) phosphoric acids and Hantzsch ester as reducing agents.^29^ This hydrogenation method shows a high functional group tolerance which was displayed by the successful use of peptide catalysts for stereoinduction on substituted 8-aminoquinolines.^30^ Preliminary trials of reducing the 8-aminoquinoline moiety of Phth-Wrf(Boc)-8AQ^31^**4** (synthesis *vide infra*) with 2.4 eq. Hantzsch's ethyl ester (HE^Et^) in the presence of 5 mol% diphenylphosphate (DPP) in toluene at 60 °C for 12 h gave 42% conversion of the starting material to the corresponding transfer hydrogenation product. Encouraged by these results, we supposed that raising the reaction temperature might lead to higher conversion. Contradictory to our hypothesis, executing this reaction at 80 °C gave only 22% conversion of the starting material after full consumption of HE^Et^, whereas room temperature led to 94% conversion of the starting material after three weeks. With these surprising results in hand, we took an in-depth look in the reaction products, suspecting non-productive pathways to be present, resulting in consumption of reducing agent. Indeed, high reaction temperatures favoured the progression of a Hantzsch ester disproportionation reaction, giving rise to a diastereomeric mixture of tetrahydropyridine **6** (compare scheme 2B) and the oxidized pyridine derivative, respectively. This side reaction was the underlying cause of rapid consumption of reducing agent, which was not observed in the reactions investigated by Rueping.^32^ We hypothesized, that the formation of the tetrahydropyridine is facilitated by the strong acid DPP, acting in a C-protonation step,^33^ followed by iminium reduction with another molecule of Hantzsch ester.^34^ This intriguing alternative reaction pathway prompted us to carry out a competition reaction between quinaldine, a commonly used substrate in Hantzsch ester mediated transfer hydrogenation, and Phth-Phe-8AQ (Scheme 3).

Only quinaldine was reduced in this competition experiment, so that 8-aminoquinoline amides were identified as a substrate with significantly lower reactivity. We reasoned that this finding is due to (1) the lack of beneficial effects of the methyl group participating in the stabilization of cationic intermediates and (2) the electron-donating properties of the amide nitrogen atom. We assumed that these electron-donating effects could potentially be lowered using oxygenophilic Lewis acids. Furthermore, the use of weaker Brønsted acids might decrease the Hantzsch ester's tendency to undergo disproportionation. The results of the reaction screening are surveyed in Table 1.

**Table 1:** Reaction screening of 8AQ reduction. The reactions were conducted in a 50 mg scale in THF. 2.4 eq. of HE^tBu^ was used. 1.0 eq. of Lewis acid and Brønsted acid were used unless otherwise stated

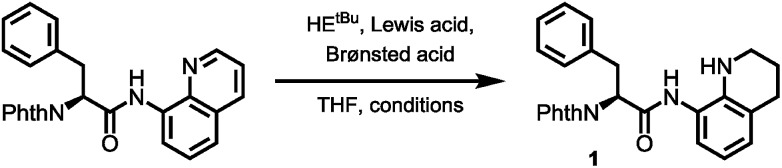					
Entry	Lewis acid	Brønsted acid	Temp.	Time	Conversion (NMR)
1	—	DPP	60 °C	12 h	18%
2	LiBr	DPP	60 °C	12 h	78%
3	LiBr	HCOOH	60 °C	12 h	86%
4	—	HCOOH	60 °C	24 h	*n. r.*
5	LiBr	—	60 °C	24 h	*n. r.*
6^*a*^	LiBr	HCOOH	60 °C	24 h	*n. r.*
7	LiCl	HCOOH	60 °C	12 h	85%
8	LiI	HCOOH	60 °C	12 h	72%
9	LiBr^*b*^	HCOOH^*b*^	60 °C	12 h	27%
10	LiBr^*c*^	HCOOH^*b*^	60 °C	12 h	85%
11	LiBr^*c*^	HCOOH	60 °C	12 h	90%
**12**	**LiBr**	**HCOOH**	**40 °C**	**16 h**	**99%**
13	LiBr	HOAc	40 °C	16 h	27%

^*a*^no Hantzsch ester was used.

^*b*^0.25 eq. was used.

^*c*^2.4 eq. was used. n. r. = no reaction.

As shown in Table 1, the use of LiBr dramatically increases the conversion (entry 1 and 2). The use of weaker Brønsted acid HCOOH increases the conversion up to 86% (entry 3), but is not capable of catalyzing the reaction without Lewis acid (entry 4). As expected, using Lewis acid alone or omitting the Hantzsch ester shuts down the reaction completely (entry 5 and 6). Using LiCl or LiI did not result in an improvement (entry 7 and 8). Optimal results were obtained when 2.4 eq. HE^tBu^ were used^35^ in THF in the presence of 1.0 eq. of lithium bromide and formic acid, respectively, at 40 °C.

**Scheme 4:**
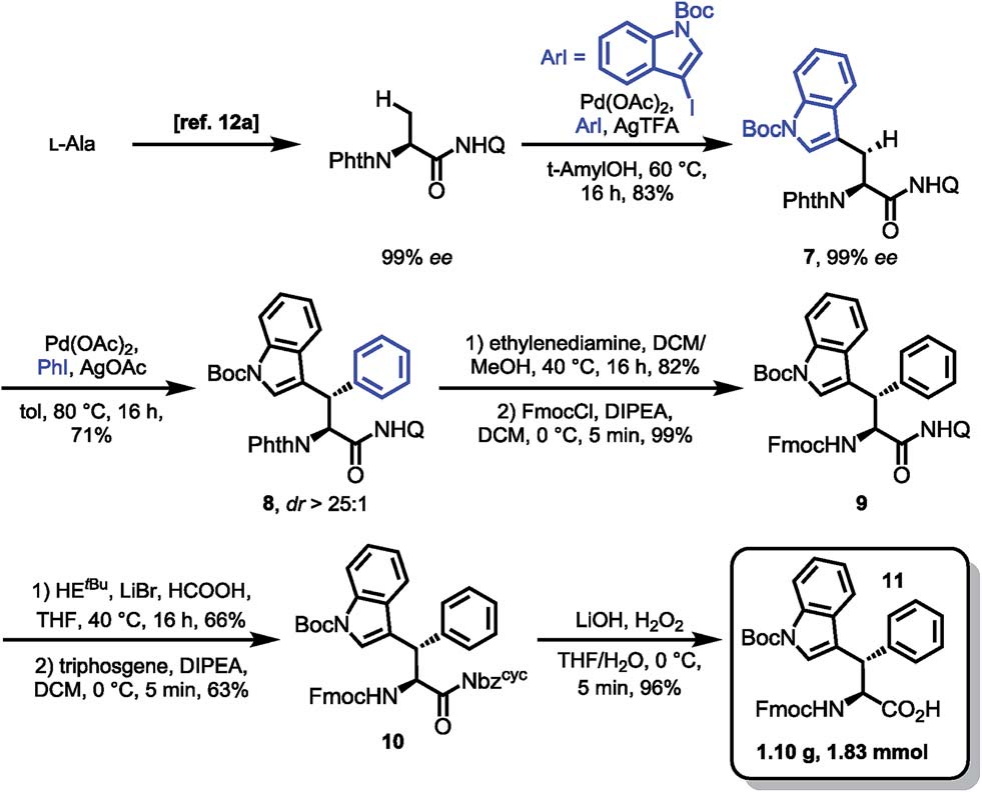
Building block synthesis of Fmoc-Wsf(Boc)-OH (**11**).

With optimized conditions for 8AQ reduction in hand, the arylation of alanine to generate a suitably protected tryptophan was undertaken. Owing to the high steric demand of N-protected 3-iodoindoles, C–H arylation was deemed challenging. However, after screening of silver additives (AgTFA, AgOAc, AgBF_4_, Ag_2_CO_3_ and Ag_2_CO_3_/(BnO)_2_PO_2_H), we found that AgTFA gave a clean indoylation at 60 °C using 10 mol% Pd(OAc)_2_ in *tert*-amyl alcohol to provide 83% of the desired tryptophan **7** with complete retention of the α-stereocenter (99% ee). The methylene arylation proceeded in a substrate-controlled, highly diastereoselective fashion (dr > 25 : 1) and good yields (71%). The presence of an Nα-phthalimide protecting group significantly increased α-epimerization in subsequent reactions. Generally, the manageability of the large-scale synthesis was improved, when the phthalimide protecting group was removed directly after C–H arylation. The phthalimide deprotection was accomplished using excess ethylenediamine to give the free amine in 82% yield. Treatment of the amine with FmocCl resulted in the formation of the Fmoc carbamate **9** in nearly quantitative yield. The following reduction of the quinoline directing group was accomplished using our optimized reaction conditions gave 66% of the tetrahydroquinoline, which was treated with triphosgene to generate the acylurea **10** in 63% yield. With the activated amide in hand, LiOH/H_2_O_2_ mediated hydrolysis was carried out. We were rewarded with the clean amide hydrolysis needing as little as five minutes to give 96% of the target SPPS building block **11** without noteworthy cleavage of the Fmoc protecting group. Remarkably, all the reaction steps of the sequence shown in Scheme 4 could be conducted in gram scale, so that in the end, 1.10 g (1.83 mmol) of the building block was obtained as a white solid, easily storable and ready to use for peptide synthesis.

**Scheme 5:**
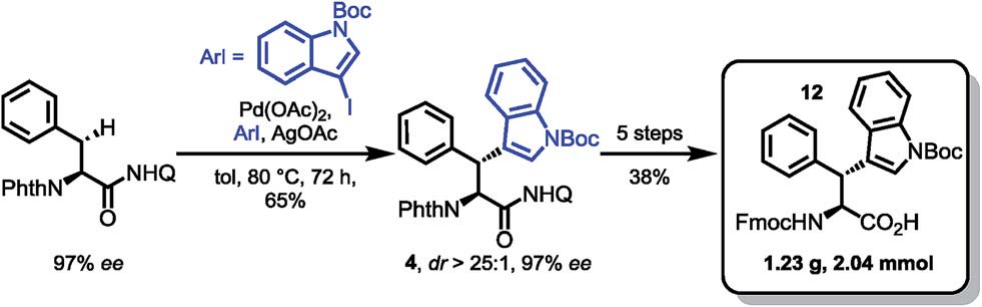
Key arylation and completion of Fmoc-Wrf(Boc)-OH (**12**) synthesis.

For the synthesis of the β-diastereomer, it was necessary to reverse the order of introduction of the aromatic residues,15*b* which was achieved by starting from phenylalanine. The β-indoylation of Phth-Phe-8AQ was expected to be a challenging step, because the aryl halide decomposes at elevated temperature. However, conducting the reaction at temperatures not higher than 80 °C favoured the C–H arylation pathway. At these temperatures, the reaction rate is only moderate and was elevated using as much as five equivalents of the halide. Luckily, the majority of unconsumed halide could be reisolated (98%) and used in further reactions without any loss of efficiency. More importantly, the arylated amino acid **4** could be isolated in 65% yield, again with excellent diastereoselectivity (dr > 25 : 1) and no loss in enantiomeric excess (97% ee). The next steps were conducted in the same manner as for diastereomer **11** shown in Scheme 4 giving yields in comparable ranges. Notably, for this respective diastereomer, the tetrahydroquinoline product was isolated by filtration directly from the reaction mixture in 72% yield. Again, the final hydrolysis step yielded excellent 97% of the SPPS building block Fmoc Wrf(Boc) OH (**12**) (Scheme 5).

**Fig. 2:**
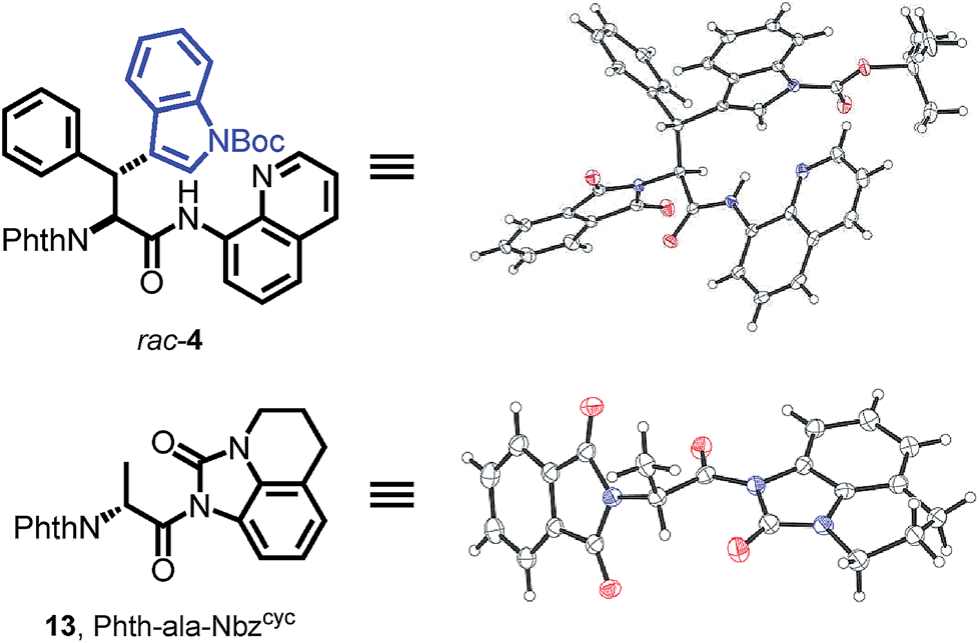
The crystal structures of *rac*-**4** and Phth-ala-Nbz^cyc^ (**13**) show distinct differences concerning amide twist, C–N bond length and nitrogen pyramidalization, explaining the easy removal of Nbz^cyc^.

With both building block syntheses completed, we were interested in elucidating the origin of amidic weakening by our developed method. In a rapidly growing field of cross-coupling^36^ and transamidation chemistry25a,^37^ using activated, nonplanar amides as valuable synthons, we suspected the Nbz^cyc^ to exhibit comparable features. Fortunately, we were able to crystallize a racemic sample of Phth-Wrf(Boc)-8AQ (*rac*-**4**) and d-configured Phth-ala-Nbz^cyc^ (**13**). X-ray analysis of these single crystals (Fig. 2) revealed distinct Winkler–Dunitz parameters^38^ regarding an increased amide twist *τ* of the activated urea (*τ* = 6.9°) compared to the amino-quinoline amide (*τ* = 3.3°). Nitrogen pyramidalization (χN) was found to rise to 11.7° in the activated urea form, whereas χN was found to be 2.3° for the 8AQ amide *rac*-**4**. Furthermore, the respective C–N bond length between the carbon atom of the carbonyl and the nitrogen atom of the directing group was enlarged from 1.35 Å to 1.40 Å in the activated urea form, resulting in a lowered efficiency of amidic resonance.

To gain a profound insight in the induced amidic weakening, theoretical investigations were applied. Szostak *et al.* showed that the resonance energy (*E*_R_) of amides correlates to their reactivity in nucleophilic substitution reactions.^37^,^39^ We therefore calculated the resonance energy for 8AQ amide *rac*-**4** and urea **13**, using crystal structures (Fig. 2) as starting points for calculations. Following the established COSNAR method,^40^ which is readily applied for nonplanar amides,^41^ a decrease of 9.6 kcal mol^–1^ in comparison to the parent aminoquinoline was observed. This decline in resonance energy correlates well to the increase in reactivity towards nucleophilic substitution. Additionally, the bond length from C–N increases while the C=O bond contracts, further supporting the weakening in amidicity.

The Winkler–Dunitz distorsion parameters of the calculated structures are compared to the X-ray structures in Table 2.

**Table 2:** Comparison of Winkler–Dunitz parameters of Phth-Wrf(Boc)-8AQ (*rac*-**4**) and Phth-ala-Nbz^cyc^ (**13**), as well as resonance energies and relative amidicity

Parameter	*rac*-4 (X-ray)	*rac*-4 (Calcd)	Urea 13 (X-ray)	Urea 13 (Calcd)
τ [°]	3.3	3.0	6.9	8.4
χN [°]	2.3	0.3	11.7	6.3
χC [°]	3.1	3.4	1.7	0.9
τ + χN [°]	5.6	3.3	18.6	14.7
C=O [Å]	1.22	1.22	1.21	1.21
C–N [Å]	1.35	1.37	1.40	1.41
*E*_R_ [kcal mol^–1^]	—	16.6	—	7.0
Amidicity^*a*^	—	91%	—	38%

^*a*^Relative to dimethyl acetamide.

**Scheme 6:**
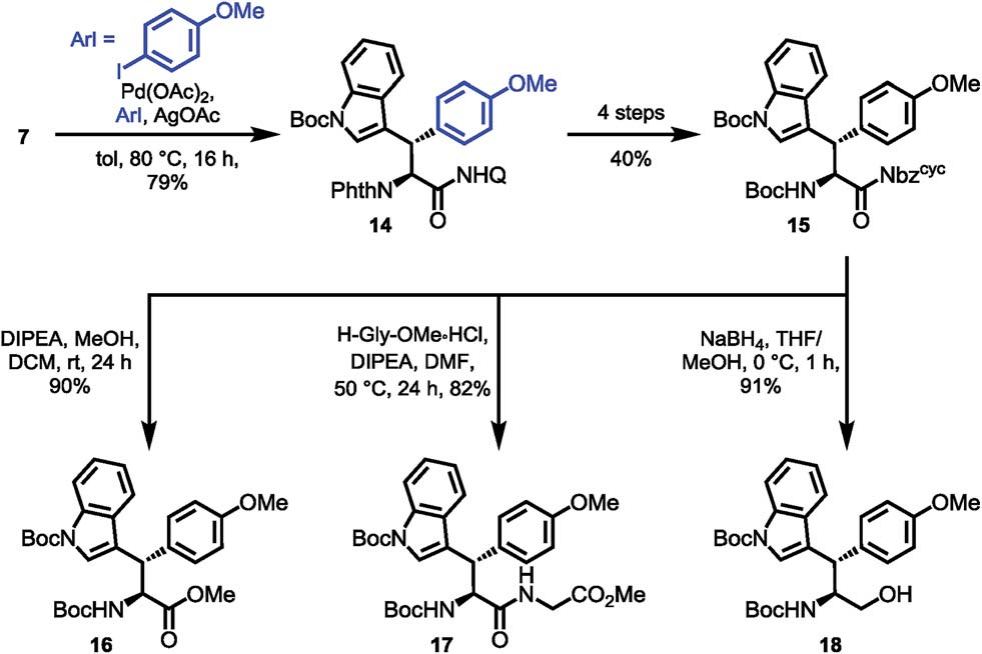
Diverse synthetic applications.

To prove the overall applicability of the concept, we synthesized the chimeric amino acid Boc-Wsy(Boc,Me)-Nbz^cyc^ (**15**) and probed the urea cleavage using nucleophiles for common synthetic precursors. Considering the utility of Dawson's linker,27b,c Nbz^cyc^ ureas could provide rapid access to several carboxylic acid derivatives. The esterification of activated amide **15** using K_2_CO_3_ in MeOH led to a loss of stereointegrity at the acidic α-position (dr 4 : 1), making a milder approach using Hünig's base imperative to give 90% of the methyl ester **16** with no detectable erosion^42^ of the α-stereocenter. Next, we examined the potential of the activated amide **15** to function in an active ester fashion for coupling of dipeptides. The formation of a dipeptide **17** was achieved using glycine methyl ester as the nucleophile in DMF with the aid of Hünig's base at 50 °C for 24 h, which was isolated in 82% yield. Finally, treatment of the activated amide with NaBH_4_ resulted in the clean formation of the protected amino alcohol **18** in 91% yield (Scheme 6).

**Scheme 7:**
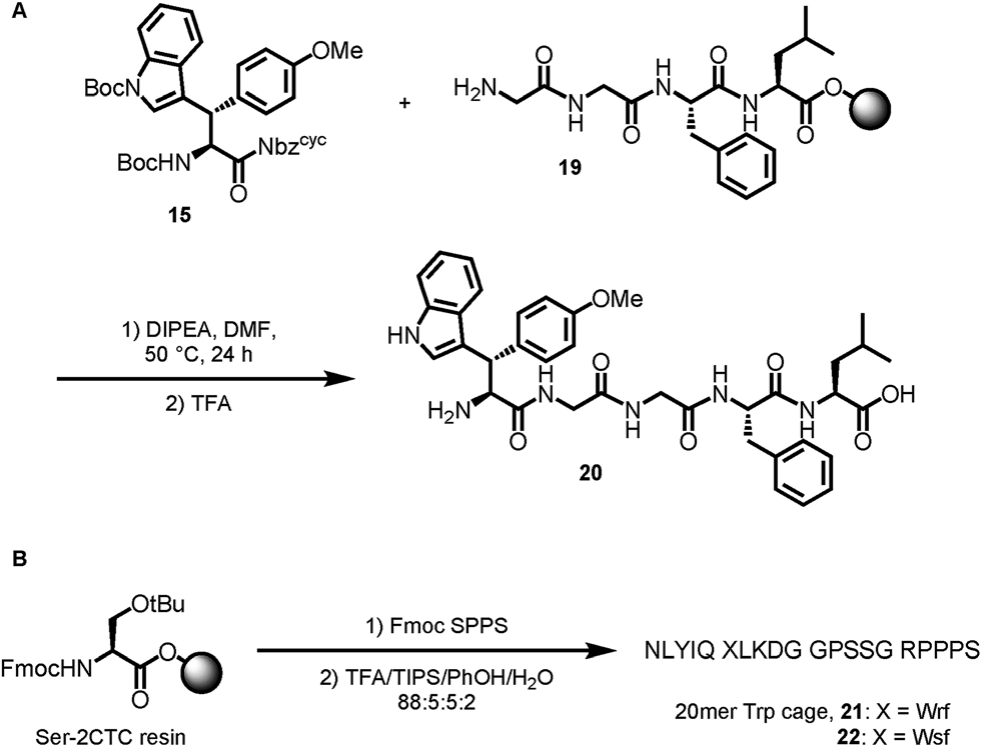
Applications in peptide synthesis.

Based on the results of dipeptide formation, that proved the ureas capability of acylating primary amines, we performed the acylation of a resin-bound Leu-enkephalin precursor **19**. Treatment of the resin-bound peptide with Boc-Wsy(Boc,Me)-Nbz^cyc^ led to the formation of the Leu-enkephalin derivate **20** (Scheme 7A). Furthermore, Fmoc-Wrf(Boc)-OH and Fmoc-Wsf(Boc)-OH were used as a tryptophan substitute to replace the buried Trp^6^ residue in standard automated SPPS to generate two modified 20mer Trp cage (TC5b) mutants **21** and **22** (Scheme 7B).^43^

**Fig. 3:**
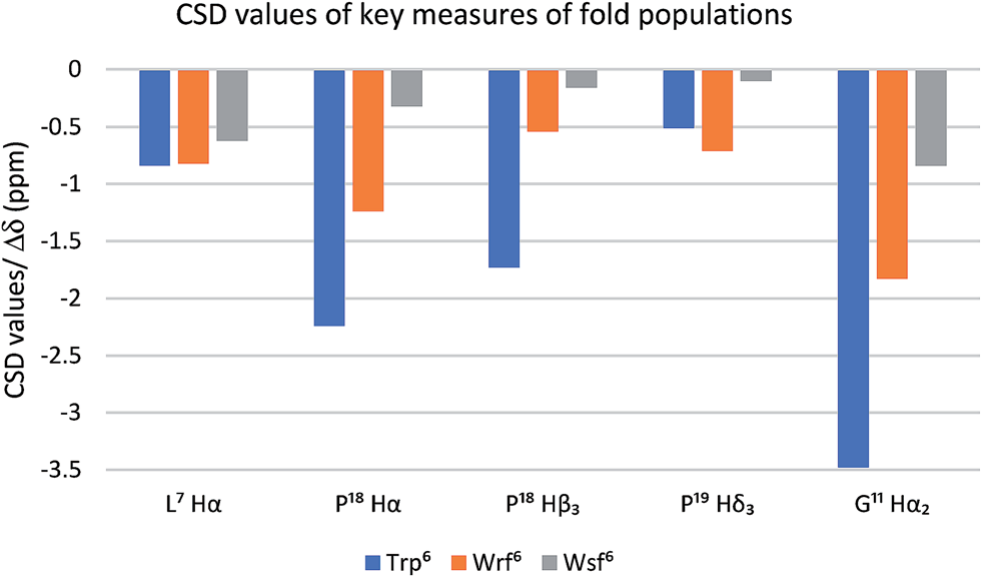
The Trp cage CSD values (blue bars) serve as a quantitative folding reference for the two diastereomeric peptides containing Wrf (orange bars) and Wrf (grey bars).

The Trp cage miniprotein was chosen because the side chain rotamer of the buried Trp^6^ indole group is similar to Wrf. Therefore, we hypothesized that the incorporation of Wrf into TC5b would lead to less disturbance of the densely arranged cage fold. The indole presentation in Wsf, however, should destabilize the cage fold, because the antiperiplanar orientation between Cα–Cβ would force the phenyl ring to occupy the indole cavity in the Trp cage motif. Both diastereomeric peptides **21** and **22** were studied by NMR spectroscopy and compared to the native Trp cage miniprotein TC5b. The chemical shift deviations of key residues for cage fold indication^44^ were used to determine the folded fraction of the two modified miniproteins (Fig. 3).

NOE patterns suggest that the N-terminal α-helical environment is retained for both diastereomers of the peptide. However, according to the CSD values only the Wrf^6^ cage mutant **21** assumes a folded state (75%), whereas the Wsf^6^ mutant **22** shows a Trp cage folding population of only 28%. The strong influence of the restricted side chain mobility and the preferred orientation of the indole moiety on peptide and protein folding is highlighted by these two examples.

## Conclusion

In conclusion, we showed that the close-to-inert amide bond of the widely used directing group 8-aminoquinoline for C–H activation could be addressed *via* amidic weakening through a two-step synthetic procedure, including a highly chemo-selective Hantzsch ester mediated reduction of the pyridyl moiety, followed by urea cyclization. These findings paved the way to the gram-scale synthesis of highly sterically congested β-branched α-amino acids in an orthogonally protected fashion for solid phase peptide synthesis, bridging the rapidly growing fields of directed C–H activation and nonplanar amide chemistry. The altered amide geometry was quantified through X-ray and computational analysis of respective 8-aminoquinoline amides and derived urea compounds to give a better understanding of the induced reactivity in nucleophilic substitution and addition reactions. Ultimately, the generated chimeric amino acids Wrf, Wsf and Wsy were used in solid phase peptide synthesis to give derivatives of Leu-enkephalin and the miniprotein Trp cage, posing the first example of incorporating the optically pure, nonsymmetrical β,β-diaryl alanine motif in peptidic environments. Further investigations on the influence of these amino acids concering the folding and biological activity of peptides are currently under way in our laboratory. We hope that this work will stimulate the use of unsymmetric β-branched α-amino acids to access novel designs of peptide ligands with tailor-made biological features.

## Conflicts of interest

There are no conflicts to declare.

## Supplementary Material

Supplementary information

Crystal structure data
