# Supplementary material for: Directed C(sp3)–H arylation of tryptophan: transformation of the directing group into an activated amide
Source: Chem Sci. 2019 Aug 8;10(37):8634–41. doi: 10.1039/c9sc03440d (PMC6844298; doi:10.1039/c9sc03440d)
Supplement: Supplementary file 1 [file SC-010-C9SC03440D-s001.pdf]

Electronic Supplementary Material (ESI) for Chemical Science.
This journal is © The Royal Society of Chemistry 2019

Supporting Information

Directed C(sp^3) —H arylation of tryptophan: transformation of the directing group into an activated amide

Lennart Nicke, Philip Horx, Klaus Harms, Armin Geyer

Table of Contents

1) List of compounds S2

2) General considerations S3

3) C(sp^3)-H activation S5

4) Phthalimide cleavage: general procedure S12

5) Protection reactions S13

6) Synthesis of tetrahydroquinolines S14

7) Synthesis of Urea compounds (Nbz^{cyc}): general procedure S16

8) Nbz^{cyc} cleavage reactions S18

9) Peptide synthesis S20

10) X-ray analysis S27

11) Computational methods S32

12) NMR data S42

1) List of compounds

HE reduction screening

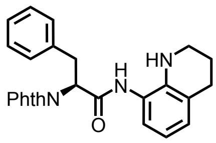

1 (S14)

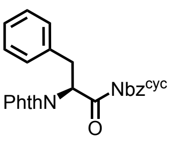

2 (S16)

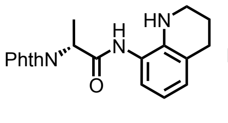

22 (S15)

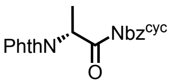

13 (S17)

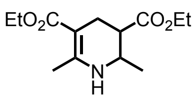

6 (S16)

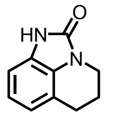

3 (S18)

Fmoc-Wrf(Boc)-OH

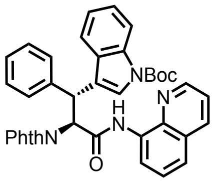

4 (S5)

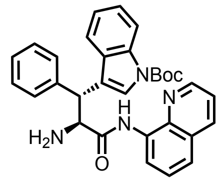

23 (S12)

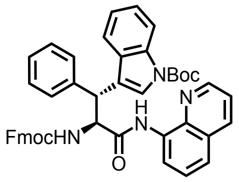

24 (S13)

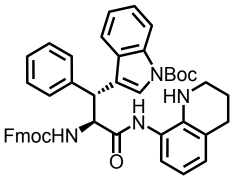

25 (S15)

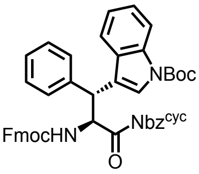

26 (S17)

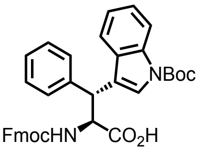

12 (S18)

Fmoc-Wsf(Boc)-OH

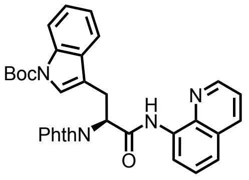

7 (S8)

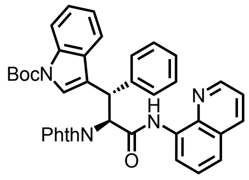

8 (S11)

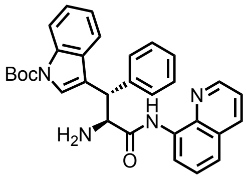

27 (S12)

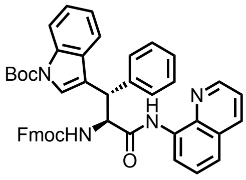

9 (S13)

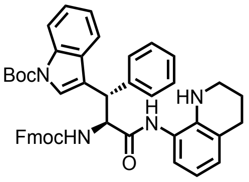

28 (S15)

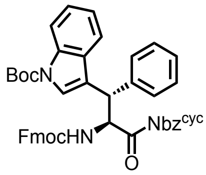

10 (S17)

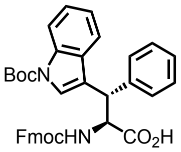

11 (S18)

Boc-Wsy(Boc,Me)-Nbz^{cyc}

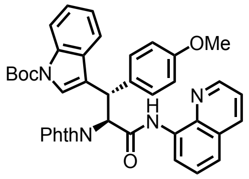

14 (S11)

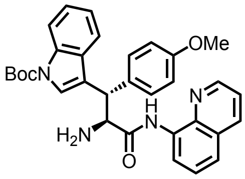

29 (S12)

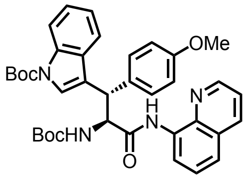

30 (S14)

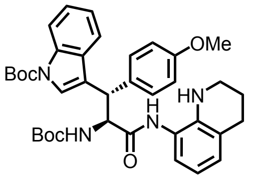

31 (S15)

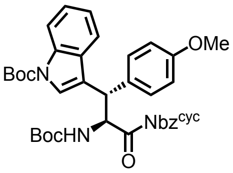

15 (S17)

Cleavage derivatives

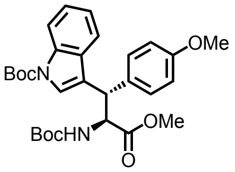

16 (S19)

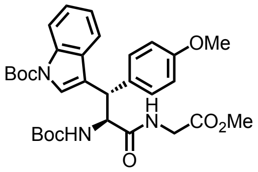

17 (S19)

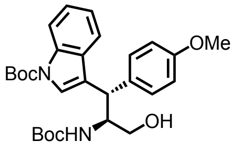

18 (S20)

Leu-Enkephalin derivative and Trp-Cage mutants

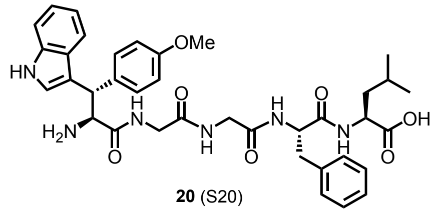

20 (S20)

H-Wsy-Gly-Gly-Phe-Leu-OH

NLYIQ XLKDG GPSSG RPPPS

20mer Trp cage, **21**: X = Wrf (S22)
22: X = Wsf (S24)

2) General considerations

The chemicals used herein were purchased from Sigma-Aldrich and TCI and used without further purification. SPPS building blocks and materials were purchased from Iris Biotech. Starting materials were synthesized according to the attached reference. Reaction solvents were degassed *via* freeze-pump-thaw cycles or by extrusion with argon. THF was distilled over sodium and stored under inert gas conditions with activated 4 Å molecular sieves. Reactions were monitored using crude NMR or TLC. TLC was performed using Merck silica gel TLC 60 F_{254} plates. Staining was performed using UV light and Hannessian´s stain or ninhydrine stain, respectively. Purification of crude products was achieved by column chromatography using silica gel by Macherey-Nagel 60 M (0.040-0.063 mm/ 230-400 mesh). NMR spectra were recorded on Bruker AV II 300, Bruker HD-500, Bruker AV III 500 or Bruker AV II 600 spectrometers. Chemical shifts are reported in ppm using the signal of solvent ($CDCl_3$: ^{1}H: 7.26 ppm, ^{13}C: 77.16 ppm; DMSO-d_6: ^{1}H: 2.50 ppm, ^{13}C: 39.52 ppm) as reference or d_4-trimethylsilylpropanoic acid (^{1}H: 0.00 ppm) for measurements in aqueous media. Multiplicity of signals are described as d = dublet, t = triplet, q = quartet, quint = quintet, oct = octet, br = broadened signal. ^{13}C shifts for peptides were extracted from the HSQC spectrum. Mass spectra (ESI+) were acquired on a Thermo Fisher Scientific LTQ-FT. Peptides were synthesized automatically on a Liberty Blue peptide synthesizer. Analytical HPLC was performed on a Thermo Scientific Dionex UltiMate 3000 system with an ACE UltraCore 2.5 SuperC18 column (150 x 2.1 mm). As eluent with a flow rate of 0.45 mL/min were used the following solvents: A: H_2O + 0,1% TFA and B: MeCN + 0,085% TFA. Semi preparative HPLC was performed on a Thermo Scientific Dionex UltiMate 3000 with a Macherey-Nagel VP Nucleodur C18 Gravity column with a flow rate of 15.0 mL/min. Diastereomeric ratios (*dr*) were determined using NMR spectroscopy, enantiomeric excess (*ee*) was determined using a JASCO HPLC system with Chiralpak IA column and *n*-hexane/*i*PrOH (HPLC grade) as eluent. Optical rotations were determined with an A:KRÜSS Optronic P8000-T polarimeter.

3) C(sp^3)-H activation

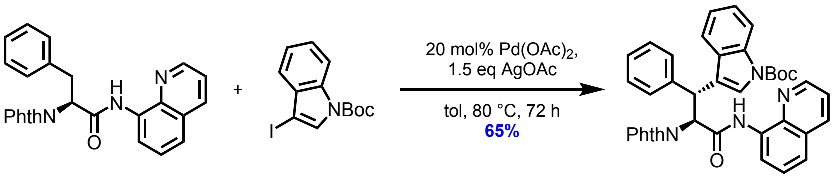

4

A pressure flask was charged with 6.00 g (14.2 mmol, 1.0 eq) Phth-Phe-8AQ (97% *ee*) (*Org. Lett.* **2006**, *8 (15)*, 3391-3394), 24.4 g (71.2 mmol, 5.0 eq) *N*-Boc-3-iodoindole (*Eur. J. Org. Chem.* **2013**, 4564-4569), 639 mg (2.85 mmol, 20 mol%) $Pd(OAc)_2$ and 3.56 g (21.4 mmol, 1.5 eq) AgOAc. The flask was flushed with Ar. To this was added 12.0 mL of anhydrous toluene and the mixture was vigorously stirred at 80 °C for 72 h. After completion, the mixture was diluted with DCM and filtered through a pad of celite. The residue was concentrated and purified by column chromatography on silica using toluene/ethyl acetate to furnish 5.86 g (9.20 mmol, 65%) of compound **4** (*dr* > 25:1, 97% *ee*) as a white solid among with 19.4 g (56.5 mmol, 79%) of reisolated aryl halide as a pale brown oil.

$[a]_D^{25}$ = -131.3° ($CHCl_3$, c = 1.0); ^{1}H-NMR (500 MHz, $CDCl_3$), δ = 10.47 (brs, 1H), 8.65-8.61 (m, 1H), 8.26 (dd, 1H, J = 4.2 Hz, 1.6 Hz, 1H), 8.13-8.03 (brd, 1H), 8.01 (dd, J = 8.3 Hz, 1.7 Hz), 7.97 (s, 1H), 7.77-7.73 (m, 2H), 7.72 (dt, J = 7.87 Hz, 0.8 Hz, 1H), 7.65-7.60 (m, 2 H), 7.46-7.42 (m, 2H), 7.42-7.38 (m, 2H), 7.27 (dd, J = 8.3 Hz, 4.3 Hz, 1H), 7.23 (ddd, J = 8.2 Hz, 7.3 Hz, 1.1 Hz, 1H), 7.17-7.10 (m, 3H), 7.03-6.98 (m, 1H), 5.97 (d, J = 11.9 Hz, 1H), 5.74 (d, J = 12.0 Hz, 1H), 1.62 (s, 9H) ppm. ^{13}C-NMR (125 MHz, $CDCl_3$), δ = 168.0, 165.7, 149.9, 147.8, 139.8, 138.3, 138.28, 136.26, 136.0, 134.1, 131.6, 130.0, 128.6, 128.3, 127.8, 127.28, 127.25, 124.9, 123.5, 122.8, 122.0, 121.5, 121.1, 119.7, 117.3, 116.5, 115.2, 84.1, 58.2, 41.8 ppm. HRMS (ESI+) Calcd for $C_{39}H_{32}N_4O_5Na$ $[M+Na]^+$: 659.2265, found: 659.2275. HPLC (Chiralpak IA, *n*-hexane/*i*PrOH 50:50, 0.5 mL/min): t_r = 13.308 min (major), 16.833 (minor), *ee*: 97%.

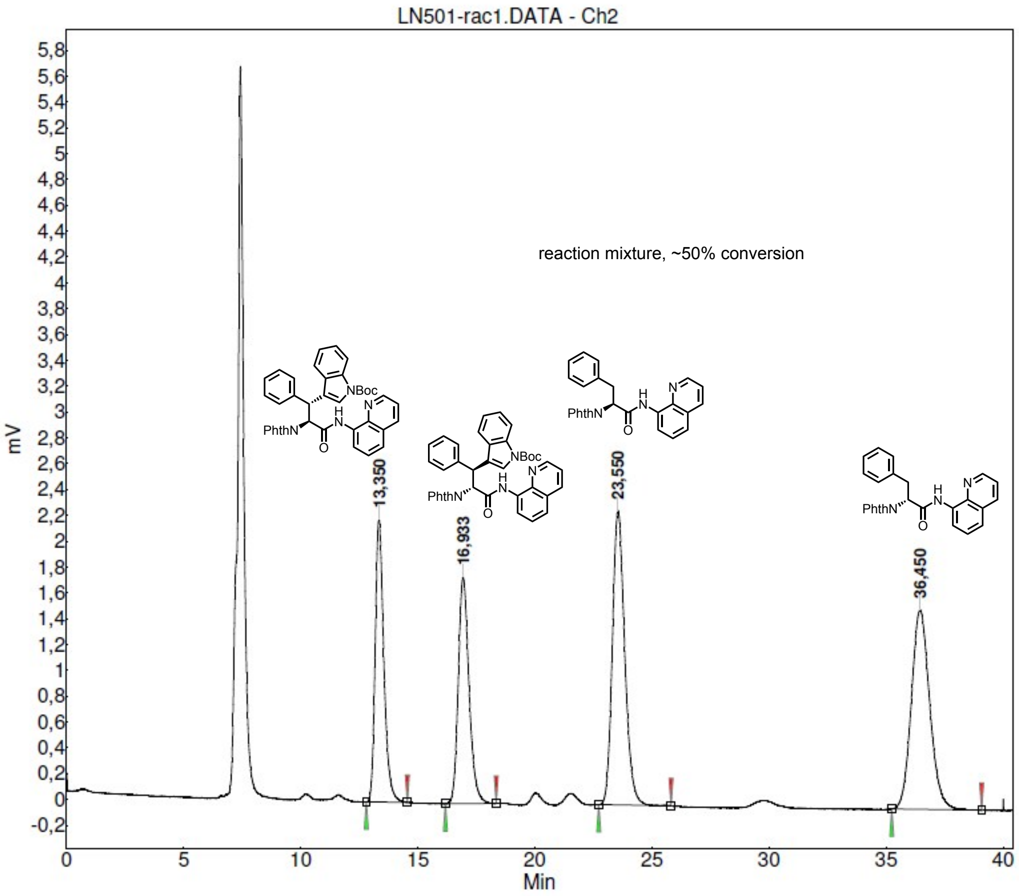

Peak results :

Index	Name	Time [Min]	Quantity [% Area]	Height [mV]	Area [mV.Min]	Area % [%]
1	UNKNOWN	13,350	20,07	2,2	0,9	20,072
2	UNKNOWN	16,933	19,98	1,8	0,9	19,979
3	UNKNOWN	23,550	29,89	2,3	1,4	29,894
4	UNKNOWN	36,450	30,06	1,5	1,4	30,056
Total			100,00	7,8	4,7	100,000

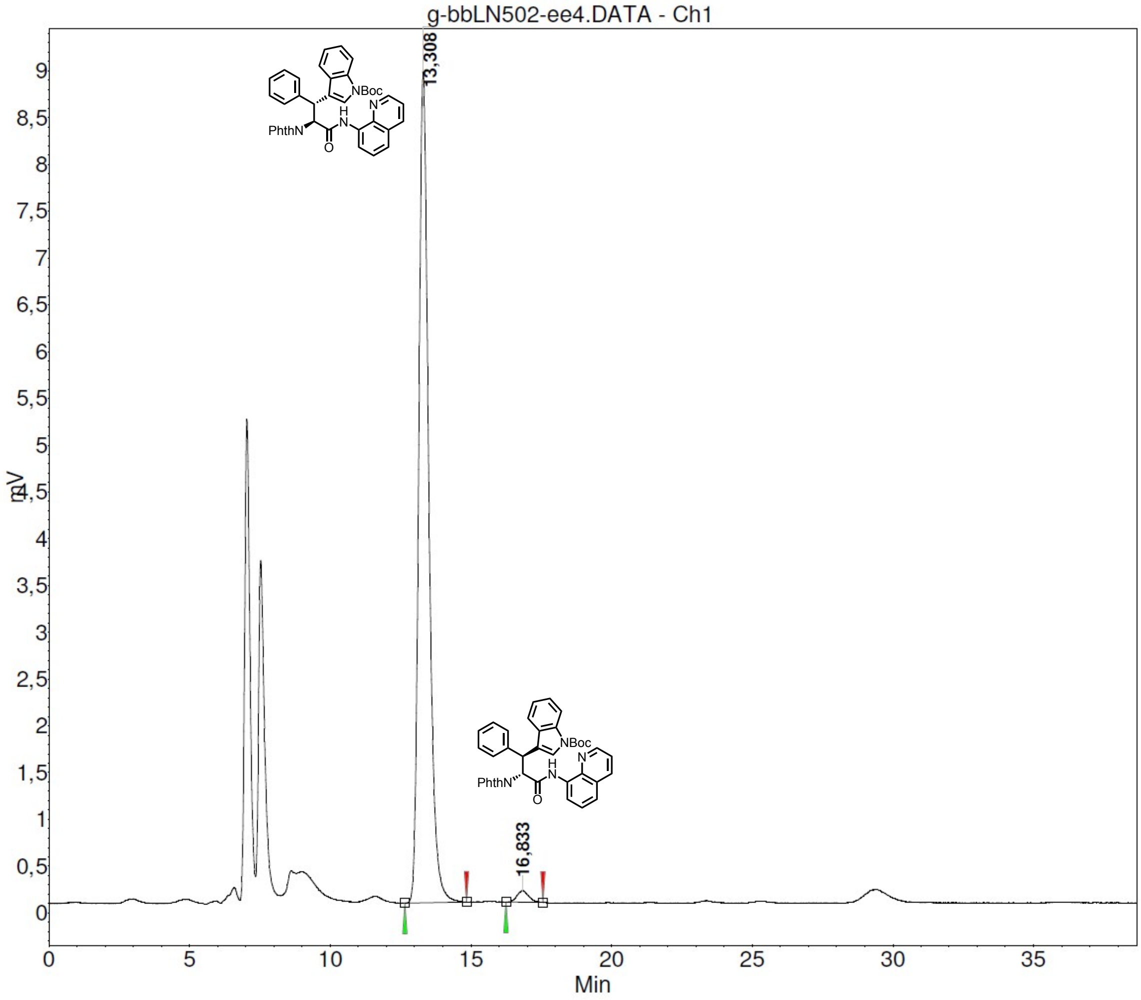

Peak results :

Index	Name	Time [Min]	Quantity [% Area]	Height [mV]	Area [mV.Min]	Area % [%]
1	UNKNOWN	13,308	98,39	8,9	3,6	98,392
2	UNKNOWN	16,833	1,61	0,1	0,1	1,608
Total			100,00	9,0	3,7	100,000

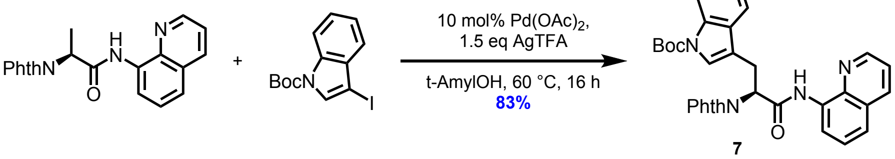

7

A pressure flask was charged with 4.00 g (11.6 mmol, 1.0 eq) Phth-Ala-8AQ (99% *ee*) (*Org. Lett.* **2006**, *8 (15)*, 3391-3394), 5.96 g (17.4 mmol, 1.5 eq) *N*-Boc-3-iodoindole (*Eur. J. Org. Chem.* **2013**, 4564-4569), 260 mg (1.16 mmol, 10 mol%) $Pd(OAc)_2$ and 3.84 g (17.4 mmol, 1.5 eq) AgTFA. The flask was flushed with Ar. To this was added 55.0 mL of degassed *tert*-amyl alcohol and the mixture was vigorously stirred at 60 °C for 16 h. After completion, the mixture was diluted with DCM and 3.21 mL (23.2 mmol, 2 eq) NEt_3 was added. The mixture was stirred for additional 30 minutes and filtered through a pad of celite. Volatiles were removed under reduced pressure. The crude material was dissolved in DCM and washed with 1 M aq. $NaHCO_3$ solution and *brine*. The organic layer was dried over $MgSO_4$, filtered and concentrated. The residue was purified by column chromatography on silica using toluene/ethyl acetate to furnish 5.41 g (9.65 mmol, 83%) of compound **7** (99% *ee*) as a white solid.

^{1}H-NMR (500 MHz, $CDCl_3$), δ = 10.29 (s, 1H), 8.73 (dd, J = 7.0 Hz, 2.0 Hz, 1H), 8.55 (dd, J = 4.3 Hz, 1.6 Hz, 1H), 8.16-8.09 (m, 2H), 7.89-7.84 (m, 2H), 7.75-7.72 (m, 2H), 7.69 (d, J = 7.6 Hz, 1H), 7.55 (brs, 1H), 7.54-7.47 (m, 2H), 7.37 (dd, J = 8.3 Hz, 4.3 Hz, 1H), 7.33-7.28 (m, 1H), 7.25-7.23 (m, 1H), 5.59 (dd, J = 8.7 Hz, 7.0 Hz, 1H), 4.01 (ddd, J = 15.1 Hz, 6.9 Hz, 0.9 Hz, 1H), 3.81 (ddd, J = 15.1 Hz, 8.8 Hz, 0.9 Hz, 1H), 1.58. (s, 9H) ppm. ^{13}C-NMR (125 MHz, $CDCl_3$), δ = 168.1, 166.5, 149.6, 148.2, 138.3, 136.6, 135.9, 134.3, 133.9, 132.0, 130.1, 128.0, 127.5, 124.8, 124.6, 123.7, 122.9, 122.1, 121.7, 119.1, 117.2, 116.0, 115.4, 83.6, 54.6, 28.3, 25.4 ppm. HRMS (ESI+) Calcd for $C_{33}H_{28}N_4O_5H$ $[M+H]^+$: 561.2132, found: 561.2134. HPLC (Chiralpak IA, *n*-hexane/*i*PrOH 50:50, 0.5 mL/min): t_r = 19.292 min (major), 28.108 (minor), *ee*: 99%.

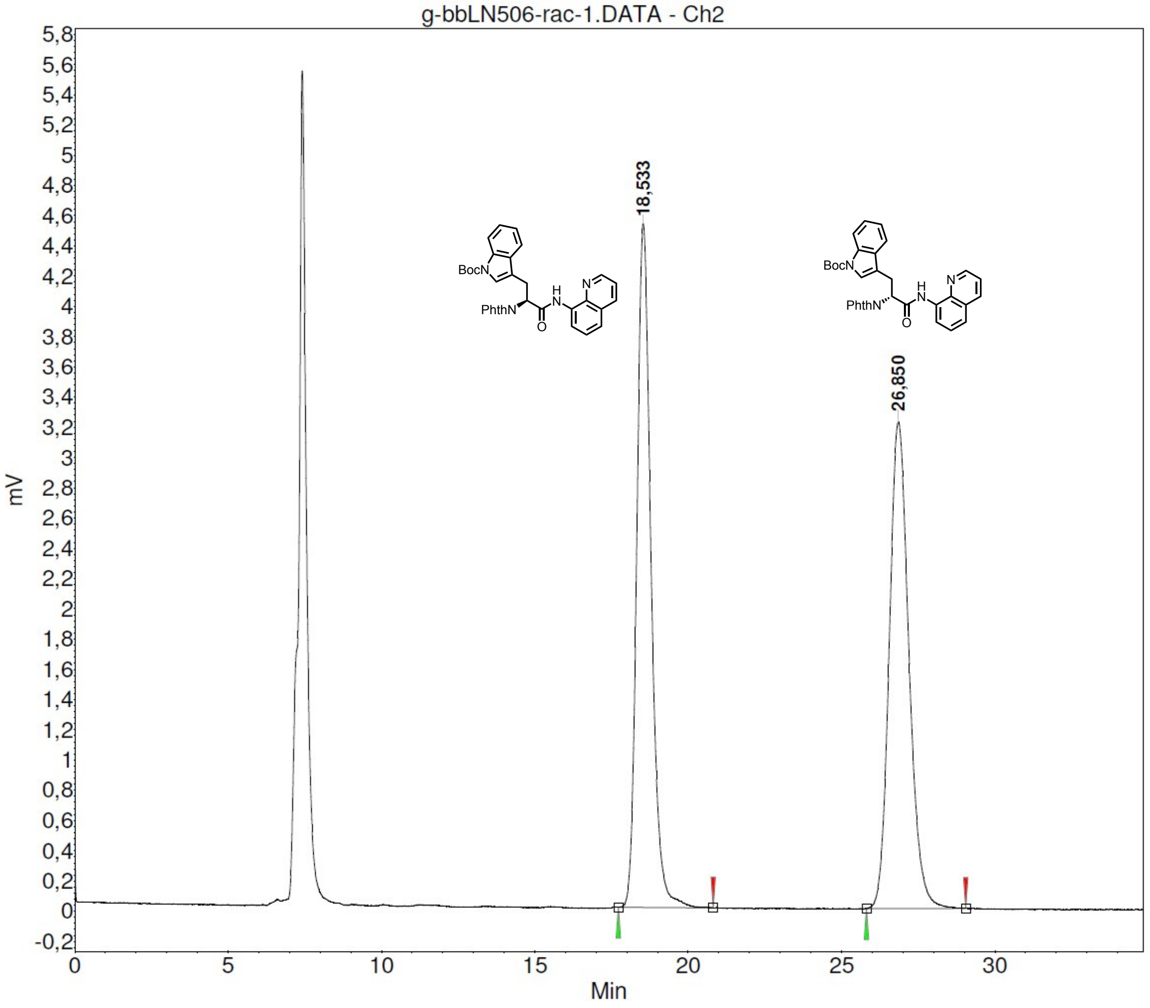

Peak results :

Index	Name	Time [Min]	Quantity [% Area]	Height [mV]	Area [mV.Min]	Area % [%]
1	UNKNOWN	18,533	50,31	4,5	2,4	50,311
2	UNKNOWN	26,850	49,69	3,2	2,4	49,689
Total			100,00	7,7	4,8	100,000

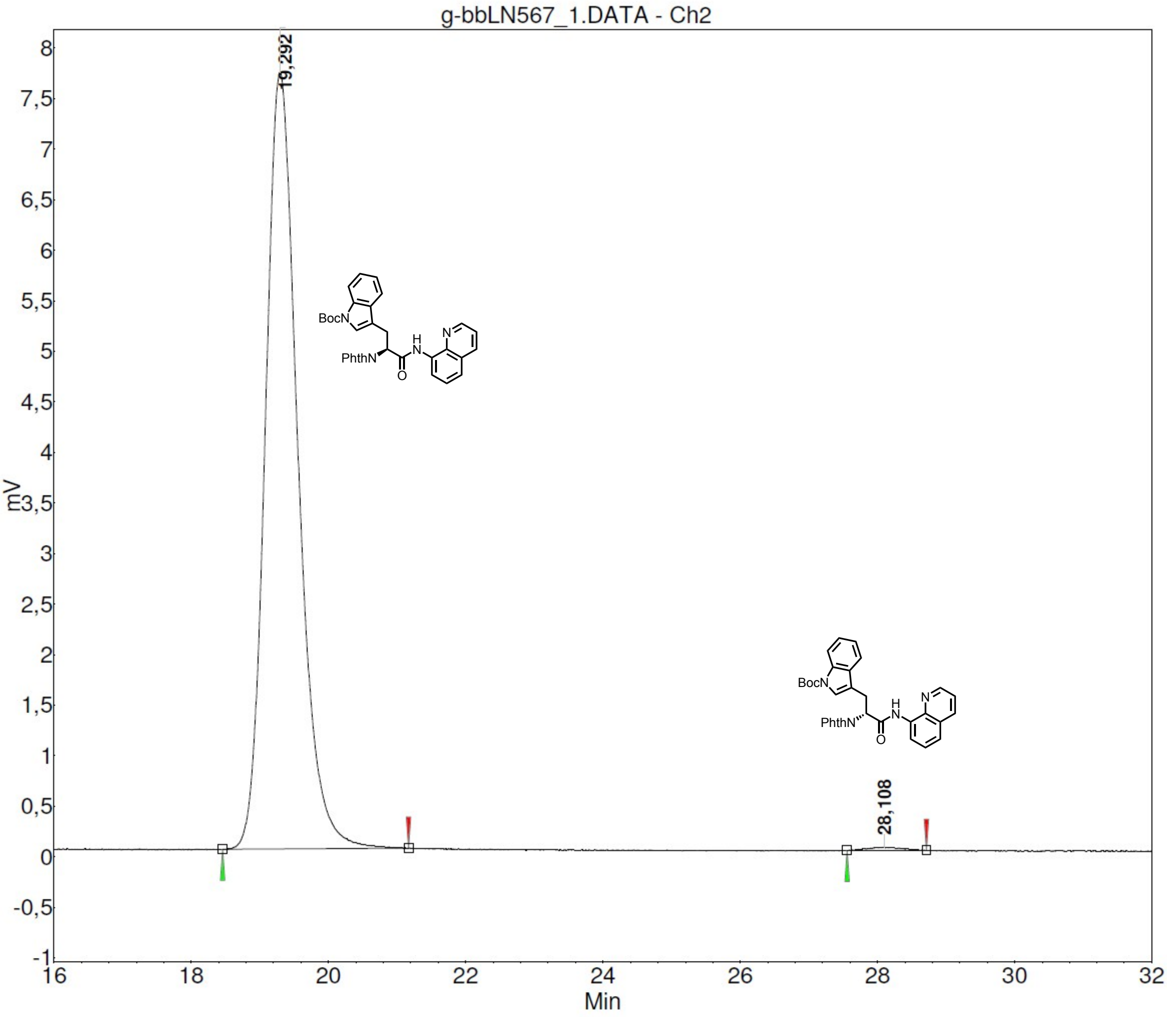

Peak results :

Index	Name	Time [Min]	Quantity [% Area]	Height [mV]	Area [mV.Min]	Area % [%]
1	UNKNOWN	19,292	99,60	7,7	4,2	99,599
2	UNKNOWN	28,108	0,40	0,0	0,0	0,401
Total			100,00	7,7	4,2	100,000

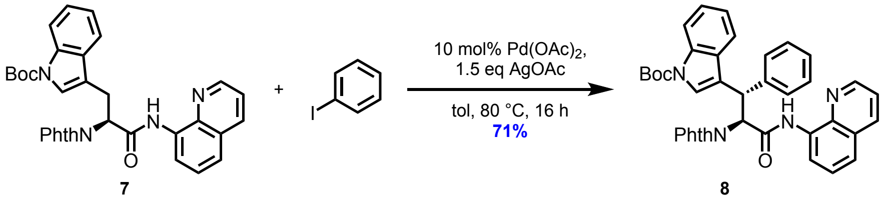

A pressure flask was charged with 5.00 g (8.92 mmol, 1.0 eq) Phth-Trp(Boc)-8AQ (**7**), 4.0 mL (35.7 mmol, 4.0 eq) iodobenzene, 200 mg (0.89 mmol, 10 mol%) $Pd(OAc)_2$ and 2.23 g (13.4 mmol, 1.5 eq) AgOAc. The flask was flushed with Ar. To this was added 5.0 mL of anhydrous toluene and the mixture was vigorously stirred at 80 °C for 16 h. After completion, the mixture was diluted with DCM and filtered through a pad of celite. The residue was concentrated and purified by column chromatography on silica using toluene/ethyl acetate to furnish 4.04 g (6.35 mmol, 71%) of compound **8** (*dr* > 25:1) as a white solid.

^{1}H-NMR (500 MHz, $CDCl_3$), δ = 10.16 (s, 1H), 8.76 (dd, J = 4.2 Hz, 1.6 Hz, 1H), 8.61 (dd, J = 6.7 Hz, 2.2 Hz, 1H), 8.10 (dd, J = 8.3 Hz, 1.5 Hz, 1H), 8.00 (d, J = 7.5 Hz, 1H), 7.81-7.74 (m, 2H), 7.67-7.61 (m, 4H), 7.59 (d, J = 7.8 Hz, 1H), 7.48-7.39 (m, 3H), 7.24-7.17 (m, 3H), 7.16-7.12 (m, 1H), 7.07-7.03 (m, 1H), 6.04 (d, J = 12.2 Hz, 1H), 5.91 (d, J = 12.2 Hz, 1H), 1.62 (s, 9H) ppm. ^{13}C-NMR (125 MHz, $CDCl_3$), δ = 168.1, 165.8, 149.6, 148.0, 139.6, 138.2, 136.7, 135.6, 134.2, 133.9, 131.7, 129.7, 129.1, 128.8, 127.9, 127.5, 127.4, 124.6, 123.7, 122.7, 122.13, 122.07, 121.6, 120.6, 119.7, 117.6, 115.0, 83.7, 59.7, 41.6, 28.3 ppm. HRMS (ESI+) Calcd for $C_{39}H_{32}N_4O_5H$ $[M+H]^+$: 637.2445, found: 637.2471.

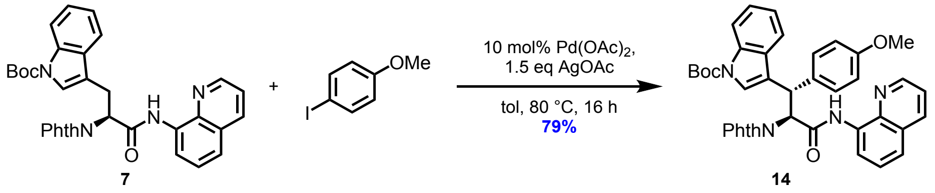

A pressure flask was charged with 2.00 g (3.59 mmol, 1.0 eq) Phth-Trp(Boc)-8AQ, 3.34 g (14.3 mmol, 4.0 eq) 4-iodoanisole, 80.1 mg (0.36 mmol, 10 mol%) $Pd(OAc)_2$ and 0.89 g (5.35 mmol, 1.5 eq) AgOAc. The flask was flushed with Ar. To this was added 2.0 mL of anhydrous toluene and the mixture was vigorously stirred at 80 °C for 16 h. After completion, the mixture was diluted with DCM and filtered through a pad of celite. The residue was concentrated and purified by column chromatography on silica using toluene/ethyl acetate to furnish 1.79 g (2.81 mmol, 79%) of compound **14** (*dr* > 25:1) as a white solid.

^{1}H-NMR (500 MHz, $CDCl_3$), δ = 10.17 (s, 1H), 8.77 (dd, J = 4.3 Hz, 1.6 Hz, 1H), 8.63 (dd, J = 6.7 Hz, 2.3 Hz, 1H), 8.15 (d, J = 8.1 Hz, 1H), 8.04-7.97 (m, 1H), 7.81-7.75 (m, 2H), 7.66-7.63 (m, 2H), 7.62 (s, 1H), 7.57 (d, J = 7.8 Hz, 1H), 7.55-7.50 (m, 2H), 7.50-7.42 (m, 3H), 7.23-7.17 (m, 1H), 7.15-7.11 (m, 1H), 6.73-6.69 (m, 2H), 5.99 (d, J = 12.2 Hz, 1H), 5.87 (d, J = 12.2 Hz, 1H), 3.56 (s, 3H), 1.61 (s, 9H) ppm. ^{13}C-NMR (125 MHz, $CDCl_3$), δ = 168.1, 166.1, 158.9, 149.6, 147.7, 137.8, 137.1, 135.6, 134.2, 133.8, 131.7, 131.5, 129.9, 129.6, 128.0, 127.6, 124.6, 123.7, 122.7, 122.2, 121.8, 121.6, 120.8, 119.7, 118.0, 115.0, 114.5, 83.6, 59.9, 55.1, 40.8, 28.3 ppm. HRMS (ESI+) Calcd for $C_{40}H_{34}N_4O_6H$ $[M+H]^+$: 667.2551, found: 667.2557.

4) Phthalimide cleavage: general procedure

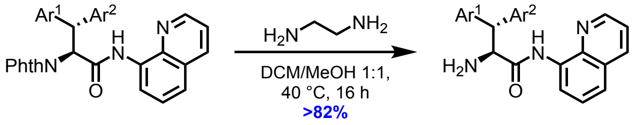

In a pressure flask, the phthalimide-protected amino acid (1.0 eq) was dissolved in dichloromethane/methanol (1:1, final concentration 0.1 M) and ethylenediamine (5.0 eq) was added. The flask was tightly sealed and stirred at 40 °C for 16 h. After completion, the mixture was allowed to cool to rt and volatiles were removed under reduced pressure. The residue was purified by column chromatography on silica using dichloromethane/methanol to give the free amine in high yield (>82%).

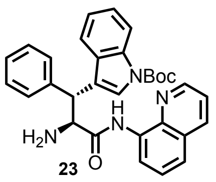

4.00 g, 7.89 mmol, 88%, white solid. ^{1}H-NMR (500 MHz, DMSO), δ = 11.39 (s, 1H), 8.86 (dd, J = 4.2 Hz, 1.7 Hz, 1H), 8.69 (dd, J = 7.7 Hz, 1.3 Hz, 1H), 8.37 (dd, J = 8.3 Hz, 1.7 Hz, 1H), 8.00-7.96 (m, 2H), 7.63 (dd, J = 8.3 Hz, 1.3 Hz, 1H), 7.59 (dd, J = 8.3 Hz, 4.2 Hz, 1H), 7.55 (t, J = 7.9 Hz, 1H), 7.36-7.33 (m, 2H), 7.27-7.20 (m, 4H), 7.16-7.12 (m, 1H), 7.10-7.06 (m, 1H), 4.97 (d, J = 4.9 Hz, 1H), 4.44 (d, J = 3.5 Hz, 1H), 2.28 (brs, 2H), 1.60 (s, 9H) ppm. ^{13}C-NMR (125 MHz, DMSO), δ = 172.9, 149.2, 148.9, 139.3, 138.1, 136.4, 134.6, 134.1, 129.9, 129.0, 128.3, 127.8, 126.9, 126.7, 124.3, 123.4, 122.31, 122.0, 121.7, 121.5, 119.6, 115.6, 114.7, 83.6, 58.6, 44.7, 27.7 ppm. HRMS (ESI+) Calcd for $C_{31}H_{30}N_4O_3H$ $[M+H]^+$: 507.2391, found: 507.2405.

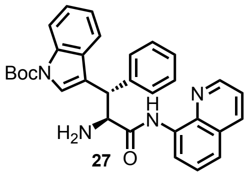

2.60 g, 5.12 mmol, 82%, white solid. ^{1}H-NMR (500 MHz, DMSO), δ = 11.67 (s, 1H), 8.92 (dd, J = 4.1 Hz, 1.7 Hz, 1H), 8.63 (dd, J = 7.7 Hz, 1.3 Hz, 1H), 8.38 (dd, J = 8.3 Hz, 1.7 Hz, 1H), 7.96 (d, J = 8.2 Hz, 1H), 7.86 (s, 1H), 7.64-7.60 (m, 2H), 7.52 (t, J = 8.0 Hz, 1H), 7.44-7.40 (m, 2H), 7.30-7.25 (m, 2H), 7.25-7.20 (m, 2H), 7.20-7.16 (m, 1H), 7.09-7.05 (m, 1H), 4.99 (d, J = 4.9 Hz Hz, 1H), 4.24 (d, J = 4.6 Hz, 1H), 2.41 (brs, 2H), 1.38 (s, 9H) ppm. ^{13}C-NMR (125 MHz, DMSO), δ = 172.4, 148.93, 148.87, 141.3, 138.2, 136.5, 134.6, 134.1, 130.2, 128.5, 128.3, 127.8, 126.9, 126.6, 124.3, 123.6, 122.3, 122.0, 121.6, 119.7, 119.0, 115.3, 114.5, 83.3, 60.1, 45.2, 27.4 ppm. HRMS (ESI+) Calcd for $C_{31}H_{30}N_4O_3H$ $[M+H]^+$: 507.2391, found: 507.2406.

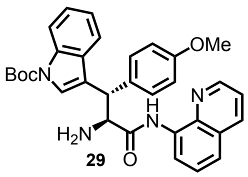

1.18 g, 2.20 mmol, 86%, white solid. ^{1}H-NMR (500 MHz, DMSO), δ = 11.65 (s, 1H), 8.92 (dd, J = 4.2 Hz, 1.7 Hz, 1H), 8.63 (dd, J = 7.7 Hz, 1.3 Hz, 1H), 8.38 (dd, J = 8.4 Hz, 1.7 Hz, 1H), 7.95 (d, J = 8.2 Hz, 1H), 7.83 (s, 1H), 7.65-7.60 (m, 2H), 7.52 (t, J = 8.0 Hz, 1H), 7.34-7.29 (m, 2H), 7.25-7.20 (m, 2H), 7.10-7.05 (m, 1H), 6.85-6.81 (m, 2H), 4.93 (d, J = 4.9 Hz, 1H), 4.18 (d, J = 4.8 Hz, 1H), 3.67 (s, 3H), 2.39 (brs, 2H), 1.37 (s, 9H) ppm. ^{13}C-NMR (125 MHz, DMSO), δ = 172.5, 157.9, 148.92, 148.88, 138.2, 136.4, 134.6, 134.1, 133.1, 130.2, 129.5, 127.8, 126.9, 124.2, 123.4, 122.3, 122.0, 121.6, 119.8, 119.3, 115.3, 114.5, 113.7, 83.3, 60.3, 54.9, 44.4, 27.4 ppm. HRMS (ESI+) Calcd for $C_{32}H_{32}N_4O_4H$ $[M+H]^+$: 537.2496, found: 537.2505.

5) Protection reactions

Fmoc protection: general procedure

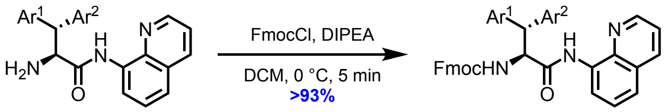

The amine (1.0 eq) was dissolved in dichloromethane (3/4 of final volume) and cooled to 0 °C. DIPEA (2.0 eq) was added and a solution of FmocCl (1.05 eq) in dichloromethane (1/4 of final volume, final concentration 0.1 M) was added dropwise. After complete addition, the mixture was stirred at 0 °C for five minutes to reach completion. Saturated NH_4Cl solution was added and the mixture was extracted with dichloromethane. The organic layer was washed with brine, dried over $MgSO_4$ and filtered. Volatiles were removed under reduced pressure and the residue was purified by column chromatography on silica using toluene/ethyl acetate to give the respective Fmoc-carbamate in high yield (>93%).

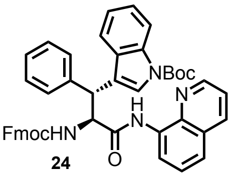

24

5.29 g, 7.26 mmol, 93%, white solid. ^{1}H-NMR (500 MHz, $CDCl_3$), δ = 10.21 (s, 1H), 8.74 (dd, J = 7.1 Hz, 1.8 Hz, 1H), 8.57 (d, J = 2.9 Hz, 1H), 8.11 (dd, J = 8.2 Hz, 1.2 Hz, 1H), 8.04 (d, J = 5.5 Hz, 1H), 7.87 (s, 1H), 7.76 (d, J = 7.2 Hz, 2H), 7.57 (d, J = 7.4 Hz, 1H), 7.55-7.48 (m, 3H), 7.44-7.33 (m, 5H), 7.33-7.27 (m, 2H), 7.26-7.22 (m, 4H), 7.21-7.16 (m, 1H), 7.07-7.02 (m, 1H), 5.59 (d, J = 7.7 Hz, 1H), 5.44 (t, J = 7.6 Hz, 1H), 5.17 (d, J = 5.0 Hz, 1H), 4.46-4.35 (m, 2H), 4.26 (t, J = 7.3 Hz, 1H), 1.60 (s, 9H) ppm. ^{13}C-NMR (125 MHz, $CDCl_3$), δ = 169.1, 156.2, 149.7, 148.2, 144.0, 143.9, 141.40, 141.37, 138.5, 138.4, 136.4, 135.8, 133.8, 130.0, 129.1, 128.0, 127.9, 127.8, 127.7, 127.4, 127.21, 127.20, 125.34, 125.27, 124.6, 123.5, 122.6, 122.2, 121.7, 120.11, 120.06, 119.8, 117.0, 115.1, 83.6, 67.7, 59.3, 47.3, 44.7, 28.3 ppm. HRMS (ESI+) Calcd for $C_{46}H_{40}N_4O_5H$ $[M+H]^+$: 729.3071, found: 729.3088.

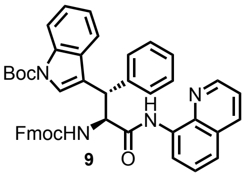

9

3.59 g, 4.93 mmol, 99%, white solid. ^{1}H-NMR (500 MHz, $CDCl_3$), δ = 9.95 (s, 1H), 8.69 (dd, J = 7.2 Hz, 1.7 Hz, 1H), 8.58 (d, J = 3.3 Hz, 1H), 8.24-8.13 (m, 2H), 7.78-7.70 (m, 3H), 7.66 (s, 1H), 7.59-7.50 (m, 3H), 7.46 (d, J = 7.1 Hz, 1H), 7.42 (d, J = 7.6 Hz, 2H), 7.40-7.29 (m, 3H), 7.25-7.17 (m, 5H), 7.10 (t, J = 7.4 Hz, 1H), 6.01 (brs, 1H), 5.43 (t, J = 7.8 Hz, 1H), 5.03 (d, J = 7.5 Hz, 1H), 4.52-4.45 (m, 1H), 4.32-4.24 (m, 2H), 1.52 (s, 9H) ppm. ^{13}C-NMR (125 MHz, $CDCl_3$), δ = 168.9, 156.2, 149.7, 147.3, 144.0, 143.9, 141.39, 141.35, 138.7, 137.6, 136.1, 133.3, 130.0, 128.91, 128.85, 128.1, 127.81, 127.75, 127.5, 127.2, 125.4, 125.3, 124.9, 124.3, 123.0, 122.3, 121.5, 120.1, 120.0, 119.9, 119.3, 118.2, 115.4, 83.7, 67.5, 59.8, 47.2, 46.2, 28.2 ppm. HRMS (ESI+) Calcd for $C_{46}H_{40}N_4O_5H$ $[M+H]^+$: 729.3071, found: 729.3084.

Boc Protection

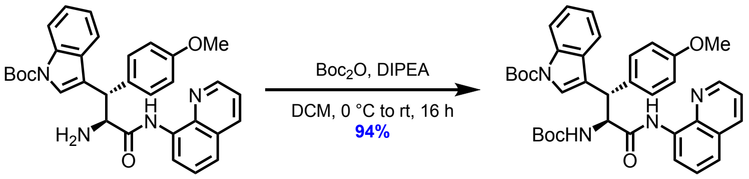

1.15 g (2.14 mmol, 1.0 eq) of the free amine was dissolved in 20 mL dichloromethane and the solution was cooled to 0 °C. 0.92 mL (4.29 mmol, 2.0 eq) DIPEA was added and 0.73 mL (4.29 mmol, 2.0 eq) Boc_2O was added dropwise. After complete addition the mixture was allowed to warm to rt and stirring was continued for 16 h. Upon completion, the mixture was concentrated, and the crude product was purified by column chromatography on silica using toluene/ethyl acetate to give 1.28 g (2.01 mmol, 94%) of the respective Boc carbamate as a white solid.

^{1}H-NMR (500 MHz, $CDCl_3$), δ = 9.84 (s, 1H), 8.72-8.59 (m, 2H), 8.22-8.10 (m, 2H), 7.68 (s, 1H), 7.60-7.46 (m, 3H), 7.41 (brs, 1H), 7.34-7.27 (m, 3H), 7.20-7.11 (m, 1H), 6.69 (d, J = 8.3 Hz, 2H), 5.54 (d, J = 7.2 Hz, 1H), 5.23 (t, J = 8.1 Hz, 1H), 4.87 (d, J = 8.1 Hz, 1H), 3.56 (s, 3H), 1.61 (s, 9H), 1.44 (s, 9H) ppm. ^{13}C-NMR (125 MHz, $CDCl_3$), δ = 169.6, 158.8, 155.6, 149.8, 147.5, 137.6, 137.1, 136.0, 133.6, 131.2, 130.2, 129.8, 129.2, 128.4, 128.0, 127.6, 124.7, 123.7, 122.8, 122.1, 121.5, 119.9, 117.7, 115.3, 114.2, 83.6, 80.1, 59.5, 55.2, 45.4, 28.5, 28.3 ppm. HRMS (ESI+) Calcd for $C_{37}H_{40}N_4O_6H$ $[M+H]^+$: 637.3021, found: 637.3029.

6) Synthesis of tetrahydroquinolines

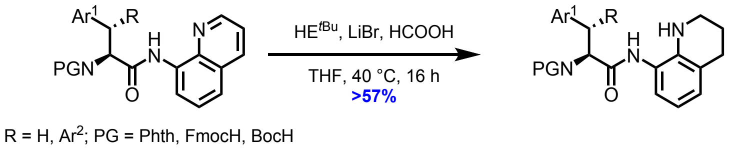

To a flame-dried pressure flask containing anhydrous LiBr (1.0 eq) was added 8-aminoquinoline amide (1.0 eq) and Hantzsch´s *tert*-butyl ester (2.6 eq). The flask was flushed with Ar. The solids were dissolved in anhydrous THF (final concentration 0.1 M). After the solids were completely dissolved, formic acid (1.0 eq) was added in a single portion and the flask was tighly sealed and heated at 40 °C for 16 h. Upon completion, volatiles were removed under reduced pressure and the residue was taken up in ethyl acetate. The organic layer was washed with 1 M aq. $NaHCO_3$ and *brine*. The organic layer was dried over $MgSO_4$, filtered and concentrated. The crude product was purified by column chromatography on silica to give the respective 8-amido tetrahydroquinolines in yields >57%.

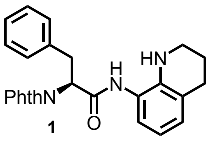

1

1.25 g, 2.94 mmol, 88% pale yellow solid. ^{1}H-NMR (500 MHz, DMSO), δ = 9.30 (s, 1H), 7.84-7.78 (m, 4H), 7.21-7.06 (m, 5H), 6.76 (t, J = 7.8 Hz, 2H), 6.42 (t, J = 7.6 Hz, 1H), 5.22 (dd, J = 11.8 Hz, 4.6 Hz, 1H), 4.88 (brs, 1H), 3.60 (dd, J = 14.0 Hz, 4.7 Hz, 1H), 3.31-3.20 (m, 3H), 2.69 (t, J = 6.2 Hz, 2H), 1.79 (quint, J = 5.7 Hz, 2H) ppm. ^{13}C-NMR (125 MHz, DMSO), δ = 167.5, 167.1, 140.4, 137.6, 134.4, 131.4, 128.8, 128.2, 127.2, 126.4, 125.2, 123.0, 121.4, 121.2, 114.4, 54.2, 41.0, 34.3, 26.8, 21.3 ppm. HRMS (ESI+) Calcd for $C_{26}H_{23}N_3O_3H$ $[M+H]^+$: 426.1812, found: 426.1809.

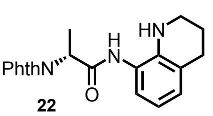

22

143 mg, 0.41 mmol, 71% pale yellow solid. ^{1}H-NMR (500 MHz, DMSO), δ = 9.18 (s, 1H), 7.93-7.89 (m, 2H), 7.88-7.83 (m, 2H), 6.75 (t, J = 6.5 Hz, 2H), 6.40 (t, J = 7.6 Hz, 1H), 4.97 (q, J = 7.2 Hz, 1H), 4.86 (brs, 1H), 3.26-3.19 (m, 2H), 2.68 (t, J = 6.3 Hz, 2H), 1.77 (quint, J = 5.9 Hz, 2H), 1.54 (d, J = 7.2 Hz, 3H) ppm. ^{13}C-NMR (125 MHz, DMSO), δ = 167.9, 167.5, 140.4, 134.4, 132.0, 127.0, 125.1, 123.0, 121.6, 121.1, 114.4, 48.1, 41.0, 26.8, 21.3, 15.2 ppm. HRMS (ESI+) Calcd for $C_{20}H_{19}N_3O_3H$ $[M+H]^+$: 350.1499, found: 350.1502.

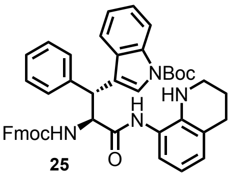

25

3.46 g, 4.72 mmol, 72%, pale yellow solid. Rotamers, ^{1}H-NMR (500 MHz, $CDCl_3$), δ = 8.11 (brs, 1H), 7.80 (s, 1H), 7.74 (d, J = 7.5 Hz, 2H) 7.50-7.30 (m, 8H), 7.30-7.16 (m, 5H), 7.11 (t, J = 7.3 Hz, 1H), 6.83 (d, J = 7.1 Hz, 1H), 6.80 (d, J = 7.5 Hz, 1H), 6.58 (t, J = 7.3 Hz, 1H), 5.54 (d, J = 6.4 Hz, 1H), 5.28-5.16 (m, 1H), 4.99 (d, J = 7.0 Hz, 1H), 4.45-4.33 (m, 1H), 4.30-4.20 (m, 1H), 4.20-4.10 (m, 1H), 3.12-2.97 (m, 2H), 2.68 (t, J = 6.3 Hz, 2H), 1.77 (quint, J = 5.8 Hz, 2H), 1.63 (s, 9H) ppm.* ^{13}C-NMR (125 MHz, $CDCl_3$), δ = 169.7, 156.4, 149.7, 143.7, 143.7, 141.4, 141.3, 138.7, 137.6, 135.6, 129.6, 129.1, 128.9, 128.4, 127.9, 127.8, 127.7, 127.2, 125.2, 125.1, 124.8, 124.3, 123.8, 123.6, 122.8, 122.6, 120.1, 120.0, 119.89, 119.85, 118.0, 115.4, 84.1, 67.6, 58.7, 47.1, 44.5, 42.0, 28.3, 27.0, 21.5 ppm. HRMS (ESI+) Calcd for $C_{46}H_{44}N_4O_5H$ $[M+H]^+$: 733.3384, found: 733.3397.

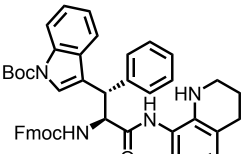

28

2.36 g, 3.22 mmol, 66%, pale yellow solid. Rotamers, ^{1}H-NMR (500 MHz, $CDCl_3$), δ = 8.10 (brs, 1H), 7.85 (s, 1H), 7.72 (t, J = 7.4 Hz, 2H), 7.54-7.41 (m, 4H), 7.41-7.27 (m, 7H), 7.25-7.09 (m, 5H), 6.80 (d, J = 7.5 Hz, 1H), 6.74 (d, J = 7.3 Hz, 1H), 6.58 (t, J = 7.4 Hz, 1H), 5.78 (d, J = 6.4 Hz, 1H), 5.10 (t, J = 8.6 Hz, 1H), 4.81 (d, J = 10.2 Hz, 1H), 4.40 (t, J = 7.6 Hz, 1H), 4.24-4.12 (m, 2H), 3.13-3.06 (m, 1H), 3.06-2.99 (m, 1H), 2.67 (t, J = 6.1 Hz, 2H), 1.82-1.74 (m, 2H), 1.57 (s, 9H) ppm.* ^{13}C-NMR (125 MHz, $CDCl_3$), δ = 169.7, 156.4, 149.7, 143.9, 143.6, 141.3, 141.2, 139.4, 137.9, 135.7, 130.1, 129.03, 128.97, 128.0, 127.9, 127.8, 127.6, 127.20, 127.18, 125.3, 125.1, 124.8, 124.1, 123.1, 122.8, 122.3, 120.1, 120.0, 119.5, 119.4, 117.8, 115.4, 84.0, 67.7, 59.6, 47.0, 45.7, 42.1, 28.2, 27.0, 21.5 ppm. HRMS (ESI+) Calcd for $C_{46}H_{44}N_4O_5H$ $[M+H]^+$: 733.3384, found: 733.3396.

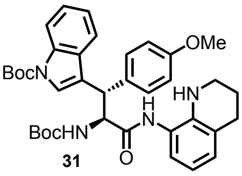

31

0.58 g, 0.90 mmol, 57%, pale yellow solid. ^{1}H-NMR (500 MHz, $CDCl_3$), δ = 8.12 (d, J = 7.2 Hz, 1H), 7.74 (s, 1H), 7.39 (d, J = 7.8 Hz, 1H), 7.34 (d, J = 8.6 Hz, 2H), 7.30-7.26 (m, 1H), 7.14 (t, J = 7.5 Hz, 1H), 6.86-6.77 (m, 4H), 6.59 (t, J = 7.4 Hz, 1H), 5.33-5.28 (m, 1H), 4.85 (t, J = 9.0 Hz, 1H), 4.68 (d, J = 10.2 Hz, 1H), 3.75 (s, 3H), 3.20-3.07 (m, 2H), 2.70 (t, J = 6.2 Hz, 2H), 1.82 (quint, J = 5.8 Hz, 2H), 1.69 (s, 9H), 1.41 (s, 9H) ppm.* ^{13}C-NMR (125 MHz, $CDCl_3$), δ = 170.0, 158.9, 155.9, 149.8, 135.8, 131.7, 130.1, 130.0, 127.9, 124.7, 124.2, 123.0, 122.8, 119.7, 119.6, 115.4, 114.3, 83.9, 80.6, 59.5, 55.4, 44.5, 42.0, 28.43, 28.35, 27.1, 21.6 ppm. HRMS (ESI+) Calcd for $C_{37}H_{44}N_4O_6H$ $[M+H]^+$: 641.3334, found: 641.3342.

* NH protons of the tetrahydroquinoline moiety are only visible in DMSO as NMR solvent due to rapid exchange with $CDCl_3$.

Disproportionation reaction of Hantzsch´s ester

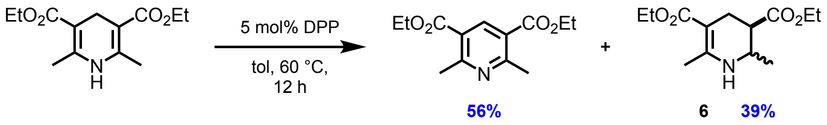

Under inert atmosphere, 400 mg (1.58 mmol) of Hantzsch´s ethyl ester and 25.8 mg (0.08 mmol) diphenyl phosphate were suspended in 10 mL toluene and heated to 60° C for 12 h. After complete conversion, volatiles were removed under reduced pressure and the crude residue was purified by column chromatography on silica using toluene/ethyl acetate to give 224 mg (0.89 mmol, 56%) of Hantzsch pyridine and 158 mg (0.62 mmol, 39%) of a 2:1 *cis*/*trans*–mixture of diastereomers (stereochemistry not assigned) of tetrahydropyridine **6** as a yellow oil.

Note: Barbe (*J. Am. Chem. Soc.* **2008**, 130, 18-19) and Miller (*Angew. Chem. Int. Ed.* **2015**, *54*, 11173-11176) used similar arrangements to generate the Hantzsch pyridine in comparable yields, however, the formation of tetrahydropyridine byproducts was not described.

6

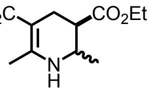

Mixture of diastereomers. ^{1}H-NMR (300 MHz, DMSO), δ = 6.86 (d, J = 3.9 Hz, NH major), 6.59 (s, NH minor), 4.19-4.03 (m, OCH_2), 4.03-3.91 (m, OCH_2), 3.69-3.58 (m, NCH major), 3.31-3.27 (m, NCH minor), 2.67-2.53 (m, CH_2), 2.35-2.15 (m, CH_2), 2.13 (s, vinyl-CH_3 minor), 2.12 (vinyl-CH_3 major), 1.22-1.12 (m, OCH_2CH_3), 1.10 (d, J = 6.4 Hz, CH_3 minor), 0.91 (d, J = 6.5 Hz, CH_3 major) ppm. ^{13}C-NMR (75 MHz, DMSO), δ = 173.5, 172.1, 167.3, 167.0, 152.9, 152.2, 88.0, 87.1, 60.0, 59.8, 57.7, 57.6, 48.0, 45.9, 44.3, 40.8, 25.9, 21.0, 20.3, 20.0, 19.2, 17.0, 14.63, 14.58, 14.0 ppm. HRMS (ESI+) Calcd for $C_{13}H_{21}NO_4Na$ $[M+Na]^+$: 278.1363, found: 278.1364.

7) Synthesis of Urea compounds (Nbz^{cyc}): general procedure

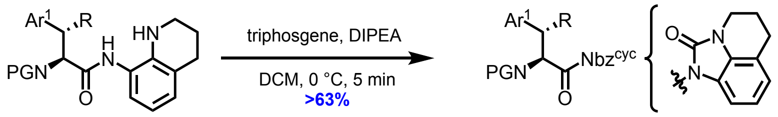

R = H, Ar^2; PG = Phth, FmocH, BocH

The tetrahydroquinoline amide (1.0 eq) was dissolved in dichloromethane (5/6 of final volume), cooled to 0 °C and DIPEA (4.0 eq) was added. Triphosgene (0.5 eq) was dissolved in dichloromethane (1/6 of final volume, final concentration 0.1 M) and added dropwise to the reaction mixture. After complete addition, stirring was continued for five minutes. Upon completion, water was added and the mixture was extracted with dichloromethane and the organic layer was washed with *brine*. The organic layer was dried over $MgSO_4$, filtered and concentrated. The crude product was purified by column chromatography on silica using toluene/ethyl acetate to give the respective acyl urea in yields >63%.

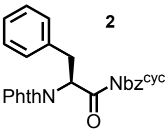

2

0.79 g, 1.77 mmol, 75%, white solid. ^{1}H-NMR (500 MHz, $CDCl_3$), δ = 7.88 (dd, J = 7.7 Hz, 0.7 Hz, 1H), 7.82-7.77 (m, 2H), 7.69-7.64 (m, 2H), 7.44 (d, J = 7.2 Hz, 2H), 7.25-7.20 (m, 2H), 7.16-7.11 (m, 1H), 7.05 (t, J = 7.7 Hz, 1H), 7.03-7.00 (m, 1H), 6.52 (dd, J = 11.3 Hz, 4.3 Hz, 1H), 3.94 (dd, J = 13.7 Hz, 11.4 Hz, 1H), 3.90 (t, J = 5.9 Hz, 2H), 3.65 (dd, J = 13.7 Hz, 4.3 Hz, 1H), 2.86 (t, J = 6.0 Hz, 2H), 2.14 (quint, J = 6.0 Hz, 2H) ppm. ^{13}C-NMR (125

MHz, $CDCl_3$), δ = 169.3, 168.3, 150.7, 137.1, 134.1, 131.9, 129.2, 128.6, 127.2, 126.9, 125.0, 123.58, 123.56, 122.6, 119.8, 114.1, 56.8, 39.4, 33.8, 23.9, 21.6 ppm. HRMS (ESI+) Calcd for $C_{27}H_{21}N_3O_4Na$ $[M+Na]^+$: 474.1435, found: 474.1425.

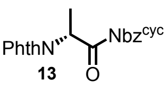

13

110 mg, 0.29 mmol, 78%, white solid. ^{1}H-NMR (500 MHz, $CDCl_3$), δ = 7.88-7.83 (m, 3H), 7.74-7.69 (m, 2H), 7.04 (t, J = 7.8 Hz, 1H), 6.99 (d, J = 7.2 Hz, 1H), 6.11 (q, J = 7.2 Hz, 1H), 3.81 (oct, J = 6.1 Hz, 2H), 2.83 (t, J = 6.7 Hz, 2H), 2.09 (quint, J = 6.0 Hz, 2H), 1.90 (d, J = 7.2 Hz, 3H) ppm. ^{13}C-NMR (125 MHz, $CDCl_3$), δ = 170.0, 167.9, 150.5, 134.1, 132.1, 127.2, 125.0, 123.6, 123.4, 122.6, 119.7, 113.9, 50.6, 39.3, 23.9, 21.6, 15.3 ppm. HRMS (ESI+) Calcd for $C_{21}H_{17}N_3O_4H$ $[M+H]^+$: 376.1292, found: 376.1295.

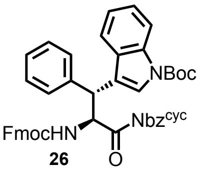

26

1.79 g, 2.36 mmol, 66%, white solid. ^{1}H-NMR (500 MHz, $CDCl_3$), δ = 8.10 (brs, 1H), 8.07 (s, 1H), 7.81 (d, J = 7.4 Hz, 1H), 7.75 (d, J = 6.2 Hz, 2H), 7.54 (d, J = 7.4 Hz, 1H), 7.50 (d, J = 8.1 Hz, 1H), 7.41-7.33 (m, 2H), 7.33-7.27 (m, 4H), 7.23-7.17 (m, 4H), 7.10-6.94 (m, 4H), 6.83-6.76 (m, 1H), 5.49 (d, J = 8.3 Hz, 1H), 4.89 (d, J = 3.0 Hz, 1H), 4.43-4.35 (m, 1H), 4.35-4.27 (m, 1H), 4.23 (t, J = 7.0 Hz, 1H), 3.81 (oct, J = 6.0 Hz, 2H), 2.85 (t, J = 5.5 Hz, 2H), 2.16-2.07 (m, 2H), 1.62 (s, 9H) ppm. ^{13}C-NMR (125 MHz, $CDCl_3$), δ = 171.6, 156.0, 150.8, 149.7, 144.0, 143.9, 141.34, 141.32, 137.5, 135.9, 129.9, 129.4, 128.8, 127.9, 127.8, 127.7, 127.2, 125.32, 125.27, 124.9, 124.3, 124.0, 123.6, 122.6, 122.3, 120.04, 120.00, 119.9, 119.80, 119.77, 115.2, 113.8, 83.5, 67.5, 56.6, 47.2, 45.3, 39.3, 28.3, 23.9, 21.5 ppm. HRMS (ESI+) Calcd for $C_{47}H_{42}N_4O_6Na$ $[M+Na]^+$: 781.2997, found: 781.3017.

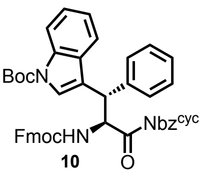

10

1.44 g, 1.90 mmol, 63%, white solid. ^{1}H-NMR (500 MHz, $CDCl_3$), δ = 8.21-8.07 (m, 1H), 7.88 (s, 1H), 7.76 (d, J = 7.7 Hz, 1H), 7.74-7.68 (m, 2H), 7.49 (d, J = 7.2 Hz, 1H), 7.41 (d, J = 7.4 Hz, 2H), 7.39-7.31 (m, 2H), 7.31-7.26 (m, 2H), 7.22 (t, J = 7.2 Hz, 1H), 7.16 (dt, J = 7.5 Hz, 0.9 Hz, 1H), 7.13-7.07 (m, 3H), 7.07-6.89 (m, 4H), 5.81-5.43 (m, 1H), 4.90 (d, J = 8.1 Hz, 1H), 4.41-4.33 (m, 1H), 4.26-4.18 (m, 2H), 3.82-3.74 (m, 1H), 3.74-3.65 (m, 1H), 2.84-2.74 (m, 2H), 2.11-2.00 (m, 2H), 1.61 (s, 9H) ppm. ^{13}C-NMR (125 MHz, $CDCl_3$), δ = 172.5, 156.0, 150.7, 149.7, 144.0, 141.34, 141.30, 138.6, 135.9, 130.2, 128.8, 128.3, 127.74, 127.68, 127.2, 127.1, 126.9, 125.31, 125.27, 124.9, 124.7, 124.1, 123.4, 122.7, 122.4, 120.0, 119.7, 119.4, 118.6, 115.4, 113.8, 83.9, 67.5, 56.9, 47.2, 46.1, 39.2, 28.3, 23.8, 21.5 ppm. HRMS (ESI+) Calcd for $C_{47}H_{42}N_4O_6H$ $[M+H]^+$: 759.3177, found: 759.3203.

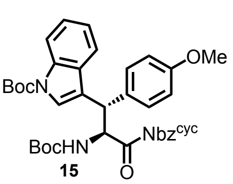

15

444 mg, 0.66 mmol, 86%, white solid. ^{1}H-NMR (500 MHz, $CDCl_3$), δ = 8.18-8.09 (m, 1H), 7.86 (s, 1H), 7.74 (d, J = 7.7 Hz, 1H), 7.26-7.16 (m, 4H), 7.05 (t, J = 7.5 Hz, 1H), 6.98 (t, J = 7.8 Hz, 1H), 6.94 (d, J = 7.5 Hz, 1H), 6.89-6.63 (m, 1H), 6.52 (d, J = 8.6 Hz, 2H), 5.48-5.03 (m, 1H), 4.63 (d, J = 8.8 Hz, 1H), 3.77-3.69 (m, 1H), 3.69-3.59 (m, 1H), 3.54 (s, 3H), 2.83-2.71 (m, 2H), 2.07-2.00 (m, 2H), 1.69 (s, 9H), 1.38 (s, 9H) ppm. ^{13}C-NMR (125 MHz, $CDCl_3$), δ = 173.4, 158.6, 155.3, 150.7, 149.8, 135.9, 130.6, 130.3, 129.9, 126.8, 125.1, 124.5, 123.7, 123.2, 122.5, 122.2, 119.6, 119.3, 119.1, 115.3, 113.6, 113.4, 83.8, 80.0, 56.2, 55.1, 46.1, 39.1, 28.4, 28.4, 23.9, 21.5 ppm. HRMS (ESI+) Calcd for $C_{38}H_{42}N_4O_7Na$ $[M+Na]^+$: 689.2946, found: 689.2971.

8) Nbz^{cyc} cleavage reactions

Hydrolysis reactions

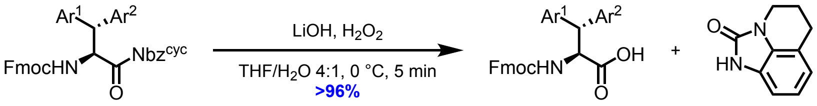

The acyl urea compound (1.0 eq) was dissolved in THF (4/5 of final volume) and cooled to 0 °C. Lithium hydroxide monohydrate (1.1 eq) was dissolved in water (1/5 of final volume, final concentration 0.1 M) and 50% aq. hydrogen peroxide (8.8 eq) was added. The aqueous solution of *in situ* generated lithium hydroperoxide was added dropwise to the solution containing the acyl urea and the mixture was stirred at 0 °C for five minutes. Upon completion, 1 M aq. sodium sulfite (12 eq) was added dropwise, ensuring that the temperature does not exceed 10 °C. After complete addition, the mixture was acidified to pH = 2 using 6 M aq. HCl by dropwise addition. The ice bath was removed and the mixture was extracted two times with ethyl acetate. The combined organic layer was washed with *brine*, dried over $MgSO_4$, filtered and concentrated. The crude product was purified by column chromatography on silica using cyclohexane/ethyl acetate/acetic acid to give the free carboxylic acids in high yields (>96%).

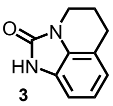

3

White solid. ^{1}H-NMR (300 MHz, DMSO), δ = 10.58 (s, 1H), 6.85 (dd, J = 8.8 Hz, 6.2 Hz, 1H), 6.80-6.72 (m, 2H), 3.69 (t, J = 5.7 Hz, 2H), 2.76 (t, J = 6.0 Hz, 2H), 1.99 (quint, J = 5.9 Hz, 2H) ppm. ^{13}C-NMR (75 MHz, DMSO), δ = 153.5, 127.5, 126.6, 120.2, 118.9, 118.5, 106.2, 38.1, 23.2, 21.6 ppm. HRMS (ESI+) Calcd for $C_{10}H_{310}N_2OH$ $[M+H]^+$: 175.0866, found: 175.0866.

12

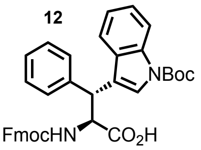

1.23 g, 2.04 mmol, 97%, white solid. Rotamers, ^{1}H-NMR (500 MHz, DMSO), δ = 12.71 (s, 1H), 8.00 (d, J = 8.3 Hz, 1H), 7.95-7.82 (m, 3H), 7.59-7.55 (m, 2H), 7.46-7.38 (m, 5H), 7.31-7.26 (m, 3H), 7.26-7.21 (m, 2H), 7.20-7.16 (m, 1H), 7.16-7.11 (m, 1H), 4.93 (dd, J = 10.4 Hz, 9.5 Hz, 1H), 4.66 (d, J = 10.6 Hz, 1H), 4.20 (dd, J = 13.9 Hz, 10.8 Hz, 1H), 4.11-4.05 (m, 2H), 1.63 (s, 9H) ppm. ^{13}C-NMR (125 MHz, DMSO), δ = 172.5, 155.7, 149.0, 143.7, 143.6, 140.65, 140.60, 139.9, 134.5, 129.5, 128.7, 128.2, 127.63, 127.61, 127.08, 127.00, 126.7, 125.4, 125.2, 124.5, 122.62, 122.57, 120.6, 120.1, 120.0, 119.4, 114.7, 83.8, 65.9, 57.4, 46.5, 43.2, 27.7 ppm. HRMS (ESI+) Calcd for $C_{37}H_{34}N_2O_6Na$ $[M+Na]^+$: 625.2309, found: 625.2326.

11

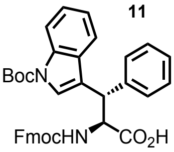

1.10 g, 1.83 mmol, 96%, white solid. Rotamers, ^{1}H-NMR (500 MHz, DMSO), δ = 12.58 (s, 1H), 8.17 (d, J = 9.0 Hz, 1H), 7.97 (d, J = 8.0 Hz, 1H), 7.85 (t, J = 7.5 Hz, 2H), 7.76 (s, 1H), 7.57 (d, J = 7.5 Hz, 1H), 7.45 (d, J = 7.9 Hz, 1H), 7.42-7.32 (m, 5H), 7.30-7.23 (m, 3H), 7.21-7.13 (m, 3H), 7.03 (t, J = 7.4 Hz, 1H), 4.89 (dd, J = 10.8 Hz, 9.1 Hz, 1H), 4.67 (d, J = 10.8 Hz, 1H), 4.42 (dd, J = 10.5 Hz, 7.1 Hz, 1H), 4.23 (t, J = 7.6 Hz, 1H), 3.98 (dd, J = 10.4 Hz, 8.4 Hz, 1H), 1.44 (s, 9H) ppm. ^{13}C-NMR (125 MHz, DMSO), δ = 172.1, 156.1, 148.9, 143.8, 143.4, 140.7, 140.6, 139.7, 134.8, 129.9, 128.7, 128.3, 127.7, 127.5, 126.99, 126.95, 126.8, 125.3, 125.0, 124.4, 122.4, 122.0, 120.4, 120.1, 120.0, 119.5, 114.7, 83.4, 66.1, 57.7, 46.4, 44.3, 27.45 ppm. HRMS (ESI+) Calcd for $C_{37}H_{34}N_2O_6H$ $[M+H]^+$: 603.2490, found: 603.2512.

Esterification

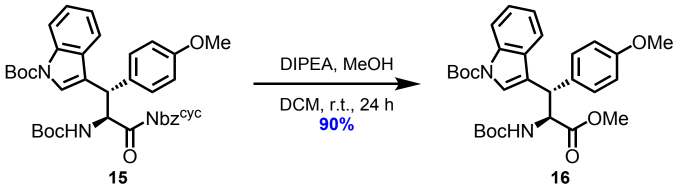

33.3 mg (0.05 mmol, 1.0 eq) Boc-Wsy(Boc,Me)-Nbz^{cyc} (**15**) was dissolved in 0.5 mL dichloromethane/methanol (1:1) and 44 µL (0.25 mmol, 5.0 eq) DIPEA was added. The mixture was stirred at r.t. for 24 h. Upon completion, volatiles were removed under reduced pressure. The crude product was purified by column chromatography on silica using toluene/ethyl acetate to give 23.5 mg (45 µmol, 90%) of Boc-Wsy(Boc,Me)-OMe (**16**) as a white solid.

^{1}H-NMR (500 MHz, $CDCl_3$), δ = 8.11 (d, J = 7.5 Hz, 1H), 7.71 (s, 1H), 7.28-7.24 (m, 2H), 7.20 (d, J = 8.6 Hz, 2H), 7.13-7.09 (m, 1H), 6.81-6.78 (m, 2H), 5.10 (d, J = 8.5 Hz, 1H), 4.99 (t, J = 8.5 Hz, 1H), 4.52 (d, J = 8.5 Hz, 1H), 3.75 (s, 3H), 3.48 (s, 3H), 1.68 (s, 9H), 1.40 (s, 9H) ppm. ^{13}C-NMR (125 MHz, $CDCl_3$), δ = 172.5, 158.9, 155.3, 149.8, 135.7, 131.1, 130.2, 129.6, 124.6, 123.2, 122.7, 119.6, 119.4, 115.3, 114.0, 83.9, 80.2, 57.6, 55.3, 52.2, 45.4, 28.4 ppm. HRMS (ESI+) Calcd for $C_{29}H_{36}N_2O_7Na$ $[M+Na]^+$: 547.2415, found: 547.2423.

Dipeptide coupling

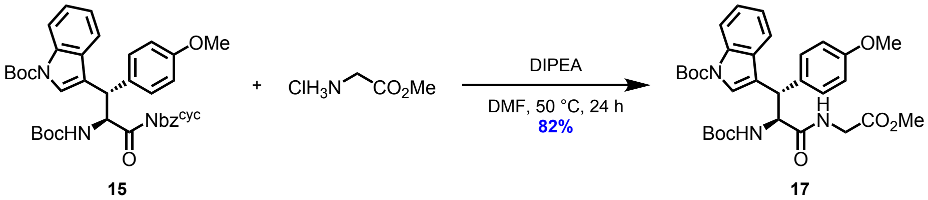

33.3 mg (0.05 mmol, 1.0 eq) Boc-Wsy(Boc,Me)-Nbz^{cyc} (**15**) and 12.5 mg (0.10 mmol, 2.0 eq) glycine methylester hydrochloride were dissolved in 0.5 mL DMF and 17 µL (0.10 mmol, 2.0 eq) DIPEA was added. The solution was stirred at 50 °C for 24 h. Upon completion, the mixture was diluted with EtOAc. The organic layer was washed with 1 M aq. HCl, twice with 5% aq. LiCl solution and *brine*. The organic layer was dried over $MgSO_4$, filtered and concentrated. The crude product was purified by column chromatography on silica to give 23.9 mg (41 µmol, 82%) of dipeptide Boc-Wsy(Boc,Me)-Gly-OMe (**17**) as a white solid.

^{1}H-NMR (500 MHz, $CDCl_3$), δ = 8.20-8.02 (m, 1H), 7.69 (s, 1H), 7.34 (d, J = 7.8 Hz, 1H), 7.28-7.25 (m, 1H), 7.35-7.21 (m, 2H), 7.14-7.10 (m, 1H), 6.78 (d, J = 8.9 Hz, 2H), 6.12 (s, 1H), 5.22 (d, J = 7.9 Hz, 1H), 4.82 (t, J = 8.9 Hz, 1H), 4.66 (d, J = 8.5 Hz, 1H), 3.90 (dd, J = 18.3 Hz, 5.0 Hz, 1H), 3.76-3.68 (m, 4H), 3.66 (s, 3H), 1.67 (s, 9H), 1.39 (s, 9H) ppm. ^{13}C-NMR (125 MHz, $CDCl_3$), δ = 171.1, 169.5, 158.8, 155.6, 149.8, 135.7, 131.6, 130.1, 129.5, 124.6, 123.1, 122.7, 119.63, 119.58, 115.3, 114.1, 83.7, 80.4, 58.3, 55.3, 52.4, 44.6, 41.4, 28.4, 28.3 ppm. HRMS (ESI+) Calcd for $C_{31}H_{39}N_3O_8Na$ $[M+Na]^+$: 604.2629, found: 604.2615.

$NaBH_4$ reduction

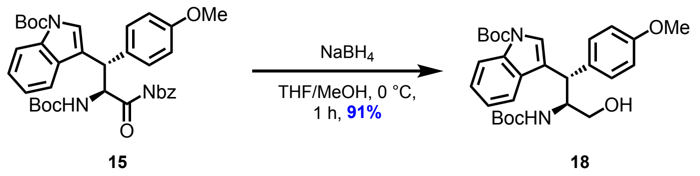

3.8 mg (0.1 mmol, 2.0 eq) sodium borohydride was dissolved in 0.5 mL THF/MeOH (1:1) and cooled to 0 °C. 33.3 mg (0.05 mmol, 1.0 eq) Boc-Wsy(Boc,Me)-Nbz^{cyc} (**15**) was added portionwise and stirred for 1 h. After completion, sat. NH_4Cl was added and the mixture was extracted with ethyl acetate. The organic layer was washed with *brine*, dried over $MgSO_4$, filtered and concentrated. The crude residue was purified by column chromatography on silica using toluene/ethyl acetate to furnish 22.6 mg (45 μmol, 91%) of the alcohol **18** as a white solid.

^{1}H-NMR (500 MHz, DMSO), δ = 8.00 (d, J = 8.2 Hz, 1H), 7.76 (s, 1H), 7.41 (d, J = 7.8 Hz, 1H), 7.29.7.21 (m, 3H), 7.12 (t, J = 7.5 Hz, 1H), 6.82 (d, J = 8.7 Hz, 2H), 6.70 (d, J = 9.5 Hz, 1H), 4.53 (t, J = 5.4 Hz, 1H), 4.25 (d, J = 11.2 Hz, 1H), 4.19-4.11 (m, 1H), 3.68 (s, 3H), 3.29-3.24 (m, 1H), 3.17-3.10 (m, 1H), 1.61 (s, 9H), 1.33 (s, 9H) ppm. ^{13}C-NMR (125 MHz, DMSO), δ = 157.7, 155.6, 149.1, 134.8, 133.6, 130.3, 129.2, 124.1, 122.32, 122.29, 121.4, 119.5, 114.6, 113.8, 83.3, 77.5, 61.9, 54.93, 54.86, 42.9, 28.2, 27.7 ppm. HRMS (ESI+) Calcd for $C_{28}H_{36}N_2O_6Na$ $[M+Na]^+$: 519.2477, found: 519.2487.

9) Peptide synthesis

Leu-enkephalin derivative

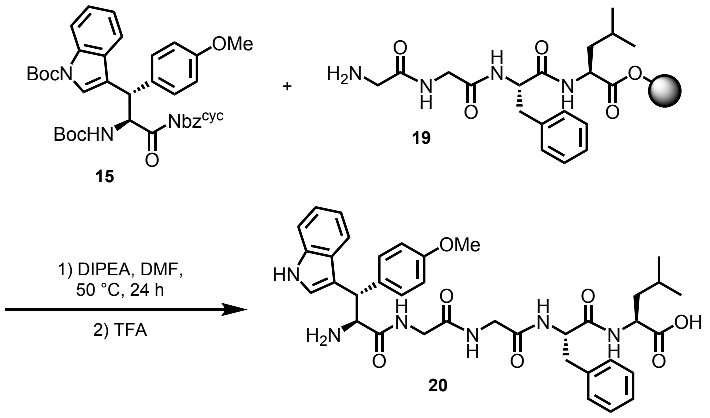

The Leu-enkephalin derivative was synthesized in 0.1 mmol scale. Preloaded 2-Chlorotrityl-resin with C-terminal amino acid (loading approx. 0.6 mmol/g) was suspended in DMF and shaken for 30 min. The slurry was transferred to the reaction chamber of the peptide synthesizer and the excess DMF was removed. DMF was used as solvent for washing, coupling and Fmoc removal steps. Fmoc-removal was achieved using 20 vol% piperidine in DMF for 7 minutes at rt. Microwave assisted single coupling of the Fmoc-protected amino acids was achieved using 5 eq of Fmoc-Xaa-OH, DIC and Oxyma for 5 minutes at 50 °C. After complete synthesis of the resin-bound peptide 19, the resin was portioned in half. 0.5 mmol of the resin were treated with TFA for 1 h, filtered and volatiles were removed under reduced pressure to give a crude tetrapeptide, which was dissolved in water and freeze-dried. The crude peptide was analyzed by *rp*-HPLC to serve as an authentic sample for reaction monitoring. The other half (0.5 mmol) of the resin were suspended in 375 μL DMF and

50.0 mg (75 µmol, 1.5 eq) Boc-Wsy(Boc,Me)-Nbz^{cyc} (**15**) and 17 µL (2 eq, 0.1 mmol) DIPEA were added and rotated at 50 °C using a RotaVap. The progress was monitored using *rp*-HPLC. After completion, the residue was transferred to a frit using DCM. The resin was washed three times with DMF and three times with DCM. The resin was dried under reduced pressure. Peptide cleavage was achieved by treatment of the resin with TFA and filtration. Volatiles were removed under reduced pressure and the crude peptide was purified *via* *rp*-HPLC using H_2O with 0.085% TFA and MeCN with 0.085% TFA as the solvent system. Product containing fractions were combined and freeze-dried to give the target peptide **20** in >98% purity.

HPLC-MS (ESI+) Calcd for $C_{37}H_{44}N_6O_7H$ $[M+H]^+$: 685.3344, found: 685.3339. *rp*-HPLC (0.45 mL/min, 10-50% B in 10 min, 28 °C, UV = 215 nm): 7.50 min.

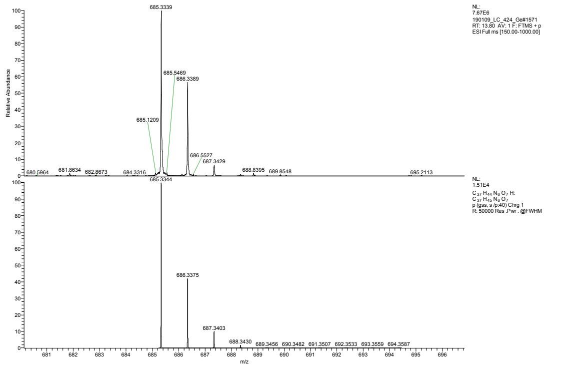

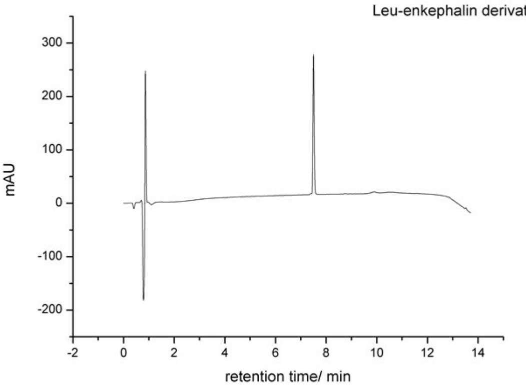

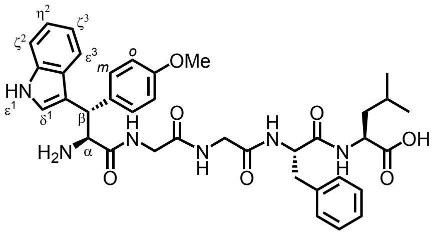

H-Wsy¹-Gly²-Gly³-Phe⁴-Leu⁵-OH

Table: TOCSY-NMR data of peptide **20** in DMSO (600 MHz, 300 K). Chemical shifts are described in ppm.

residue	NH	α	β	others
Wsy[1]	exchange	4.54	4.54	*o* = 6.75; *m* = 7.24; OMe = 3.64; δ^1 = 7.43; ε^1 = 11.03; ε^3 = 7.47; ζ^2 = 7.34; ζ^3 = 6.93; η^2 = 7.05
Gly[2]	8.42	3.68; 3.36	-	-
Gly[3]	7.76	3.66; 3.56	-	-
Phe[4]	8.04	4.54	3.05; 2.76	*p* = 7.18; *o*, *m* = 7.24
Leu[5]	8.23	4.20	1.54	γ = 1.63; δ = 0.89, 0.84

Trp cage mutants

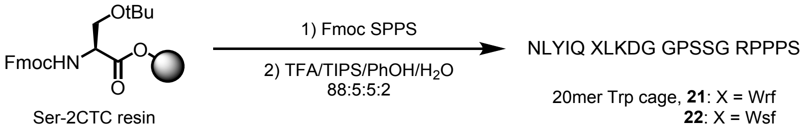

The Trp cage mutants were synthesized in 0.1 mmol scale. Preloaded 2-Chlorotrityl-resin with C-terminal amino acid (loading approx. 0.6 mmol/g) was suspended in DMF and shaken for 30 min. The slurry was transferred to the reaction chamber of the peptide synthesizer and the excess DMF was removed. DMF was used as solvent for washing, coupling and Fmoc removal steps. Fmoc-removal was achieved using 20 vol% piperidine in DMF for 7 minutes at rt. Microwave assisted single coupling of the Fmoc-protected amino acids was achieved using 5 eq of Fmoc-Xaa-OH, DIC and Oxyma for 5 minutes at 50 °C. The coupling of the unnatural building blocks Fmoc-Wrf(Boc)-OH and Fmoc-Wsf(Boc)-OH were done using 2 eq of the amino acid for 30 minutes at 50 °C (Arg-single coupling CTC). After complete synthesis, the resin was transferred into a frit and washed several times with DCM and dried under high vacuum. The peptide was cleaved from the resin *via* addition of 5 mL of the cleavage cocktail containing TFA/TIPS/PhOH/H_2O (88:5:5:2, vol/vol/wt/vol) and agitation for 3 h. The resin was filtered and the solution was slowly added to 30 mL of chilled diethyl ether to precipitate the peptide and centrifuged. The supernatant was discarded and the precipitate was suspended in diethyl ether and centrifuged two more times, after which the crude peptide was dried under high vacuum. The residue was dissolved in 10% MeCN in H_2O and freeze-dried to give 120 mg of the peptide as its TFA-salt. 35 mg of the crude peptide were purified *via rp*-HPLC using H_2O with 0.085% TFA and MeCN with 0.085% TFA as the solvent system. Product containting fractions were combined and freeze-dried to give the target peptide **21** and **22** in >98% purity.

21: HPLC-MS (ESI+) Calcd for $C_{104}H_{153}N_{27}O_{29}H_2$ $[M+2H]^{2+}$: 1123.0737, found: 1123.0777. *rp*-HPLC (0.45 mL/min, 10-50% B in 10 min, 28 °C, UV = 215 nm): 8.17 min.

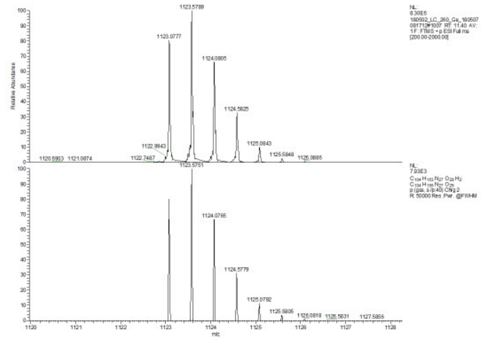

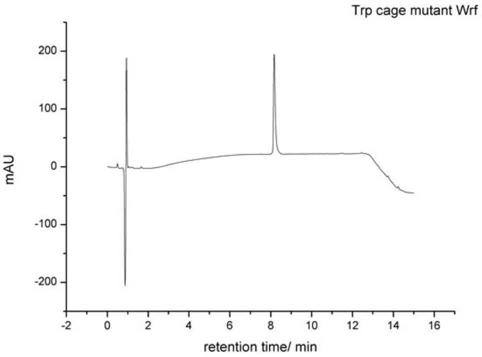

Table: TOCSY-NMR data with partial assignment of peptide **21** in 50 mM phosphate buffer pH 7.4, TFE-d_2, D_2O 60%/30%/10% (600 MHz, 280 K). Chemical shifts are described in ppm. n/a = not assigned

residue	NH	α	β	others
Asn[1]	exchange	3.74	2.76	NH_2 = 7.65, 6.56
Leu[2]	8.36	3.90	n/a	n/a
Tyr[3]	7.92	4.29	3.19, 3.12	*m* = 7.17; *o* = 6.88
Ile[4]	7.58	3.80	1.98	γ^1, γ^2, δ^2 = 0.95
Gln[5]	7.59	3.86	2.06	γ = 2.32; NH_2 = 7.78, 6.76
Wrf[6]	7.86	5.07	4.99	*o* = 7.33; *m* = 7.27; *p* = 7.19; δ^1 = 6.98; ε^1 = 10.32; ε^3 = 7.61; ζ^2 = 7.17; ζ^3 = 7.36; η^2 = 7.12
Leu[7]	8.43	3.50	2.03, 1.85	γ = 1.51; δ = 0.95, 0.86
Lys[8]	8.81	3.94	1.93	γ = 1.48; δ = 1.64; ε = 2.94
Asp[9]	7.96	4.77	2.94, 2.85	-
Gly[10]	7.82	4.13, 3.65	-	-
Gly[11]	8.11	2.29	-	-
Pro[12]	-	4.40	n/a	n/a
Ser[13]	7.84	4.42	3.98, 3.91	-
Ser[14]	8.11	4.31	4.03, 3.96	-
Gly[15]	8.03	4.23, 3.81	-	-
Arg[16]	8.01	4.85	1.74, 1.66	γ = 1.93; δ = 3.21
Pro[17]	-	4.61	2.22, 1.81	γ = 1.93; δ = 3.76, 3.59
Pro[18]	-	3.49	1.63, 1.37	γ = 1.91; δ = 3.29, 3.21
Pro[19]	-	4.24	2.09, 1.87	γ = 1.67; δ = 2.92, 2.41
Ser[20]	7.59	4.20	3.83	-

22: HPLC-MS (ESI+) Calcd for $C_{104}H_{153}N_{27}O_{29}H_2$ $[M+2H]^{2+}$: 1123.0737, found: 1123.0769. *rp*-HPLC (0.45 mL/min, 10-40% B in 10 min, 28 °C, UV = 215 nm): 9.69 min.

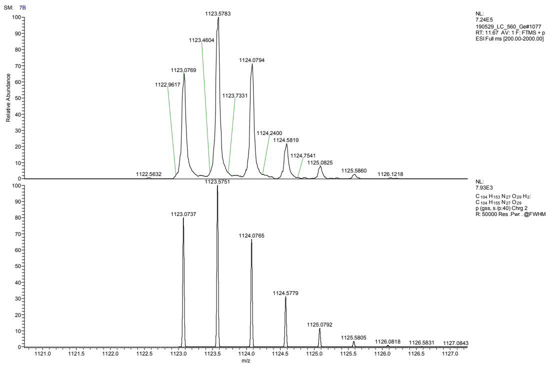

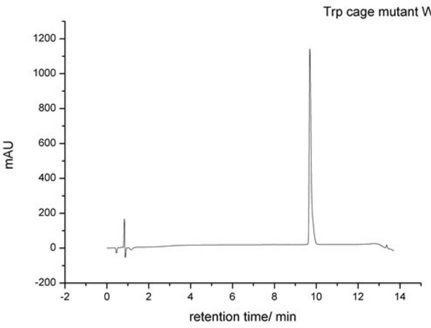

Table: TOCSY-NMR data with partial assignment of peptide **22** in 50 mM phosphate buffer pH 7.4, TFE-d_2, D_2O 60%/30%/10% (600 MHz, 280 K). Chemical shifts are described in ppm. n/a = not assigned

residue	NH	α	β	others
Asn[1]	exchange	3.65	2.68	NH_2 = 7.58, 6.50
Leu[2]	8.06	n/a	n/a	n/a
Tyr[3]	7.30	4.10	2.96	*m* = 7.11; *o* = 6.90
Ile[4]	7.46	3.74	1.92	γ^1, γ^2, δ^2 = 0.90
Gln[5]	7.55	3.89	2.19	γ = 2.48; NH_2 = 7.82, 6.78
Wsf[6]	8.29	5.07	4.87	*o* = 7.25; *m* = 7.20; *p* = 7.25; δ^1 = 7.44; ε^1 = 9.85; ε^3 = 7.36; ζ^2 = 6.88; ζ^3 = 7.08; η^2 = 7.15
Leu[7]	8.43	3.70	2.05, 1.42	γ = 1.07; δ = 0.82, 0.73
Lys[8]	8.66	4.00	1.95	γ = 1.47; δ = 1.64; ε = 2.96
Asp[9]	8.23	4.74	2.88	-
Gly[10]	8.02	4.08, 3.78	-	-
Gly[11]	8.17	3.59, 3.27	-	-
Pro[12]	-	4.53	2.36, 2.02	γ = 2.10; δ = 3.70, 3.42
Ser[13]	8.22	4.46	4.00, 3.91	-
Ser[14]	8.15	4.40	3.99, 3.95	-
Gly[15]	8.28	4.06, 3.91	-	-
Arg[16]	7.93	4.70	1.85	γ = 1.71, 1.65; δ = 3.17
Pro[17]	-	4.63	2.22, 1.87	γ = 1.99, 1.94; δ = 3.76, 3.58
Pro[18]	-	4.41	1.79, 1.75	γ = 1.93; δ = 3.64, 3.50
Pro[19]	-	4.38	2.20, 1.95	γ = 1.87; δ = 3.53, 3.20
Ser[20]	7.77	4.26	3.88	-

CSD – Values Trp Cage and mutants thereof

Random coil shifts reported by Sykes *et al*. were used as reference (D. S. Wishart, C. G. Bigam, A. Holm, R. S. Hodges and B. D. Sykes, J. Biomol. NMR 1995, 5, 67-81). Values in bold show amino acids followed by proline in the sequence.

Table: Random coil shifts in ppm.

residue	L Hα	P Hα	P Hβ	P Hδ	G Hα
	4.34	4.73	**1.91**	3.63	**4.13**

Table: Chemical shifts in ppm.

peptide	L^7 Hα	P^{18} Hα	P^{18} Hβ_3	P^{19} Hδ_3	G^{11} Hα_2
Trp^6	3.50	2.49	0.18	3.12	0.65
Wrf^6	3.52	3.49	1.37	2.92	2.30
Wsf^6	3.72	4.41	1.75	3.53	3.29

Table: CSD-Values of Trp cage and mutants thereof (CSD: $\Delta\delta = \delta_{observed} - \delta_{rc}$).

peptide	L^7 Hα	P^{18} Hα	P^{18} Hβ_3	P^{19} Hδ_3	G^{11} Hα_2
Trp^6	-0.84	-2.24	-1.73	-0.51	-3.48
Wrf^6	-0.82	-1.24	-0.54	-0.71	-1.83
Wsf^6	-0.62	-0.32	-0.16	-0.10	-0.84

Table: Folding fraction, Trp cage TC5b as 100%-folded reference.

peptide	L^7 Hα	P^{18} Hα	P^{18} Hβ_3	P^{19} Hδ_3	G^{11} Hα_2	Average
Wrf^6	98%	55%	31 %	139%	53%	**75%**
Wsf^6	74%	14%	9%	20%	24%	**28%**

10) X-ray analysis

Crystal structure of *rac*-4

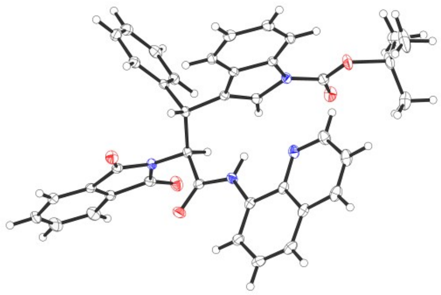

Dr. Klaus Harms
Chemistry Department
University of Marburg

CCDC 1903900

Table 1. Crystal data and structure refinement for LN307_0m.

Crystal data

Identification code	LN307_0m	
Habitus, colour	nugget, colourless	
Crystal size	0.33 x 0.16 x 0.16 mm^3	
Crystal system	Triclinic	
Space group	P-1	Z = 2
Unit cell dimensions	a = 10.5439(5) Å	α = 87.794(2)°.
	b = 12.3994(5) Å	β = 79.277(2)°.
	c = 14.5971(6) Å	γ = 68.813(2)°.
Volume	1747.47(13) $Å^3$	
Cell determination	9945 peaks with Theta 2.3 to 27.5°.	
Empirical formula	$C_{41} H_{40} N_4 O_7$	
Moiety formula	$C_{39} H_{32} N_4 O_5$, 2($C H_4 O$)	
Formula weight	700.77	
Density (calculated)	1.332 Mg/m^3	
Absorption coefficient	0.092 mm^{-1}	
F(000)	740	

Data collection:

Diffractometer type	Bruker D8 QUEST area detector
Wavelength	0.71073 Å
Temperature	100(2) K

Theta range for data collection	2.298 to 27.541°.
Index ranges	-13<=h<=13, -16<=k<=16, -18<=l<=18
Data collection software	APEX3 (Bruker AXS Inc., 2015)[1]
Cell refinement software	SAINT V8.35A (Bruker AXS Inc., 2015)[2]
Data reduction software	SAINT V8.35A (Bruker AXS Inc., 2015)

Solution and refinement:

Reflections collected	72214
Independent reflections	8041 [R(int) = 0.0359]
Completeness to theta = 25.242°	99.9 %
Observed reflections	6839[I > 2σ(I)]
Reflections used for refinement	8041
Extinction coefficient	X = 0.0077(8)
Absorption correction	Semi-empirical from equivalents[3]
Max. and min. transmission	0.99 and 0.95
Largest diff. peak and hole	0.558 and -0.429 e.Å^{-3}
Solution	dual space algorithm[4]
Refinement	Full-matrix least-squares on F^2
Treatment of hydrogen atoms	CH riding model, OH, NH located, isotr. ref.
Programs used	XT V2014/1 (Bruker AXS Inc., 2014)[4]
	SHELXL-2017/1 (Sheldrick, 2017)[5]
	DIAMOND (Crystal Impact)[6]
	ShelXle (Hübschle, Sheldrick, Dittrich, 2011)[7]
Data / restraints / parameters	8041 / 0 / 487
Goodness-of-fit on F^2	1.051
R index (all data)	wR2 = 0.1093
R index conventional [I>2sigma(I)]	R1 = 0.0458

[1] *APEX3*, Bruker AXS Inc., Madison, Wisconsin, USA, **2016**.

[2] *SAINT*, Bruker AXS Inc., Madison, Wisconsin, USA, **2015**.

[3] *SADABS. Bruker AXS area detector scaling and absorption correction*, Bruker AXS Inc., Madison, Wisconsin, USA, **2016**.

[4] G. M. Sheldrick, *Acta Crystallogr A Found Adv* **2015**, *71*, 3.

[5] G. M. Sheldrick, *Acta crystallographica. Section C, Structural chemistry* **2015**, *71*, 3.

[6] K. Brandenburg, *Diamond - Crystal and Molecular Structure Visualization*, Crystal Impact - Dr. H. Putz & Dr. K. Brandenburg GbR, Bonn, Germany, **2014**.

[7] C. B. Hübschle, G. M. Sheldrick, B. Dittrich, *Journal of applied crystallography* **2011**, *44*, 1281.

Crystal structure of 13

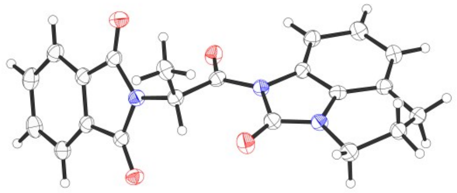

Dr. Klaus Harms
Chemistry Department
University of Marburg

Single-crystal X-ray analysis of LN513.
Data was collected with an STOE STADIVARI diffractometer eqipped with with CuKα radiation, a graded multilayer mirror monochromator (λ = 1.54186 Å) and a DECTRIS PILATUS 300K detector using an oil-coated schock-cooled crystal at 100(2) K. Absorption effects were corrected semi-empirical using multiscanned reflexions (X-Area LANA 1.68.2.0 (STOE, 2016)). Cell constants were refined using 18991 of observed reflections of the data collection. The structure was solved by direct methods by using the program XT V2014/1 (Bruker AXS Inc., 2014) and refined by full matrix least squares procedures on F^2 using SHELXL-2017/1 (Sheldrick, 2017). The non-hydrogen atoms have been refined anisotropically, carbon bonded hydrogen atoms were included at calculated positions and refined using the 'riding model' with isotropic temperature factors at 1.2 times (for CH_3 groups 1.5 times) that of the preceding carbon atom. CH_3 groups were allowed to rotate about the bond to their next atom to fit the electron density The Flack parameter refined to 0.09(9). The absolute configuration could be determined.

CCDC 1903901

Table 1. Crystal data and structure refinement for LN513.

Crystal data

Identification code	LN513	
Habitus, colour	plate, colorless	
Crystal size	0.69 x 0.33 x 0.07 mm^3	
Crystal system	Monoclinic	
Space group	$P2_1$	Z = 2
Unit cell dimensions	a = 8.5853(4) Å	α= 90°.
	b = 7.0808(2) Å	β= 96.828(4)°.
	c = 14.4608(7) Å	γ = 90°.
Volume	872.85(6) $Å^3$	
Cell determination	18991 peaks with Theta 5.2 to 71.5°.	
Empirical formula	$C_{21} H_{17} N_3 O_4$	
Moiety formula	$C_{21} H_{17} N_3 O_4$	
Formula weight	375.38	

Density (calculated)	1.428 Mg/m^3
Absorption coefficient	0.833 mm^{-1}
F(000)	392

Data collection:

Diffractometer type	STOE STADIVARI
Wavelength	1.54186 Å
Temperature	100(2) K
Theta range for data collection	5.189 to 71.573°.
Index ranges	-4<=h<=10, -8<=k<=8, -17<=l<=17
Data collection software	X-Area Pilatus3_SV 1.31.127.0 (STOE, 2016)[1]
Cell refinement software	X-Area Recipe 1.33.0.0 (STOE, 2015)[2]
Data reduction software	X-Area Integrate 1.71.0.0 (STOE, 2016)[3]
	X-Area LANA 1.68.2.0 (STOE, 2016)[4]

Solution and refinement:

Reflections collected	14642
Independent reflections	3282 [R(int) = 0.0295]
Completeness to theta = 67.686°	99.4 %
Observed reflections	3030[I > 2σ(I)]
Reflections used for refinement	3282
Absorption correction	Semi-empirical from equivalents[5]
Max. and min. transmission	0.7847 and 0.1720
Flack parameter (absolute struct.)	0.09(9)
Largest diff. peak and hole	0.176 and -0.192 e.Å^{-3}
Solution	dual space algorithm
Refinement	Full-matrix least-squares on F^2
Treatment of hydrogen atoms	calculated positions, constr ref.
Programs used	XT V2014/1 (Bruker AXS Inc., 2014)[6]
	SHELXL-2017/1 (Sheldrick, 2017)[7]
	DIAMOND (Crystal Impact)[8]
	ShelXle (Hübschle, Sheldrick, Dittrich, 2011)[9]
Data / restraints / parameters	3282 / 19 / 264
Goodness-of-fit on F^2	1.080
R index (all data)	wR2 = 0.0844
R index conventional [I>2sigma(I)]	R1 = 0.0312

[1] *X-Area Pilatus3_SV*, STOE & Cie GmbH, Darmstadt, Germany, **2016**.
[2] *X-Area Recipe*, STOE & Cie GmbH, Darmstadt, Germany, **2015**.
[3] *X-Area Integrate*, STOE & Cie GmbH, Darmstadt, Germany, **2016**.
[4] *X-Area LANA*, STOE & Cie GmbH, Darmstadt, Germany, **2016**.
[5] *SADABS. Bruker AXS area detector scaling and absorption correction*, Bruker AXS Inc., Madison, Wisconsin, USA, **2016**.
[6] G. M. Sheldrick, *Acta Crystallogr A Found Adv* **2015**, *71*, 3.
[7] G. M. Sheldrick, *Acta crystallographica. Section C, Structural chemistry* **2015**, *71*, 3.
[8] K. Brandenburg, *Diamond - Crystal and Molecular Structure Visualization*, Crystal Impact - Dr. H. Putz & Dr. K. Brandenburg GbR, Bonn, Germany, **2014**.
[9] C. B. Hübschle, G. M. Sheldrick, B. Dittrich, *Journal of applied crystallography* **2011**, *44*, 1281.

11) Computational methods

The calculations were performed using Gaussian 09 version C.01 suite of programs.[1] The geometry optimization for the urea compound started from the crystal structure of compound **13**. For the aminoquinoline amide, the x-ray structure of compound *rac*-**4** was used, which was then modified using *pymol*[2] to get the initial structure in analogue to compound **13**. We used B3LYP/6-311++G(d,p)[3,4] level of theory for the optimization in the gas phase. Extensive studies by various authors have showed that this level is accurate enough for the prediction of energetics of amides. Furthermore, the optimized structure showed excellent agreement to the x-ray structure.

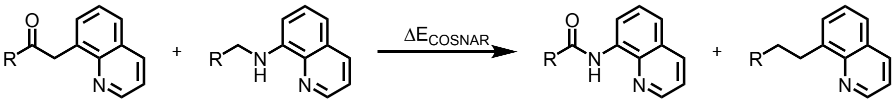

For the calculation of the resonance energies we employed the COSNAR method[5] following the depiction. The ketone, amine and hydrocarbon model compounds were built using *pymol* to alter the parent amide structure. This initial conformation was used to generate multiple structures through Frog2.[6] The lowest 20 energy conformers were then subjected to minimization in Gaussian 09. The absence of imaginary frequencies was used to characterize the structures as minima on the PES. ZPE and thermal energies were calculated under standard conditions (1atm and 298.15 K).

To compare compound **13** to recently developed amides for organic synthesis, we employed *N*-acetylglutarimide developed by Szostak *et al.*[7] to the COSNAR method. The following table shows the collation of WINKLER-DUNITZ values.

parameter	*N*-acetylglutarimide	amide *rac*-**4**	urea **13**
τ [°]	90.0	3.0	8.4
χ_N [°]	1.0	0.3	6.3
χ_C [°]	0.0	3.4	0.9
$\tau + \chi_N$ [°]	91.0	3.3	14.7
C=O [Å]	1.19	1.22	1.21
C-N [Å]	1.50	1.37	1.41
E_R [kcal/mol]	3.1[a]	16.6	7.0

(a) recalculated value, Szostak reported 0.95 kcal/mol for this compound[8]

References for computational methods

[1] Gaussian 09, Revision C.01, M. J. Frisch, G. W. Trucks, H. B. Schlegel, G. E. Scuseria, M. A. Robb, J. R. Cheeseman, G. Scalmani, V. Barone, G. A. Petersson, H. Nakatsuji, X. Li, M. Caricato, A. Marenich, J. Bloino, B. G. Janesko, R. Gomperts, B. Mennucci, H. P. Hratchian, J. V. Ortiz, A. F. Izmaylov, J. L. Sonnenberg, D. Williams-Young, F. Ding, F. Lipparini, F. Egidi, J. Goings, B. Peng, A. Petrone, T. Henderson, D. Ranasinghe, V. G. Zakrzewski, J. Gao, N. Rega, G. Zheng, W. Liang, M. Hada, M. Ehara, K. Toyota, R. Fukuda, J. Hasegawa, M. Ishida, T. Nakajima, Y. Honda, O. Kitao, H. Nakai, T. Vreven, K. Throssell, J. A. Montgomery, Jr., J. E. Peralta, F. Ogliaro, M. Bearpark, J. J. Heyd, E. Brothers, K. N. Kudin, V. N. Staroverov, T. Keith, R. Kobayashi, J. Normand, K. Raghavachari, A. Rendell, J. C. Burant, S. S. Iyengar, J. Tomasi, M. Cossi, J. M. Millam, M. Klene, C. Adamo, R. Cammi, J. W. Ochterski, R. L. Martin, K. Morokuma, O. Farkas, J. B. Foresman, and D. J. Fox, Gaussian, Inc., Wallingford CT, 2010.

[2] ThePyMOL Molecular Graphics System, Version 2.2.0a0 Schrödinger, LLC.

[3] (a) A.D. Becke, *J. Chem. Phys.* **1993**, *98*, 5648-5652; (b) C. Lee, W. Yang, R.G. Parr, *Phys. Rev. B* **1988**, *37*, 785-789.

[4] (a) A. J. H. Wachters, *J. Chem. Phys.* **1970,** *52*, 1033; (b) P. J. Hay, *J. Chem. Phys.* **1977**, *66*, 4377-4384; (c) K Raghavachari, J. S. Binkley, R. Seeger, J. A. Pople, *J. Chem. Phys.* **1980**, *72*, 650-654; (d) A. D. McLean, G. S. Chandler, *J. Chem. Phys.* **1980**, *72*, 5639-5648.

[5] S. A. Glover, A. A. Rosser, *J. Org. Chem.* **2012**, *77*, 5492-5502.

[6] Frog2: Efficient 3D conformation ensemble generator for small compounds. M. A. Miteva, F. Guyon, P. Tufféry, *Nucleic Acids Res.* **2010**, *38*, W622-W627.

[7] G. Meng, M. Szostak, *Eur. J. Org. Chem.* **2018**, 2352-2365.

[8] R. Szostak, M. Szostak, *Org. Lett.* **2018**, *20*, 1342-1345.

Coordinates of the calculated structures used in the COSNAR method.

AQ-AMIN
SCF done: -1087.40861233 au
Geometry:

O	1.011273 1.016037 0.030193
C	1.917834 0.276046 -0.275217
C	3.258603 0.622041 -0.831231
C	3.812682 1.856063 -1.130625
C	5.111558 1.868659 -1.650386
C	5.817210 0.679318 -1.858661
C	5.247086 -0.561425 -1.553739
C	3.961438 -0.562169 -1.037825
C	3.090929 -1.703112 -0.623290
O	3.336105 -2.888028 -0.665718
N	1.893760 -1.127578 -0.167402
C	0.732218 -1.865142 0.356925
C	1.084624 -2.668685 1.610596
C	0.111133 -2.730290 -0.769550
N	-1.151923 -3.324295 -0.401311
C	-2.373251 -2.709208 -0.493839
C	-3.538239 -3.529412 -0.260249
N	-3.332484 -4.841977 0.018231
C	-4.379533 -5.612505 0.234438
C	-5.710401 -5.142218 0.192117
C	-5.934139 -3.816602 -0.092496
C	-4.839529 -2.950992 -0.334529
C	-4.984049 -1.573670 -0.642205
C	-3.859466 -0.815339 -0.865457
C	-2.561874 -1.365142 -0.792817
H	0.017458 -1.085358 0.626032
H	1.833363 -3.433091 1.395724
H	0.184327 -3.156174 1.989696
H	1.468931 -2.010288 2.393270
H	0.805751 -3.532716 -1.023368
H	-0.008328 -2.107425 -1.664617
H	-1.205635 -4.320493 -0.243185
H	3.257644 2.771848 -0.966558
H	5.580377 2.814439 -1.896702
H	6.821940 0.721851 -2.263287
H	5.785024 -1.488355 -1.712260
H	-4.177934 -6.657962 0.453125
H	-6.530405 -5.825070 0.380639
H	-6.943078 -3.419020 -0.136367
H	-5.975148 -1.137991 -0.695651
H	-3.958657 0.239081 -1.099279
H	-1.711995 -0.716950 -0.963664

AQ-Carbonyl
SCF done: -1145.39436788 au
Geometry:

O	-0.151001 0.293561 -0.076643
C	0.948251 -0.154435 -0.301037
C	2.276650 0.516157 -0.191169
C	2.595086 1.815023 0.170576
C	3.949672 2.164255 0.187842
C	4.939133 1.234806 -0.148136
C	4.606468 -0.074166 -0.513038
C	3.262380 -0.410027 -0.526249
C	2.595396 -1.700762 -0.869471
O	3.095997 -2.746307 -1.216434
N	1.218041 -1.469688 -0.729761
C	0.179246 -2.495466 -0.859901
C	0.172038 -3.462423 0.323844
C	0.224274 -3.216983 -2.222942
C	0.590961 -2.384072 -3.454338
C	-0.142687 -2.815257 -4.696410
C	-1.544121 -2.557997 -4.795564
N	-2.161362 -1.937646 -3.750703
C	-3.452665 -1.699890 -3.839246
C	-4.244408 -2.051152 -4.957235
C	-3.641385 -2.679102 -6.018527
C	-2.253950 -2.956972 -5.969519
C	-1.556511 -3.608854 -7.018690
C	-0.211066 -3.851561 -6.900864
C	0.491003 -3.453272 -5.738608
O	-0.069795 -4.384007 -2.299794
H	-0.759868 -1.929915 -0.883394
H	1.109685 -4.017973 0.376666
H	-0.639122 -4.180709 0.206837
H	0.025873 -2.911016 1.255198
H	1.670177 -2.500958 -3.597439
H	0.397135 -1.329771 -3.246586
H	1.821333 2.527777 0.429208
H	4.239351 3.170886 0.466599
H	5.980246 1.535540 -0.124283
H	5.365884 -0.801246 -0.774395
H	-3.912219 -1.202300 -2.988564
H	-5.303829 -1.824291 -4.960702
H	-4.211875 -2.968295 -6.895410
H	-2.101047 -3.912892 -7.906536
H	0.324542 -4.353198 -7.698800
H	1.553189 -3.661502 -5.667682

AQ-Amide
SCF done: -1161.46797717 au
Geometry:

O	0.572585 0.870992 -0.235937
C	1.420563 0.033218 -0.433296
C	2.900266 0.194710 -0.515747
C	3.680517 1.334896 -0.409377
C	5.065895 1.178695 -0.521929
C	5.635498 -0.081234 -0.733443
C	4.838438 -1.225803 -0.838997
C	3.467416 -1.060332 -0.726240
C	2.370258 -2.070499 -0.788715
O	2.446473 -3.265530 -0.943038
N	1.172257 -1.341711 -0.633427
C	-0.162683 -1.933439 -0.502051
C	-0.282247 -2.841065 0.724754
C	-0.656538 -2.639742 -1.787113
N	-0.140424 -2.146244 -2.952574
C	-0.428740 -2.527275 -4.265953
C	0.310546 -1.825306 -5.276718
N	1.203343 -0.883171 -4.871454
C	1.887519 -0.231084 -5.788695
C	1.743556 -0.457767 -7.175723
C	0.846401 -1.407589 -7.600268
C	0.088753 -2.134490 -6.650302
C	-0.856966 -3.132264 -6.997209
C	-1.546210 -3.787152 -6.005820
C	-1.342591 -3.495052 -4.638009
O	-1.501291 -3.514183 -1.721510
H	-0.825508 -1.070963 -0.372543
H	0.392865 -3.693569 0.649606
H	-1.302094 -3.216726 0.797110
H	-0.047507 -2.272806 1.627500
H	0.563318 -1.417246 -2.907589
H	3.229708 2.305967 -0.243903
H	5.710620 2.046473 -0.442809
H	6.712760 -0.169994 -0.814809
H	5.270118 -2.206012 -1.001590
H	2.591837 0.515063 -5.430529
H	2.336716 0.113647 -7.879449
H	0.707738 -1.611254 -8.657095
H	-1.024031 -3.366166 -8.042787
H	-2.269491 -4.551635 -6.266222
H	-1.894844 -4.023658 -3.876296

AQ-Alkan

SCF done: -1071.36148551 au

O	0.807011	-0.243548	-2.019539
C	1.636111	-0.440369	-1.161313
C	2.905650	0.303221	-0.908465
C	3.460653	1.380907	-1.579052
C	4.679864	1.875571	-1.103405
C	5.308026	1.300491	0.006029
C	4.737717	0.212135	0.675240
C	3.530593	-0.269803	0.195366
C	2.680044	-1.399373	0.681196
O	2.882392	-2.135285	1.621396
N	1.574520	-1.448289	-0.181091
C	0.439295	-2.388782	-0.077044
C	-0.353911	-2.142365	1.211516
C	0.882736	-3.853355	-0.221443
C	1.592140	-4.159075	-1.553726
C	1.962393	-5.616140	-1.693744
C	0.980427	-6.579078	-2.090603
N	-0.285372	-6.147331	-2.359624
C	-1.184987	-7.032240	-2.729956
C	-0.924816	-8.416059	-2.866423
C	0.341177	-8.873127	-2.600394
C	1.345780	-7.958608	-2.199597
C	2.674742	-8.358206	-1.909171
C	3.602959	-7.421011	-1.530323
C	3.242043	-6.057789	-1.427179
H	-0.188006	-2.130110	-0.933735
H	0.245440	-2.374456	2.093772
H	-1.243258	-2.777316	1.221747
H	-0.680493	-1.101218	1.272636
H	-0.018502	-4.468753	-0.156880
H	1.526027	-4.127690	0.618614
H	2.504050	-3.559517	-1.630522
H	0.936716	-3.870951	-2.379314
H	2.965244	1.819282	-2.437040
H	5.146701	2.718174	-1.600529
H	6.251807	1.706596	0.351751
H	5.216017	-0.239525	1.536111
H	-2.181504	-6.649605	-2.938998
H	-1.717017	-9.087381	-3.175930
H	0.587486	-9.926212	-2.692661
H	2.942118	-9.406469	-1.992367
H	4.621413	-7.719785	-1.308754
H	3.996475	-5.337053	-1.128351

Urea-Amine
SCF done: -1202.01051501 au
Geometry:

O	0.780486	0.681507	-1.471404
C	1.733914	0.005325	-1.148434
C	3.172632	0.399323	-1.096593
C	3.780209	1.606449	-1.401254
C	5.169335	1.682218	-1.254723
C	5.910184	0.579575	-0.817277
C	5.286053	-0.634793	-0.512769
C	3.910214	-0.698387	-0.661549
C	2.967036	-1.830889	-0.428060
O	3.211776	-2.955995	-0.063621
N	1.683010	-1.331924	-0.732881
C	0.471101	-2.165615	-0.680085
C	-0.132556	-2.372826	-2.073254
C	-0.569983	-1.603496	0.307726
N	-0.108370	-1.576693	1.683995
C	-0.115529	-2.735792	2.481180
O	-0.484148	-3.846021	2.139671
C	0.348153	-0.484279	2.425174
C	0.645370	-0.981992	3.698350
N	0.373231	-2.337112	3.715279
C	0.556327	-3.126039	4.926209
C	1.645277	-2.461633	5.789627
C	1.392510	-0.956566	6.043811
C	1.135067	-0.223483	4.744564
C	1.329107	1.136242	4.470551
C	1.027436	1.661806	3.208145
C	0.533057	0.868013	2.161323
H	0.815260	-3.125238	-0.290234
H	-0.498599	-1.434603	-2.494247
H	-0.965586	-3.077292	-2.012359
H	0.612948	-2.792762	-2.751891
H	-1.447670	-2.252273	0.264365
H	-0.872269	-0.596901	0.018917
H	-0.390801	-3.183904	5.475920
H	0.837443	-4.141369	4.642340
H	1.709266	-2.996307	6.740694
H	2.610213	-2.581080	5.286726
H	0.530373	-0.837877	6.712515
H	2.250171	-0.523136	6.566180
H	3.197876	2.454838	-1.740122
H	5.682182	2.609364	-1.483676
H	6.985437	0.669603	-0.713728
H	5.850937	-1.494794	-0.173719
H	1.716867	1.791931	5.243050
H	1.183347	2.719233	3.028210
H	0.312907	1.300348	1.193459

Urea-Carbonyl
SCF done: -1259.98115367 au
Geometry:

O	0.596590 0.983921 -0.823937
C	1.487660 0.196848 -0.607038
C	2.939505 0.458467 -0.386171
C	3.636409 1.655253 -0.351610
C	5.016188 1.590232 -0.131469
C	5.661076 0.362030 0.047152
C	4.947141 -0.840338 0.011304
C	3.581320 -0.764937 -0.208097
C	2.560945 -1.850055 -0.308838
O	2.717874 -3.046665 -0.230698
N	1.332423 -1.197905 -0.507737
C	0.074872 -1.874115 -0.790837
C	-0.368333 -2.798421 0.353792
C	0.157277 -2.536432 -2.183044
C	-0.992994 -3.477490 -2.582692
C	-2.368713 -2.735950 -2.629469
O	-2.880594 -2.080135 -1.743198
H	-1.078040 -4.239717 -1.800085
C	-0.888126 -4.095539 -3.954671
C	-2.046791 -3.766785 -4.654789
N	-2.896758 -2.973608 -3.871816
C	-4.158514 -2.466032 -4.403485
C	-4.688733 -3.438106 -5.468368
C	-3.642652 -3.745230 -6.560309
C	-2.330378 -4.183169 -5.946285
C	-1.355510 -4.982972 -6.554895
C	-0.176423 -5.319483 -5.886057
C	0.070258 -4.877924 -4.577934
O	1.067000 -2.303927 -2.937762
H	-0.672937 -1.079815 -0.893204
H	0.281430 -3.669390 0.444719
H	-1.400398 -3.112472 0.198085
H	-0.332301 -2.242281 1.292553
H	-4.857349 -2.352208 -3.573460
H	-3.994299 -1.472601 -4.837805
H	-4.986125 -4.371736 -4.979720
H	-5.588012 -3.008471 -5.916748
H	-4.024111 -4.517802 -7.233914
H	-3.483447 -2.850050 -7.175103
H	3.127658 2.601128 -0.493495
H	5.597220 2.504803 -0.099873
H	6.732014 0.343818 0.214121
H	5.438135 -1.796653 0.145576
H	-1.519498 -5.347207 -7.564197
H	0.561956 -5.933820 -6.387799
H	0.993688 -5.138660 -4.075392

Urea-Amide

SCF done: -1276.05175591 au

Geometry:

C	5.995280	4.763078	7.832193
O	7.126599	4.719480	8.267858
N	4.763143	4.700892	8.579602
O	3.514126	4.262736	10.417105
C	3.670159	4.760622	7.672637
O	6.438212	1.862407	11.560844
C	2.287515	4.745009	7.809734
O	4.409367	5.619173	13.276219
C	1.532222	4.861077	6.633616
C	2.121817	4.985846	5.370867
C	3.513468	4.994792	5.228450
C	4.311450	5.115924	3.948037
C	5.715941	5.699185	4.224137
C	6.471145	4.955810	5.340150
N	5.613740	4.883233	6.519154
C	4.232447	4.879846	6.404073
C	4.611596	4.489863	9.963845
C	5.865536	4.580695	10.851716
C	6.368643	6.026739	11.005726
N	5.570572	3.942311	12.130324
C	5.860910	2.580543	12.343778
C	5.314639	2.249883	13.689706
C	5.348561	1.061934	14.402713
C	4.732153	1.047994	15.657849
C	4.107577	2.191368	16.167667
C	4.080477	3.384866	15.439055
C	4.694134	3.387525	14.196895
C	4.826669	4.489205	13.196346
H	1.818627	4.645351	8.775937
H	0.451384	4.854222	6.711252
H	1.490985	5.077991	4.492999
H	6.653414	3.984707	10.390874
H	5.832859	0.181642	13.997525
H	4.735398	0.137470	16.246038
H	3.636242	2.148600	17.142927
H	3.597723	4.275213	15.823583
H	4.410001	4.128639	3.479254
H	3.785522	5.748684	3.227916
H	5.617127	6.751681	4.508400
H	6.736617	3.941521	5.020791
H	7.393035	5.470517	5.613812
H	7.214442	6.036977	11.696255
H	5.593356	6.681972	11.397903
H	6.720817	6.391279	10.042113
H	6.321352	5.666496	3.315126

Urea-Alkane
SCF done: -1185.95104822 au
Geometry:

O	0.929319	-0.347844	-2.224306
C	1.671140	-0.521712	-1.282647
C	2.887963	0.252977	-0.903028
C	3.485554	1.340534	-1.519337
C	4.637407	1.864420	-0.922791
C	5.160388	1.307608	0.248858
C	4.548047	0.209706	0.862830
C	3.407979	-0.300622	0.263942
C	2.537214	-1.445028	0.669128
O	2.642977	-2.163318	1.635488
N	1.531910	-1.529135	-0.313169
C	0.408105	-2.485701	-0.310717
C	-0.472008	-2.301372	0.932053
C	0.892373	-3.937679	-0.458338
C	1.656948	-4.255442	-1.761072
C	2.260973	-5.680524	-1.655891
O	3.008393	-6.097511	-0.793822
H	2.495775	-3.557416	-1.862422
C	0.841041	-4.327068	-3.038621
C	0.948304	-5.628748	-3.535506
N	1.771266	-6.409855	-2.717571
C	2.039235	-7.810067	-3.023713
C	1.931055	-8.033560	-4.539821
C	0.598021	-7.513809	-5.117533
C	0.357163	-6.076525	-4.707962
C	-0.393832	-5.129986	-5.415540
C	-0.518617	-3.819051	-4.953059
C	0.103888	-3.401906	-3.765707
H	-0.175529	-2.208259	-1.189682
H	0.066670	-2.567226	1.842599
H	-1.355305	-2.939745	0.852184
H	-0.810654	-1.265645	1.013632
H	0.010244	-4.583698	-0.401805
H	1.536103	-4.195736	0.385816
H	3.033671	-8.055603	-2.647528
H	1.319874	-8.444658	-2.491177
H	2.762116	-7.519893	-5.034188
H	2.042876	-9.101021	-4.746526
H	0.604718	-7.602135	-6.207642
H	-0.225229	-8.146437	-4.760930
H	3.072972	1.764188	-2.427058
H	5.134639	2.715107	-1.374532
H	6.055251	1.735116	0.686417
H	4.945530	-0.229279	1.769995
H	-0.875126	-5.418145	-6.345022
H	-1.094596	-3.103831	-5.528786
H	0.025795	-2.368864	-3.448091

12) NMR data

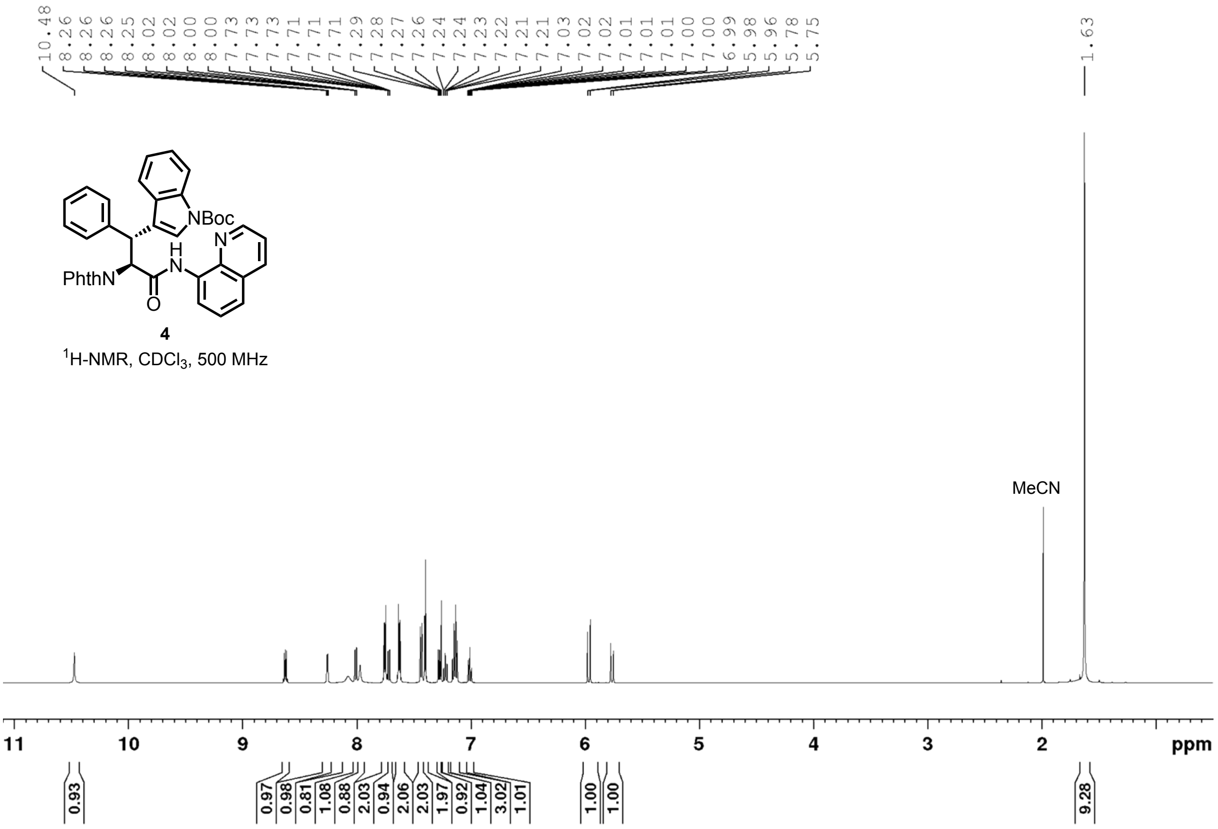
10.48
8.26
8.26
8.25
8.02
8.02
8.00
8.00
7.73
7.73
7.73
7.71
7.71
7.71
7.29
7.28
7.27
7.26
7.24
7.24
7.23
7.22
7.21
7.21
7.03
7.02
7.02
7.01
7.01
7.00
7.00
6.99
5.98
5.96
5.78
5.75
1.63
NBoc
H
N
N
PhthN
O
4
^{1}H-NMR, $CDCl_3$, 500 MHz
MeCN
11 10 9 8 7 6 5 4 3 2 ppm
0.93
0.97
0.98
0.81
1.08
0.88
2.03
0.94
2.06
2.03
1.97
0.92
1.04
3.02
1.01
1.00
1.00
9.28

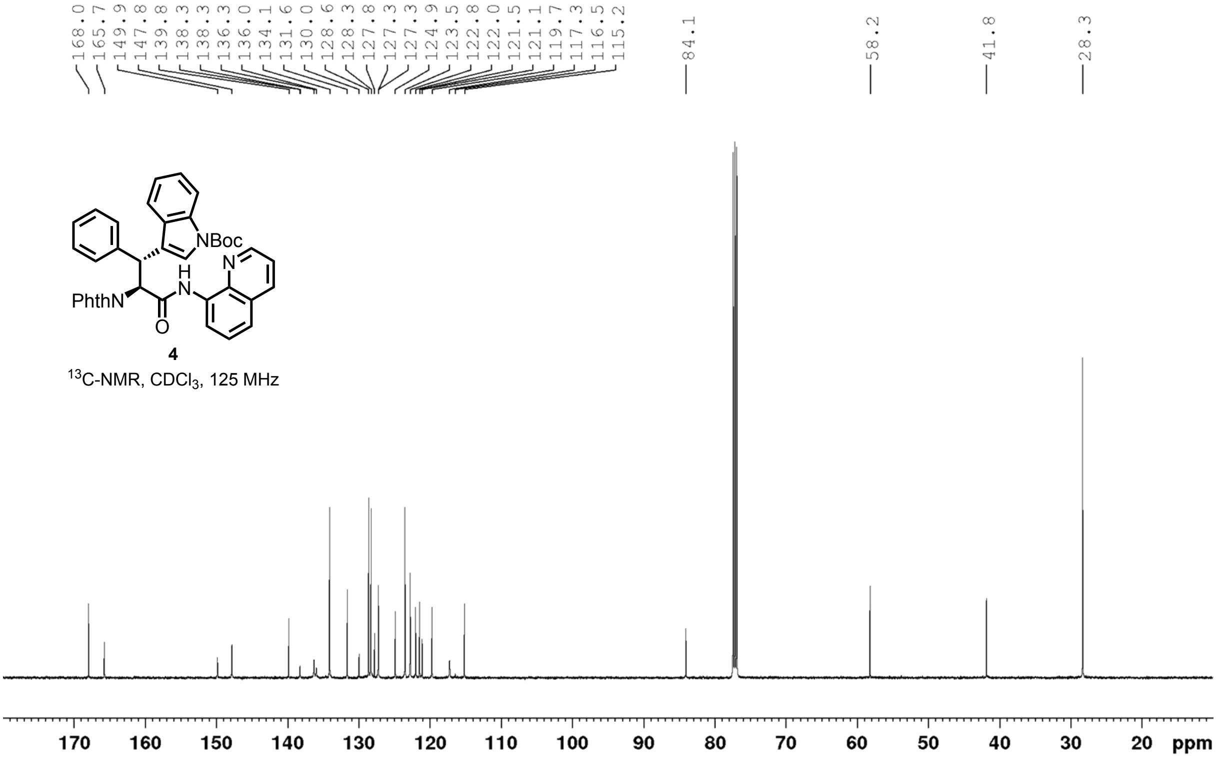
168.0
165.7
149.9
147.8
139.8
138.3
138.3
136.3
136.0
134.1
131.6
130.0
128.6
128.3
127.8
127.3
127.3
124.9
123.5
122.8
122.0
121.5
121.1
119.7
117.3
116.5
115.2
84.1
58.2
41.8
28.3
NBoc
H
N
N
PhthN
O
4
^{13}C-NMR, $CDCl_3$, 125 MHz
170 160 150 140 130 120 110 100 90 80 70 60 50 40 30 20 ppm

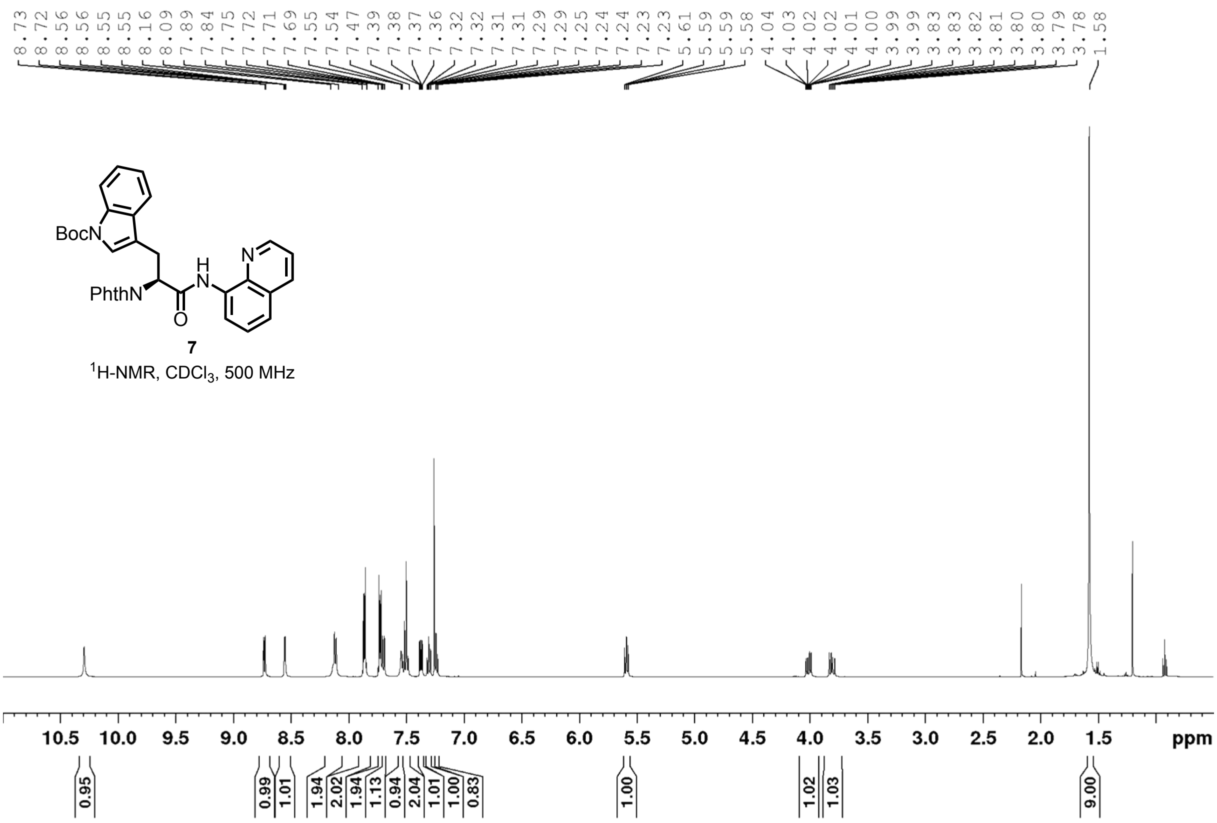
8.73 8.72 8.56 8.56 8.55 8.55 8.16 8.09 7.89 7.84 7.75 7.72 7.71 7.69 7.55 7.54 7.47 7.39 7.38 7.37 7.36 7.32 7.32 7.31 7.31 7.29 7.29 7.25 7.24 7.24 7.23 7.23 5.61 5.59 5.59 5.58 4.04 4.03 4.02 4.02 4.01 4.00 3.99 3.99 3.83 3.83 3.82 3.81 3.80 3.79 3.78 1.58
BocN
H
N
PhthN
O
7
1H-NMR, CDCl3, 500 MHz
10.5 10.0 9.5 9.0 8.5 8.0 7.5 7.0 6.5 6.0 5.5 5.0 4.5 4.0 3.5 3.0 2.5 2.0 1.5 ppm
0.95 0.99 1.01 1.94 2.02 1.94 1.13 0.94 2.04 1.01 1.00 0.83 1.00 1.02 1.03 9.00

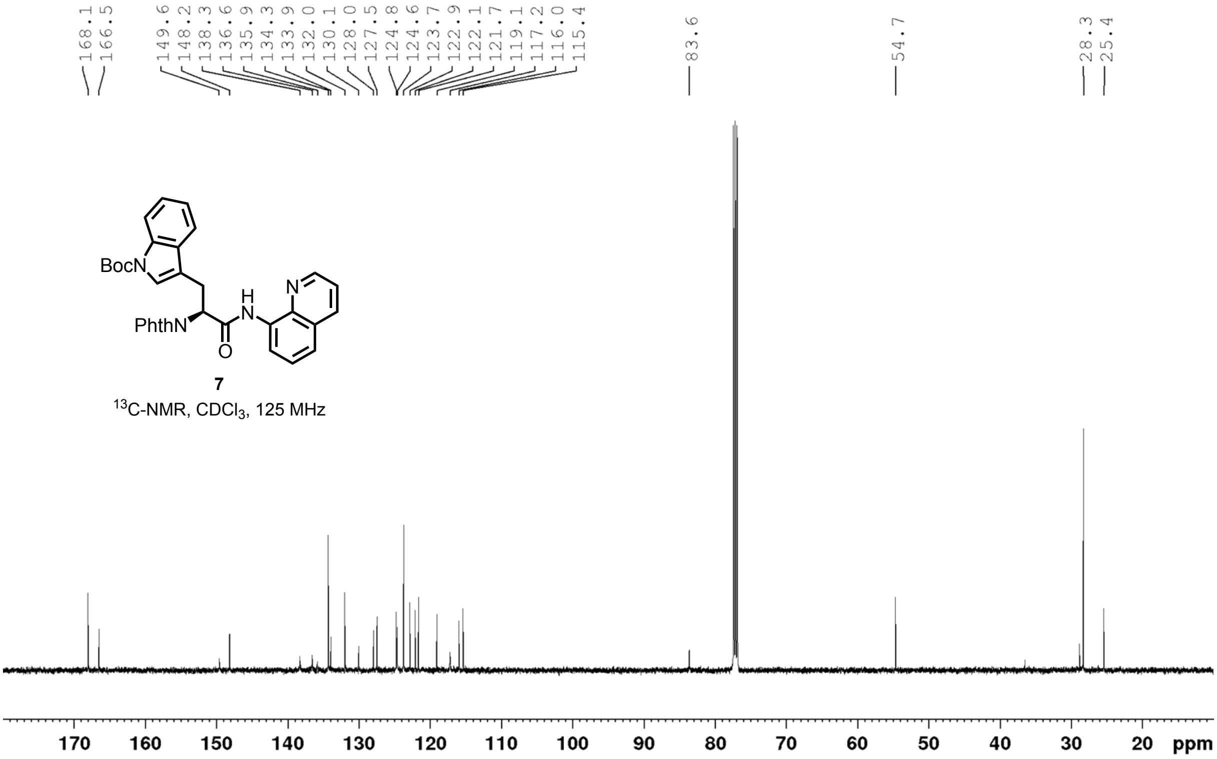
168.1 166.5 149.6 148.2 138.3 136.6 135.9 134.3 133.9 132.0 130.1 128.0 127.5 124.8 124.6 123.7 122.9 122.1 121.7 119.1 117.2 116.0 115.4 83.6 54.7 28.3 25.4
BocN
H
N
PhthN
O
7
13C-NMR, CDCl3, 125 MHz
170 160 150 140 130 120 110 100 90 80 70 60 50 40 30 20 ppm

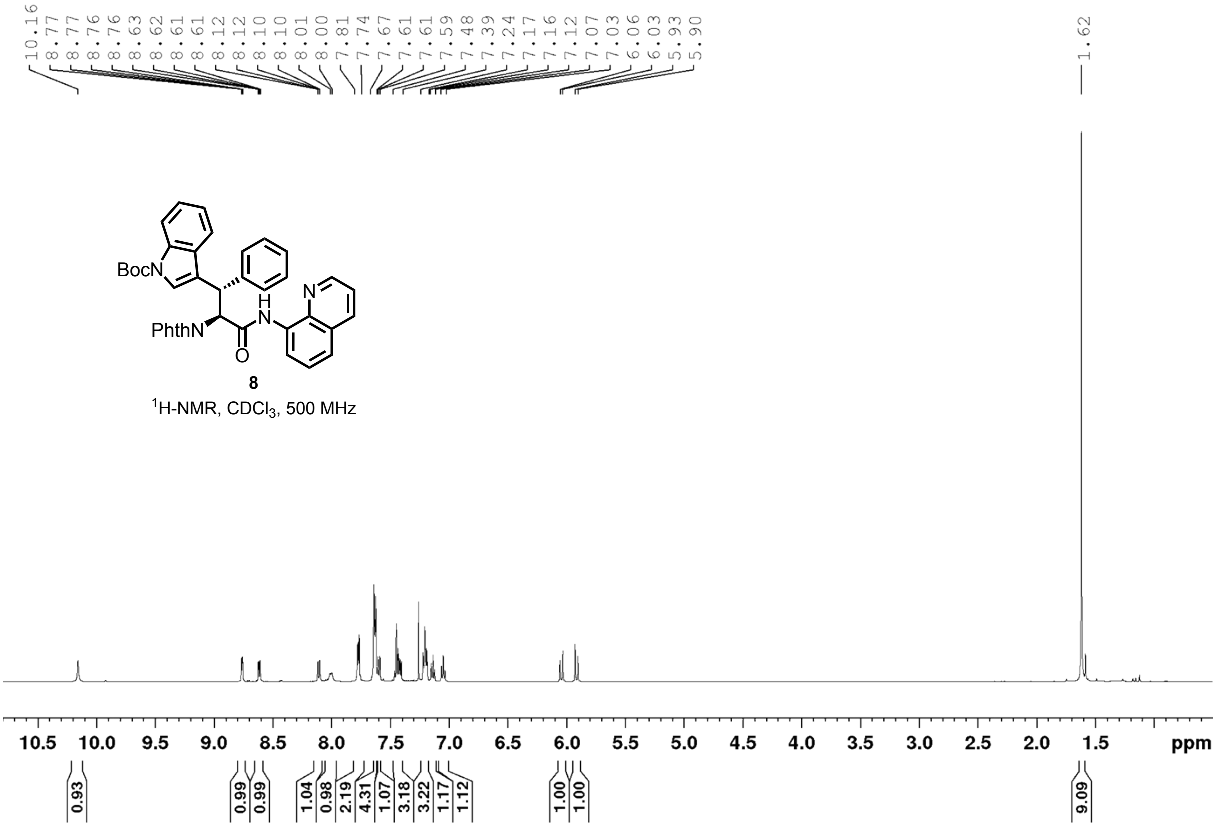

8

^{1}H-NMR, $CDCl_3$, 500 MHz

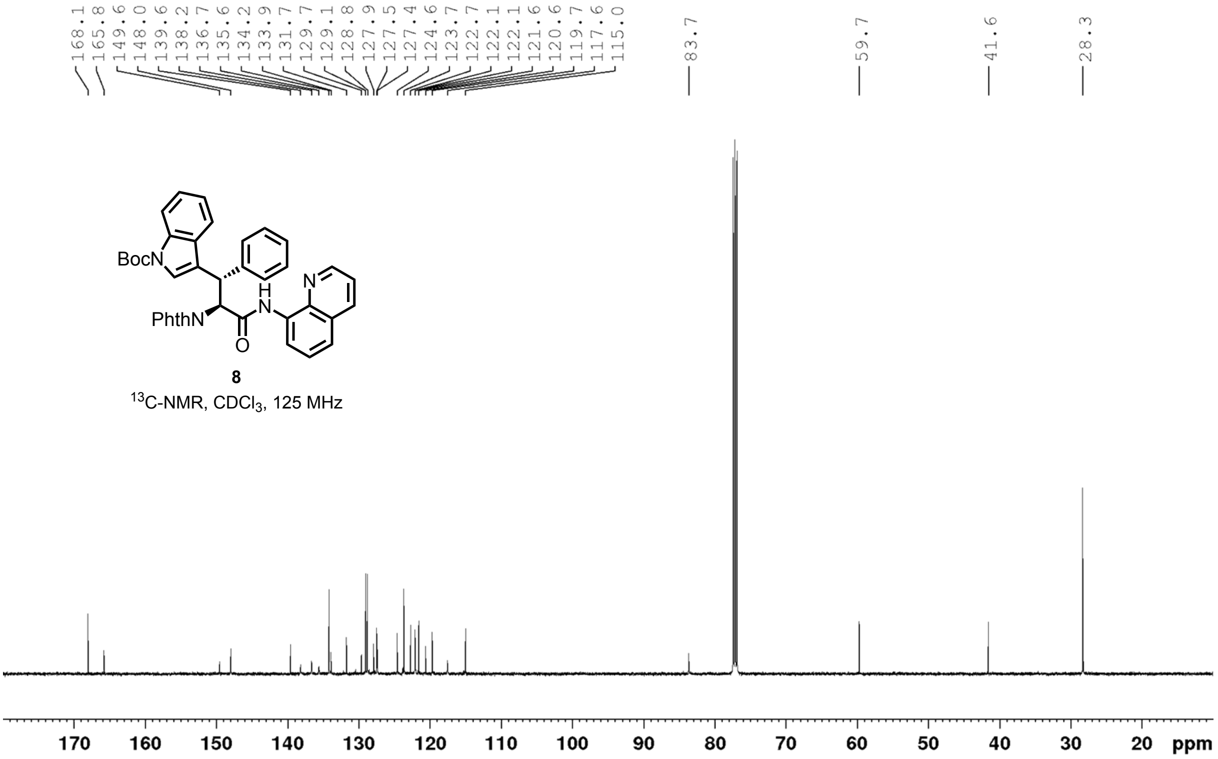

8

^{13}C-NMR, $CDCl_3$, 125 MHz

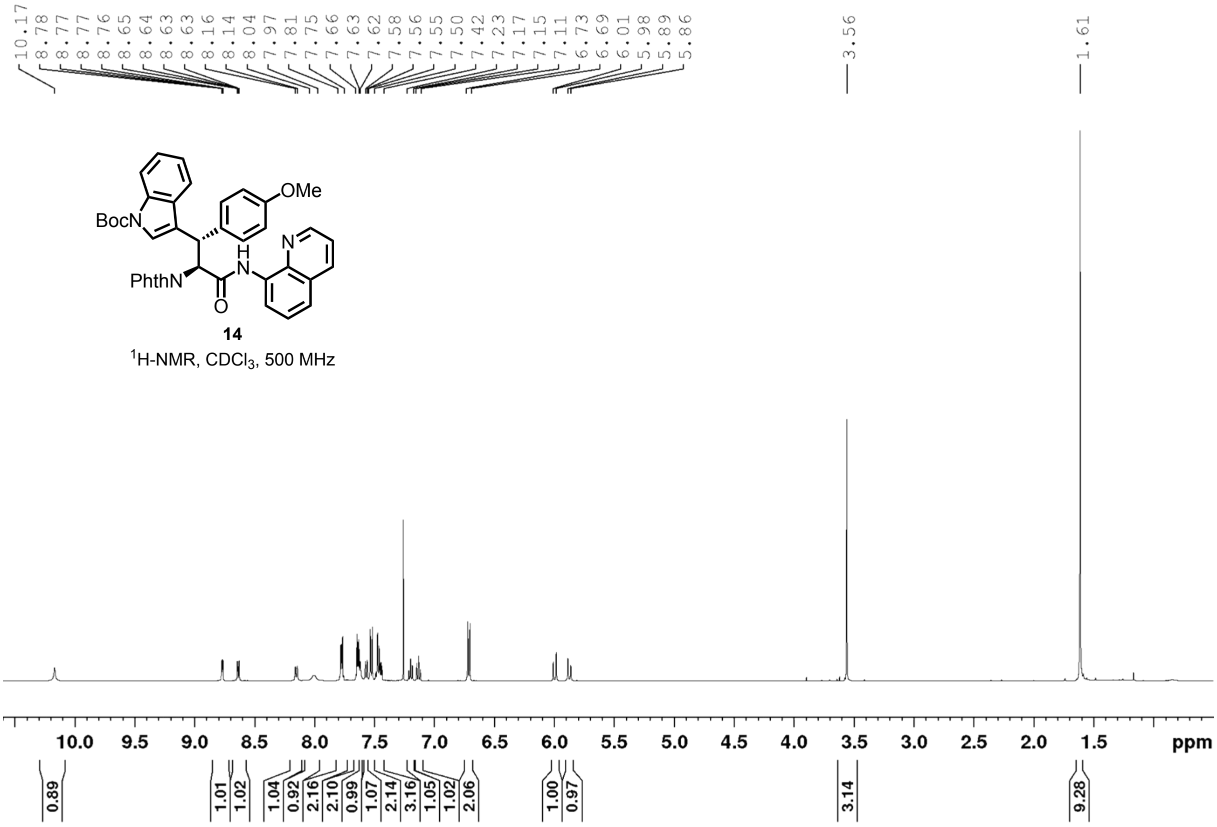

14

^{1}H-NMR, $CDCl_3$, 500 MHz

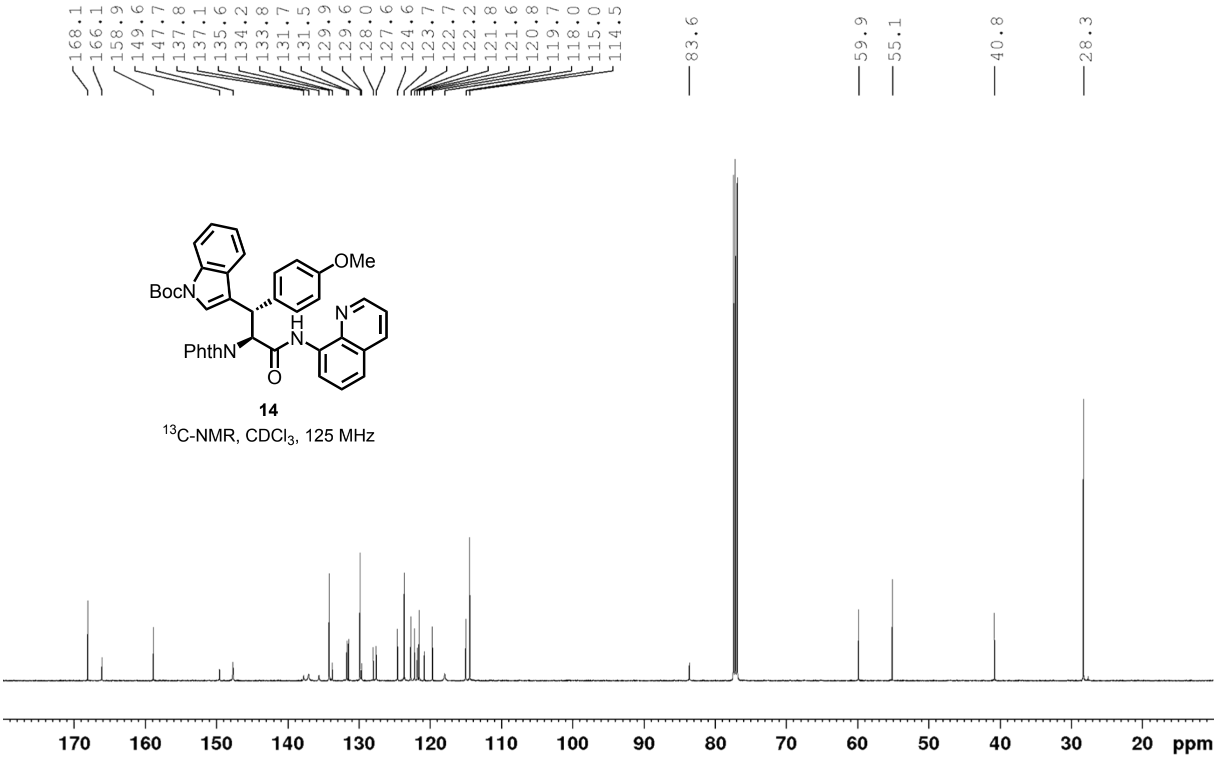

14

^{13}C-NMR, $CDCl_3$, 125 MHz

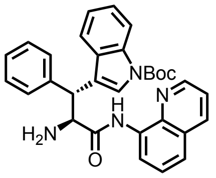

23

^{1}H-NMR, DMSO, 500 MHz

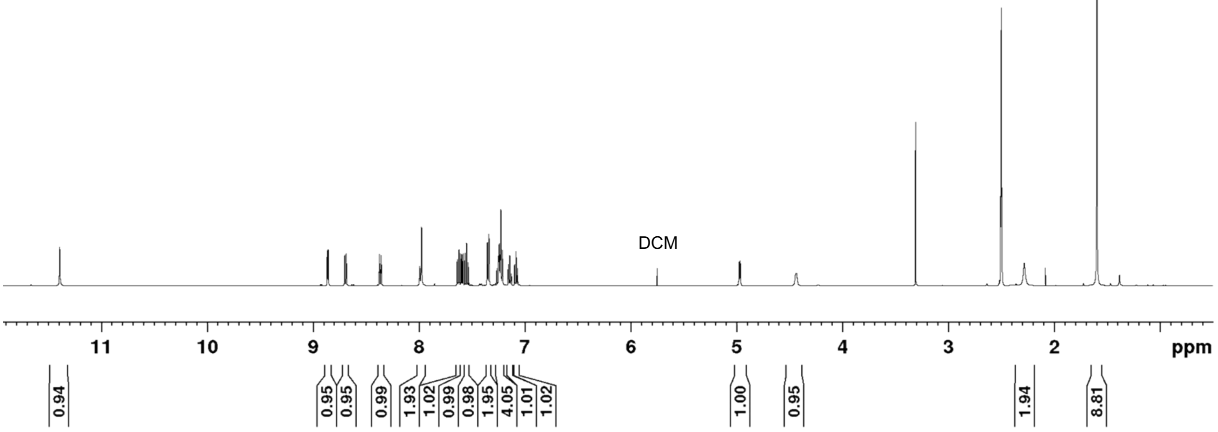

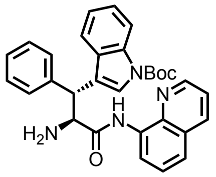

23

^{13}C-NMR, DMSO, 125 MHz

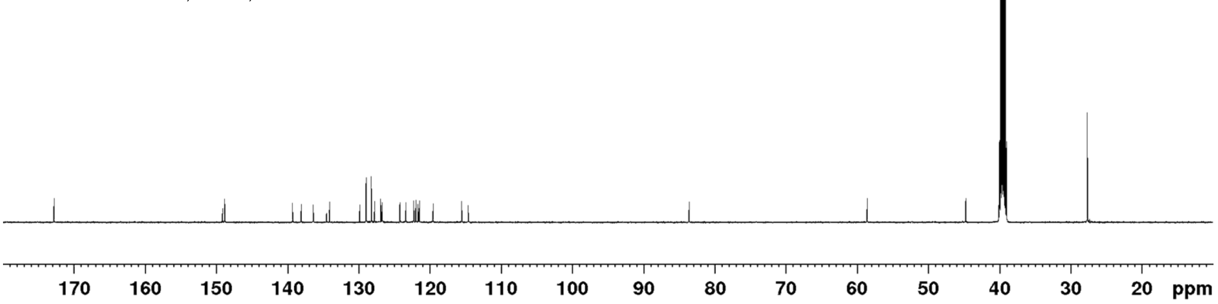

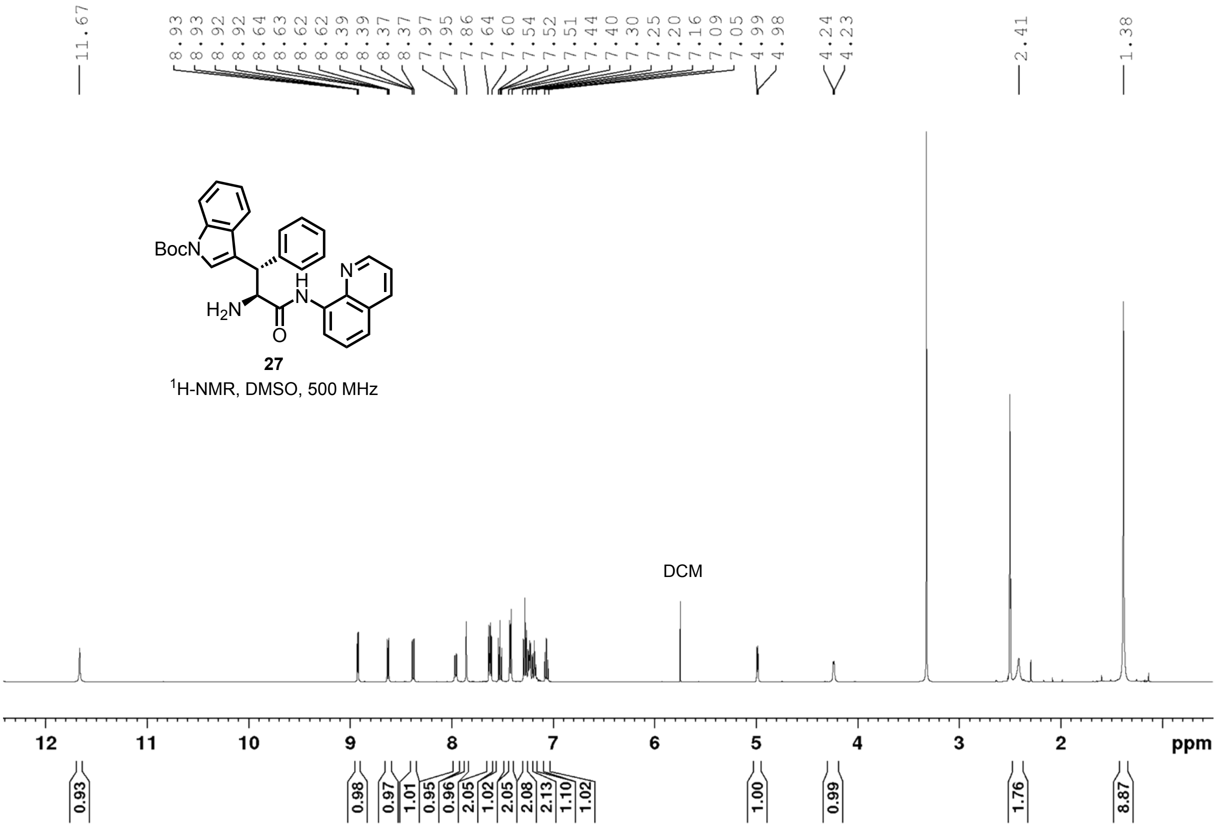

27

^{1}H-NMR, DMSO, 500 MHz

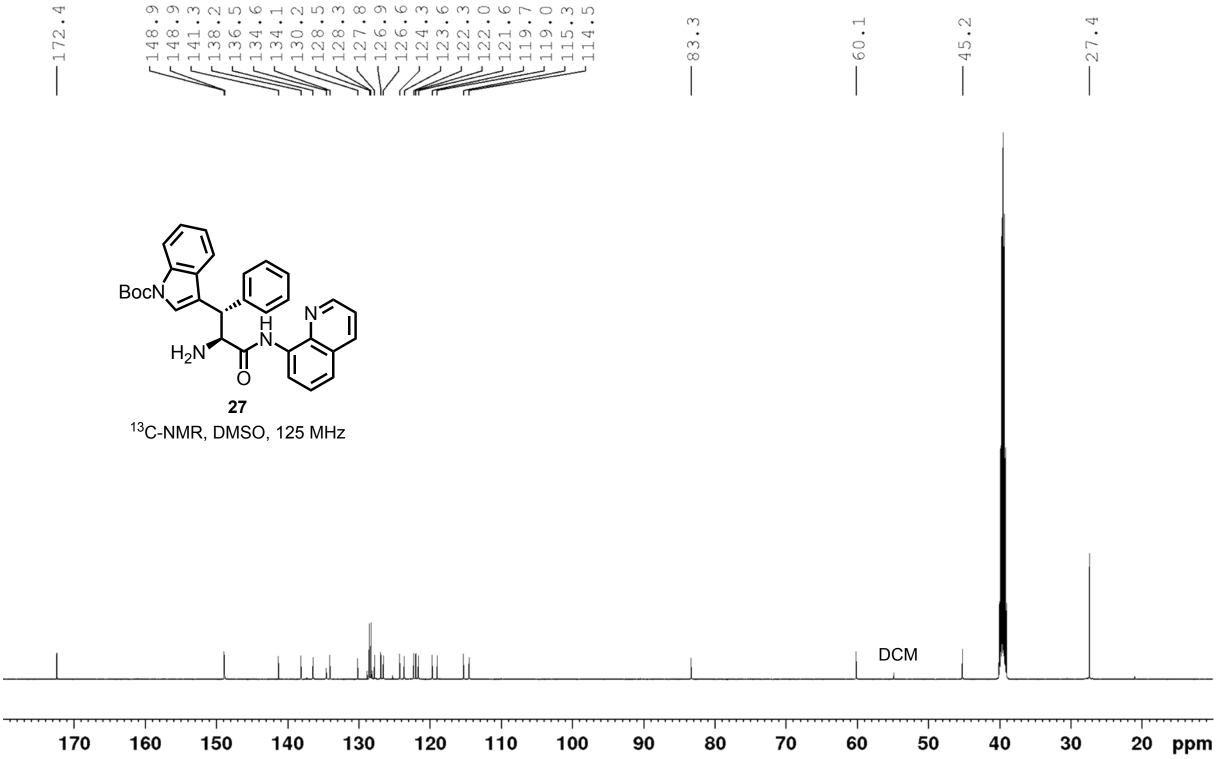

27

^{13}C-NMR, DMSO, 125 MHz

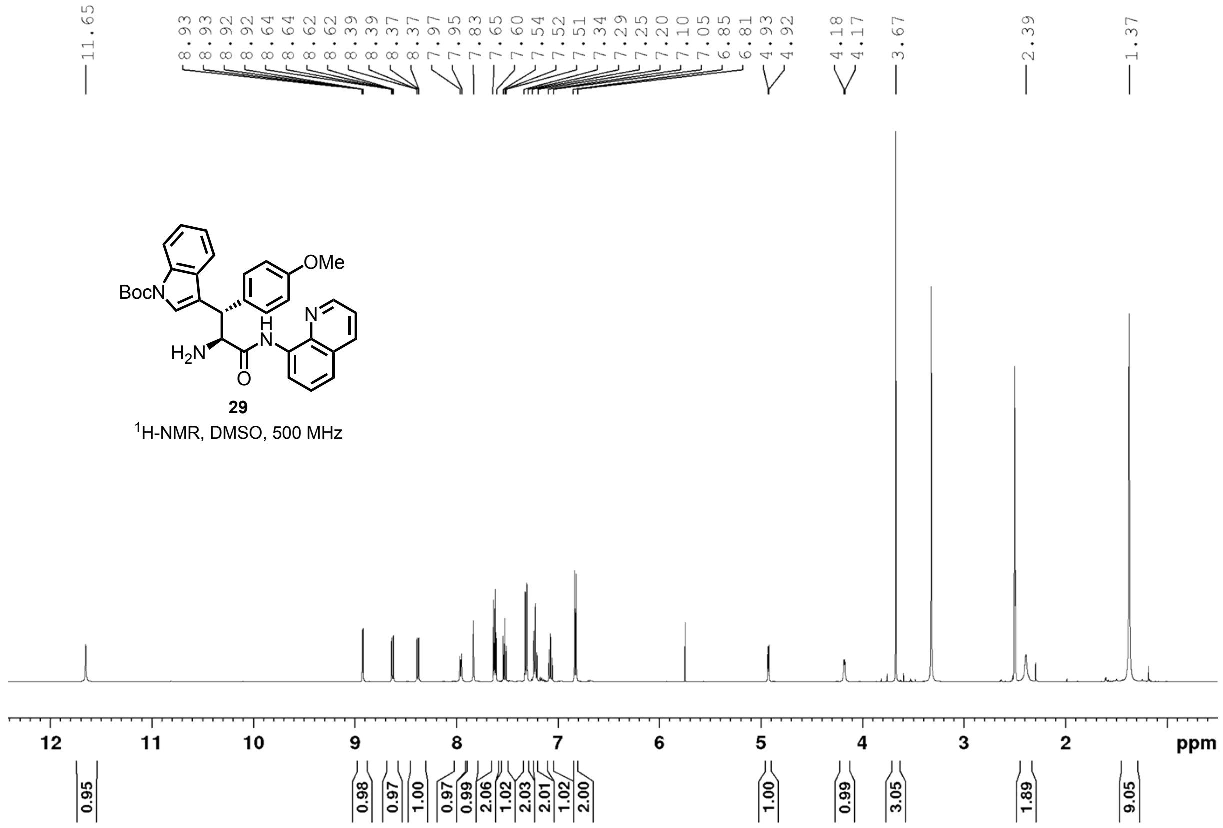

29
^{1}H-NMR, DMSO, 500 MHz

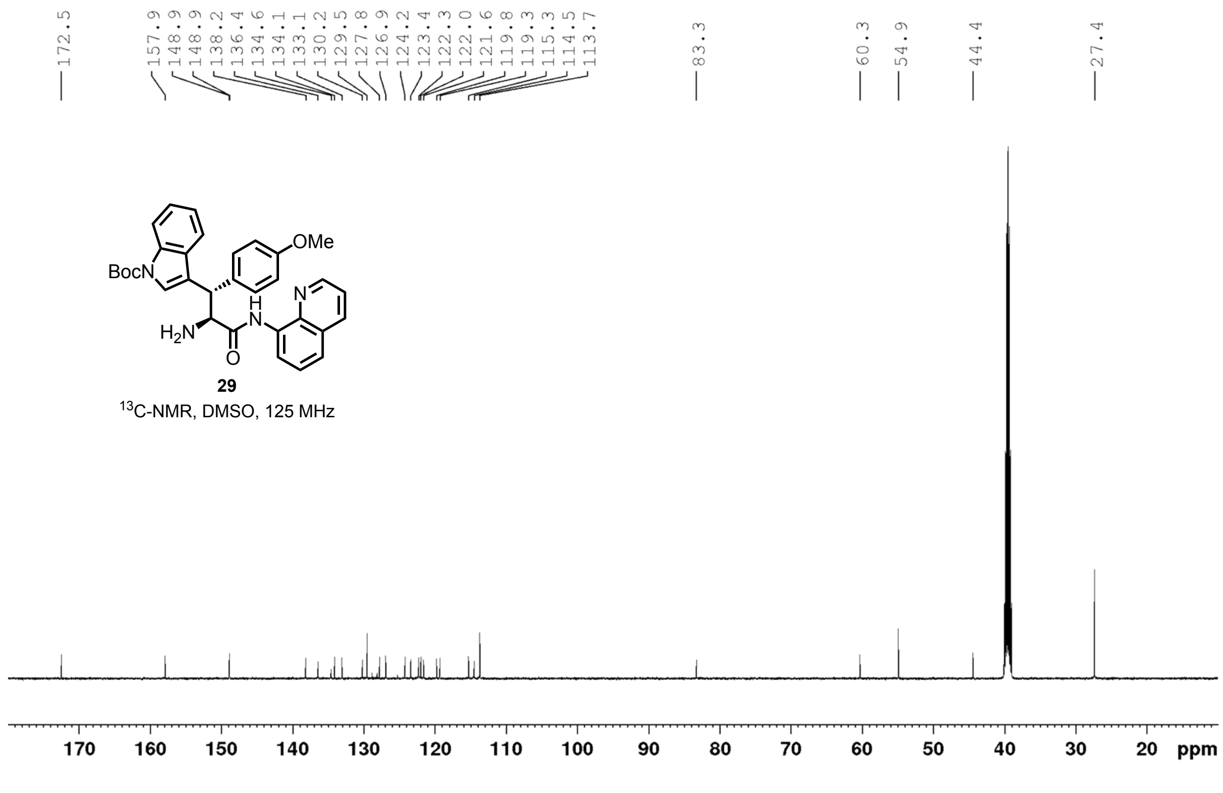

29
^{13}C-NMR, DMSO, 125 MHz

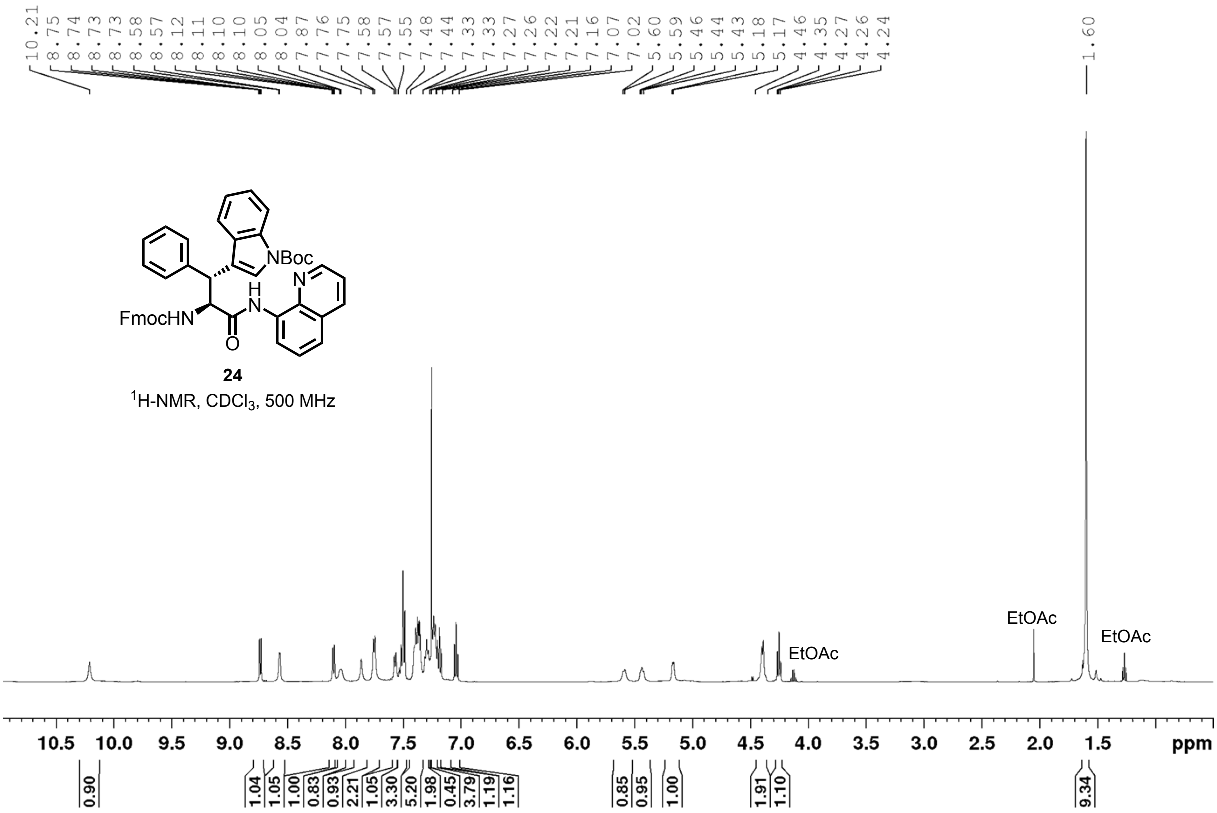
10.21 8.75 8.74 8.73 8.73 8.58 8.57 8.12 8.11 8.10 8.05 8.04 7.87 7.76 7.75 7.58 7.57 7.55 7.48 7.44 7.33 7.33 7.27 7.26 7.22 7.21 7.16 7.07 7.02 5.60 5.59 5.46 5.44 5.43 5.18 5.17 4.46 4.35 4.27 4.26 4.24
1.60
NBoc
N
H
N
FmocHN
O
24
^{1}H-NMR, $CDCl_3$, 500 MHz
EtOAc
EtOAc
EtOAc
10.5 10.0 9.5 9.0 8.5 8.0 7.5 7.0 6.5 6.0 5.5 5.0 4.5 4.0 3.5 3.0 2.5 2.0 1.5 ppm
0.90
1.04 1.05 1.00 0.83 0.93 2.21 1.05 3.30 5.20 1.98 0.45 3.79 1.19 1.16
0.85 0.95 1.00
1.91 1.10
9.34

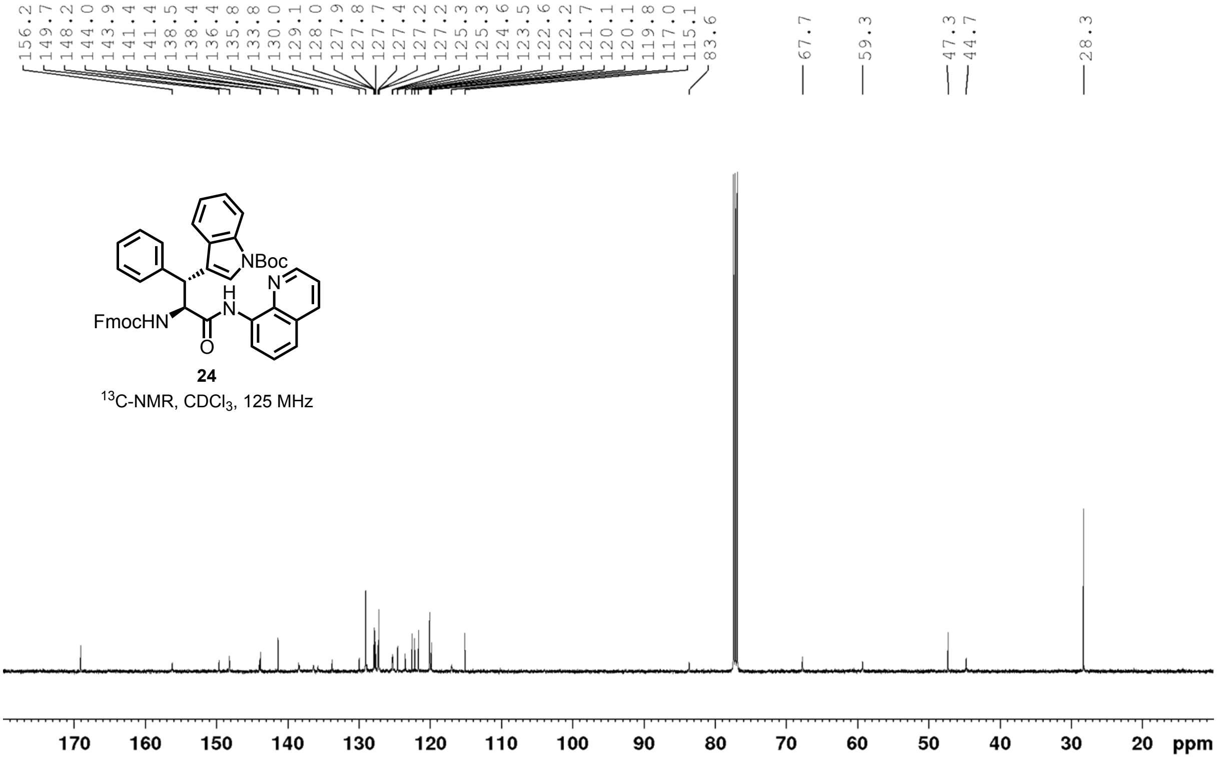
156.2 149.7 148.2 144.0 143.9 141.4 138.5 138.4 136.4 135.8 133.8 130.0 129.1 128.0 127.9 127.8 127.7 127.4 127.2 127.2 125.3 125.3 124.6 123.5 122.6 122.2 121.7 120.1 120.1 119.8 117.0 115.1 83.6
67.7
59.3
47.3 44.7
28.3
NBoc
N
H
N
FmocHN
O
24
^{13}C-NMR, $CDCl_3$, 125 MHz
170 160 150 140 130 120 110 100 90 80 70 60 50 40 30 20 ppm

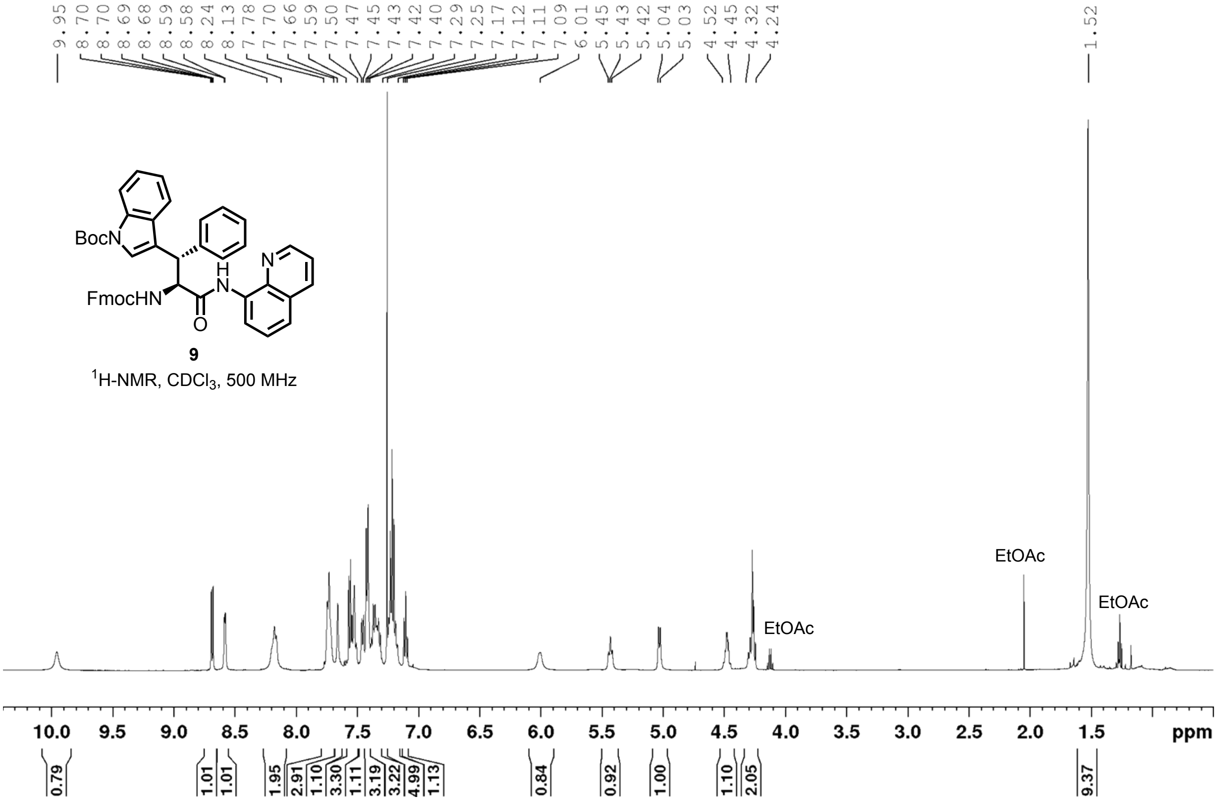
9.95 8.70 8.69 8.68 8.59 8.58 8.24 8.13 7.78 7.70 7.66 7.59 7.50 7.47 7.45 7.43 7.42 7.40 7.29 7.25 7.17 7.12 7.11 7.09 6.01 5.45 5.43 5.42 5.04 5.03 4.52 4.45 4.32 4.24 1.52
BocN
FmocHN
H
N
N
O
9
1H-NMR, CDCl3, 500 MHz
EtOAc
EtOAc
EtOAc
10.0 9.5 9.0 8.5 8.0 7.5 7.0 6.5 6.0 5.5 5.0 4.5 4.0 3.5 3.0 2.5 2.0 1.5 ppm
0.79 1.01 1.01 1.95 2.91 1.10 3.30 1.11 3.19 3.22 4.99 1.13 0.84 0.92 1.00 1.10 2.05 9.37

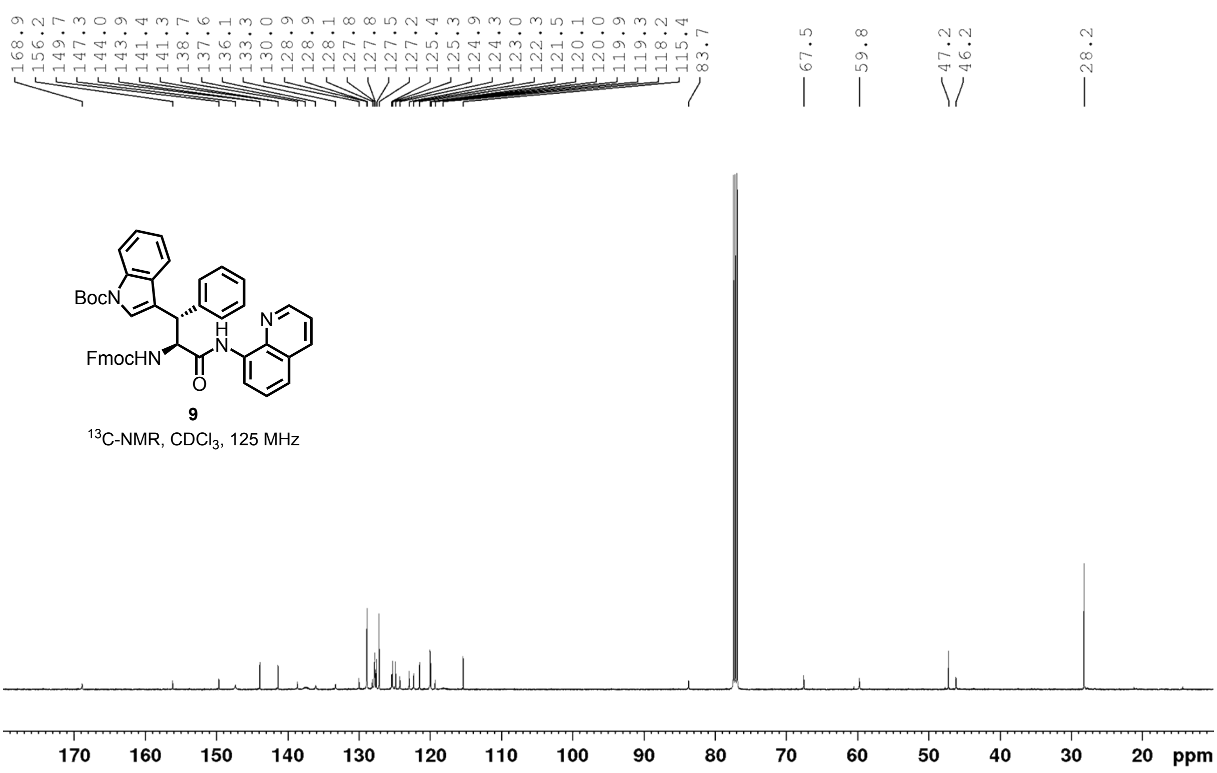
168.9 156.2 149.7 147.3 144.0 143.9 141.4 141.3 138.7 137.6 136.1 133.3 130.0 128.9 128.9 128.1 127.8 127.8 127.5 127.2 125.4 125.3 124.9 124.3 123.0 122.3 121.5 120.1 120.0 119.9 119.3 118.2 115.4 83.7 67.5 59.8 47.2 46.2 28.2
BocN
FmocHN
H
N
N
O
9
13C-NMR, CDCl3, 125 MHz
170 160 150 140 130 120 110 100 90 80 70 60 50 40 30 20 ppm

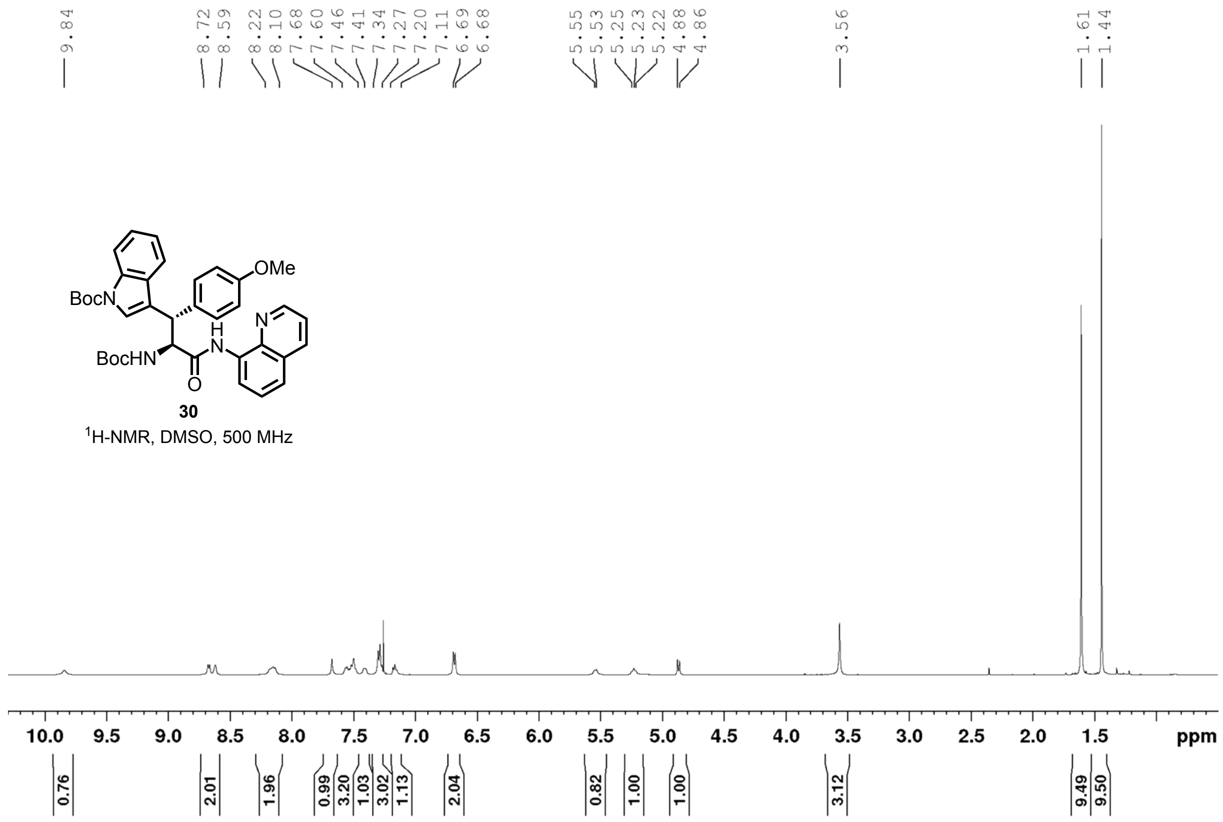
9.84
8.72
8.59
8.22
8.10
7.68
7.60
7.46
7.41
7.34
7.27
7.20
7.11
6.69
6.68
5.55
5.53
5.25
5.23
5.22
4.88
4.86
3.56
1.61
1.44
OMe
BocN
H
N
BocHN
O
30
^{1}H-NMR, DMSO, 500 MHz
0.76
2.01
1.96
0.99
3.20
1.03
3.02
1.13
2.04
0.82
1.00
1.00
3.12
9.49
9.50
10.0 9.5 9.0 8.5 8.0 7.5 7.0 6.5 6.0 5.5 5.0 4.5 4.0 3.5 3.0 2.5 2.0 1.5 ppm

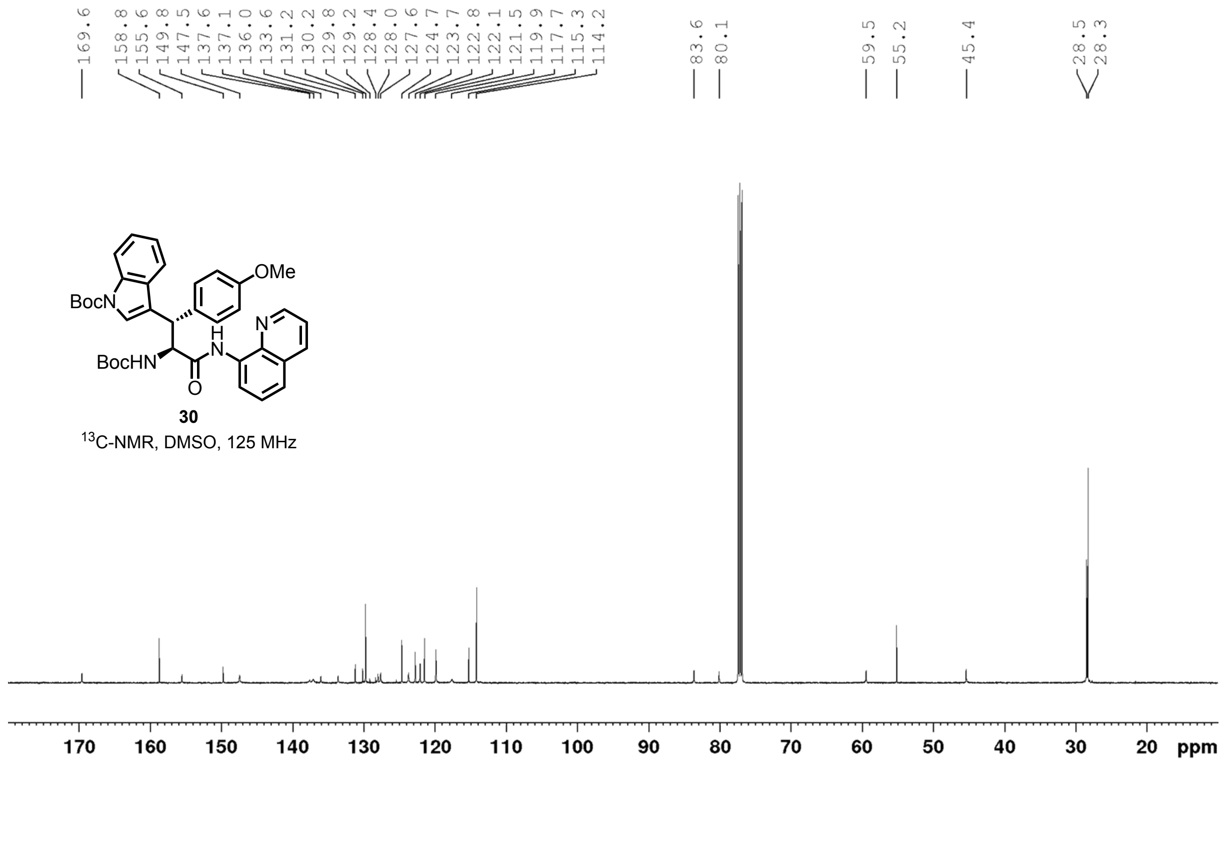
169.6
158.8
155.6
149.8
147.5
137.6
137.1
136.0
133.6
131.2
130.2
129.8
129.2
128.4
128.0
127.6
124.7
123.7
122.8
122.1
121.5
119.9
117.7
115.3
114.2
83.6
80.1
59.5
55.2
45.4
28.5
28.3
OMe
BocN
H
N
BocHN
O
30
^{13}C-NMR, DMSO, 125 MHz
170 160 150 140 130 120 110 100 90 80 70 60 50 40 30 20 ppm

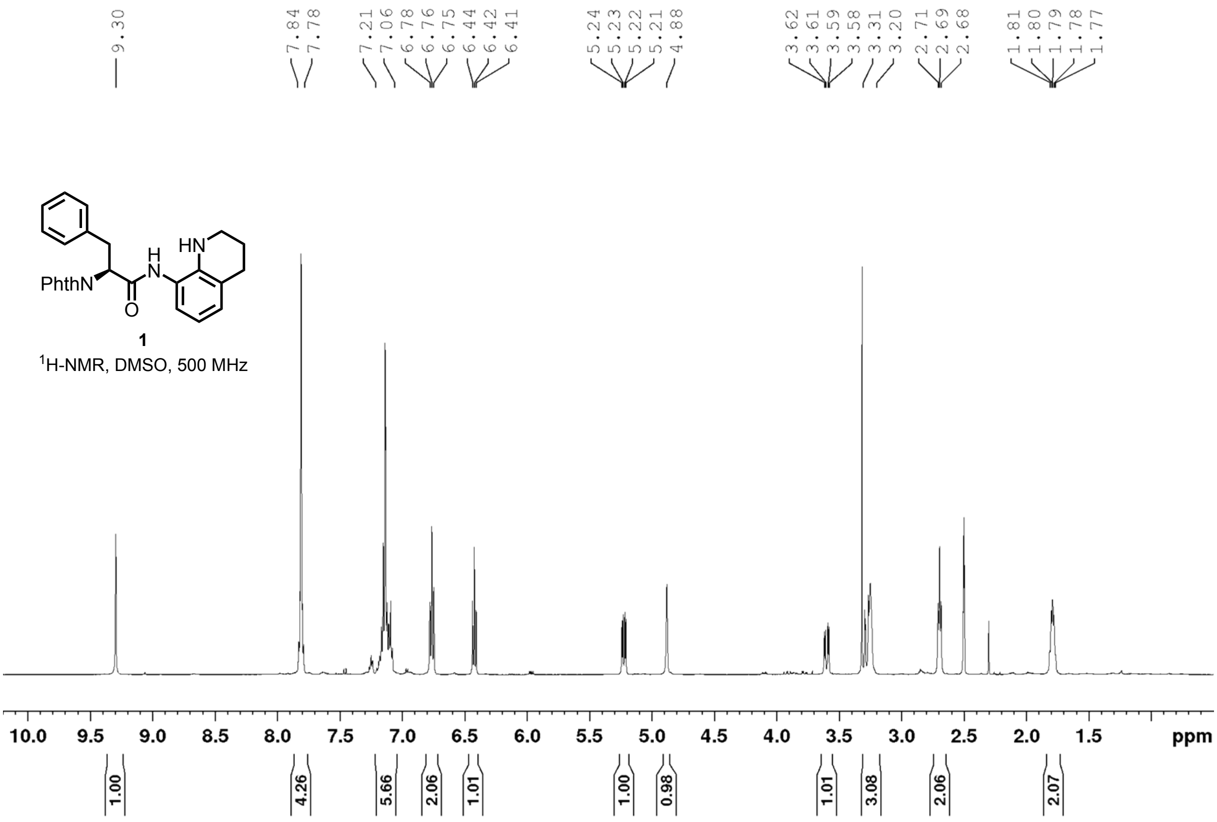
9.30
7.84
7.78
7.21
7.06
6.78
6.76
6.75
6.44
6.42
6.41
5.24
5.23
5.22
5.21
4.88
3.62
3.61
3.59
3.58
3.31
3.20
2.71
2.69
2.68
1.81
1.80
1.79
1.78
1.77
H
HN
PhthN
N
O
1
¹H-NMR, DMSO, 500 MHz
10.0 9.5 9.0 8.5 8.0 7.5 7.0 6.5 6.0 5.5 5.0 4.5 4.0 3.5 3.0 2.5 2.0 1.5 ppm
1.00
4.26
5.66
2.06
1.01
1.00
0.98
1.01
3.08
2.06
2.07

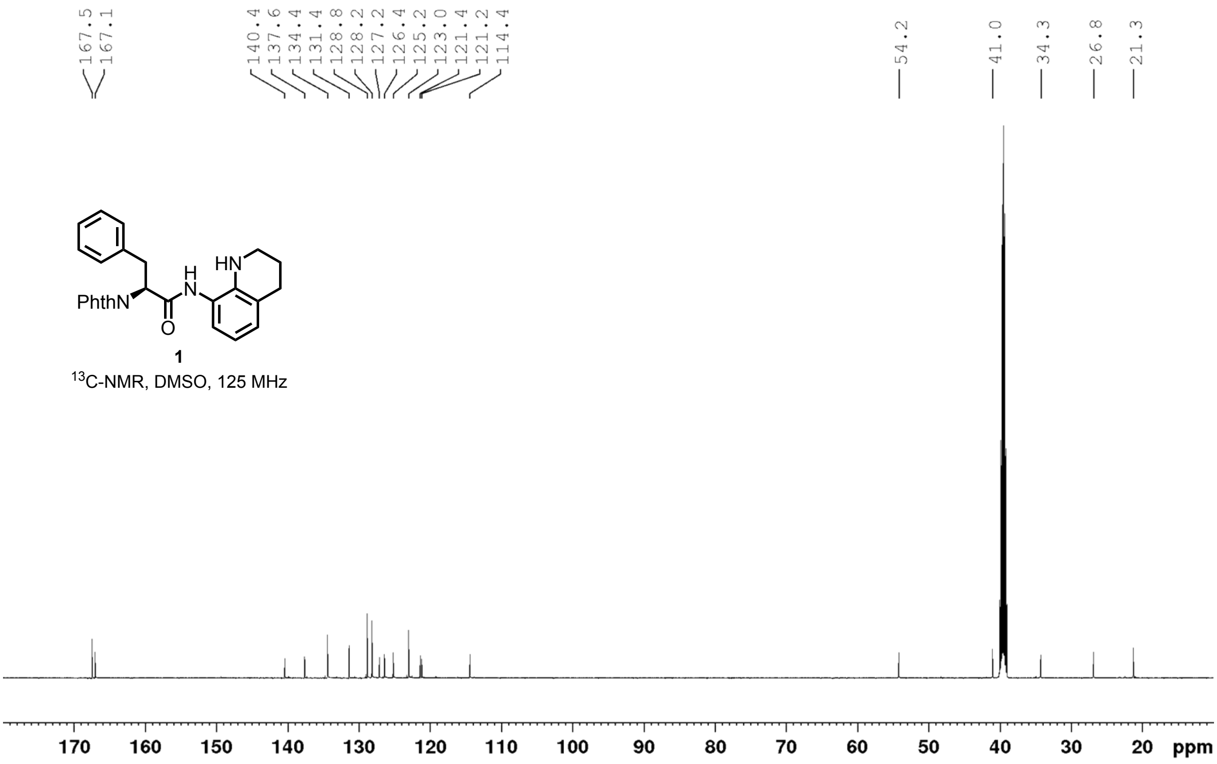
167.5
167.1
140.4
137.6
134.4
131.4
128.8
128.2
127.2
126.4
125.2
123.0
121.4
121.2
114.4
54.2
41.0
34.3
26.8
21.3
H
HN
PhthN
N
O
1
¹³C-NMR, DMSO, 125 MHz
170 160 150 140 130 120 110 100 90 80 70 60 50 40 30 20 ppm

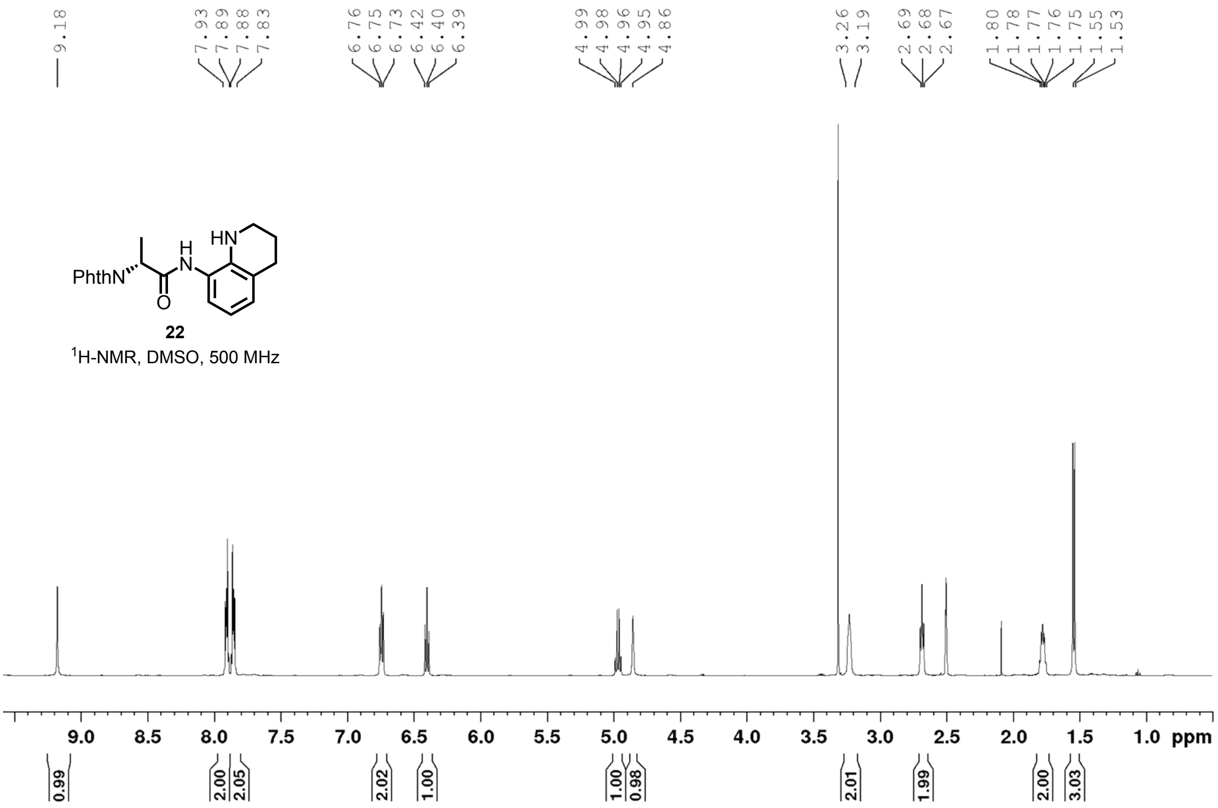

22

^{1}H-NMR, DMSO, 500 MHz

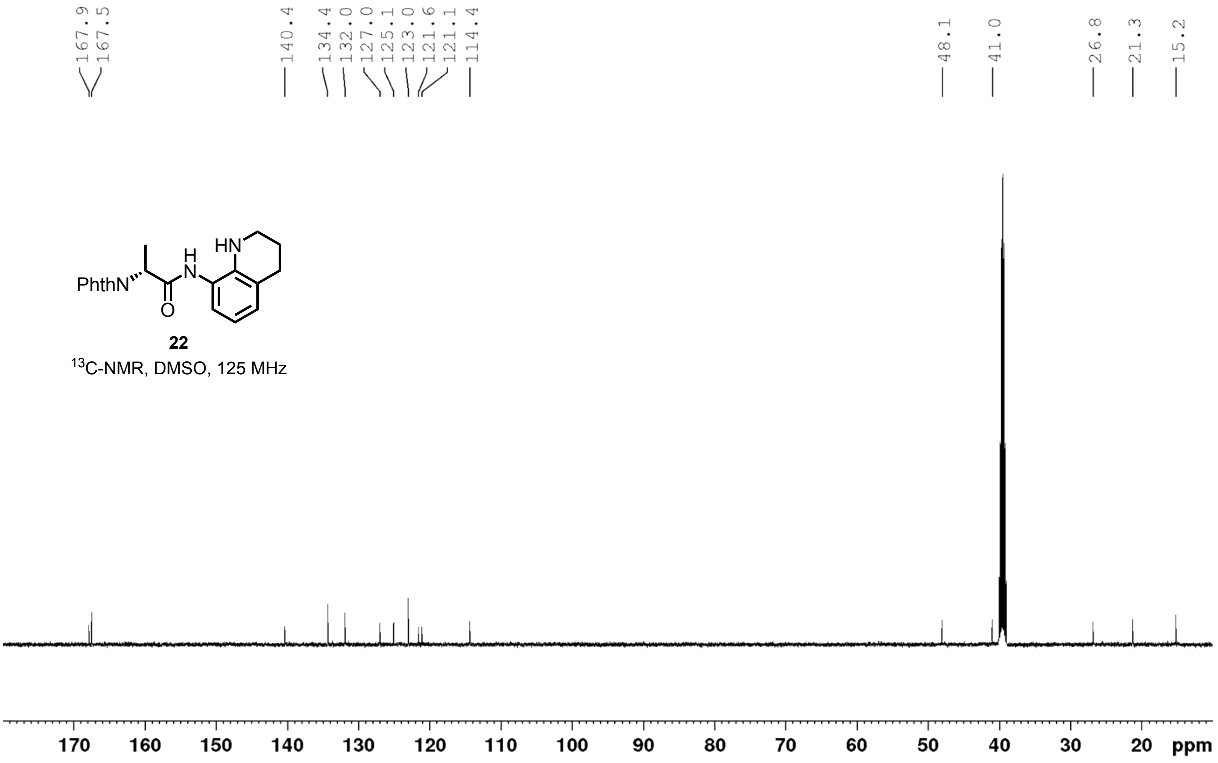

22

^{13}C-NMR, DMSO, 125 MHz

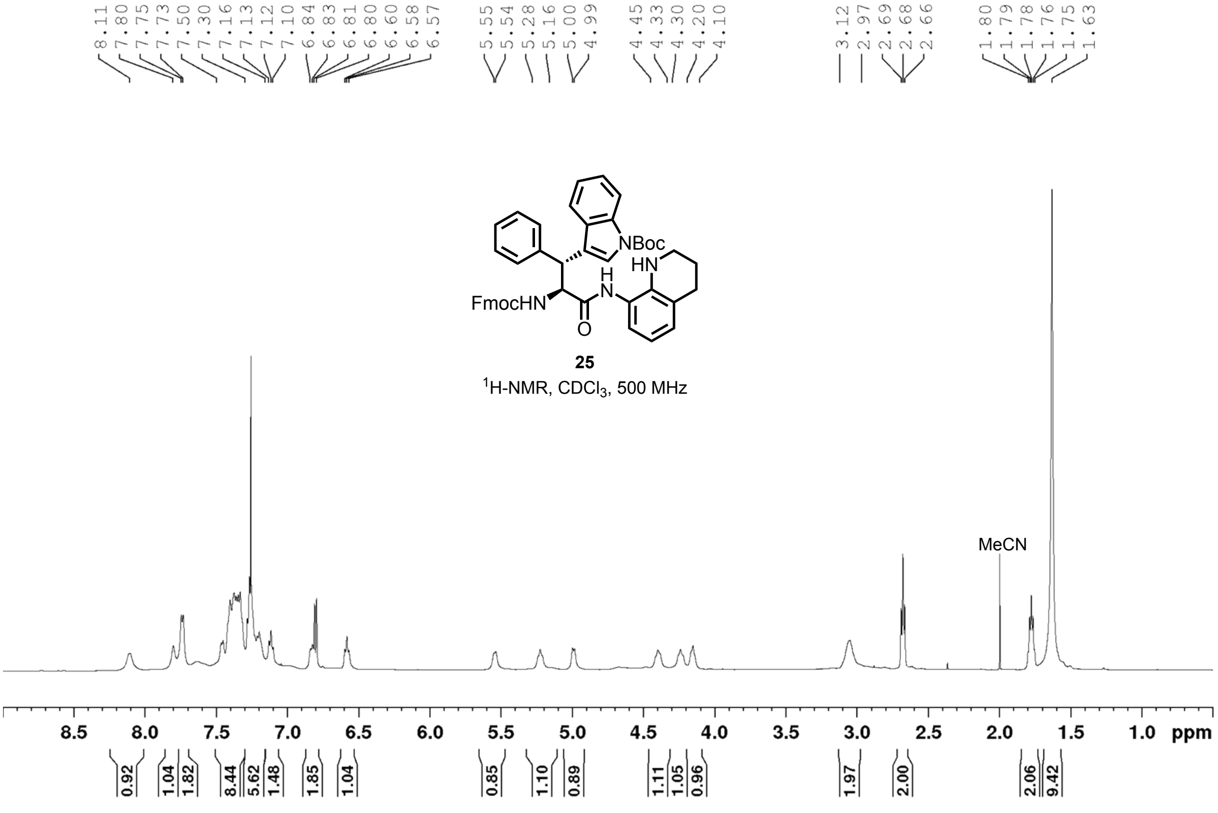

NBoc
HN
H
N
FmocHN
O
25
^{1}H-NMR, $CDCl_3$, 500 MHz
MeCN
ppm

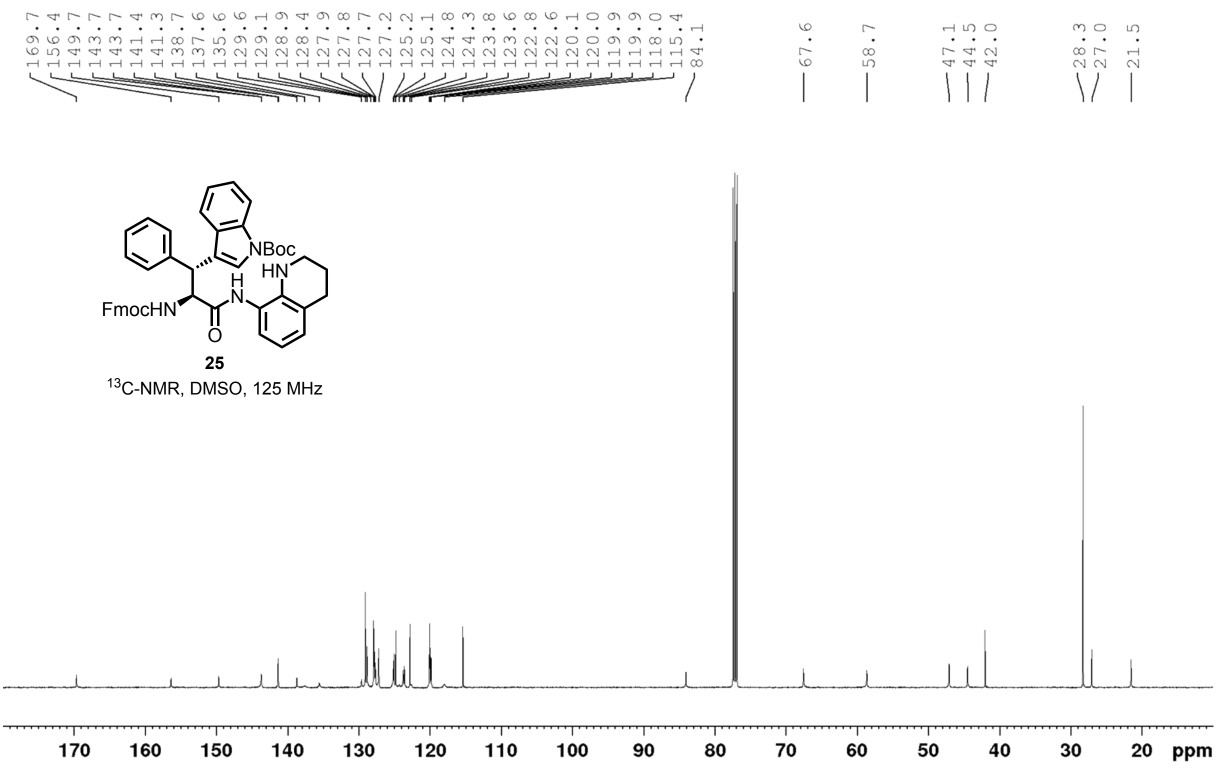

NBoc
HN
H
N
FmocHN
O
25
^{13}C-NMR, DMSO, 125 MHz
ppm

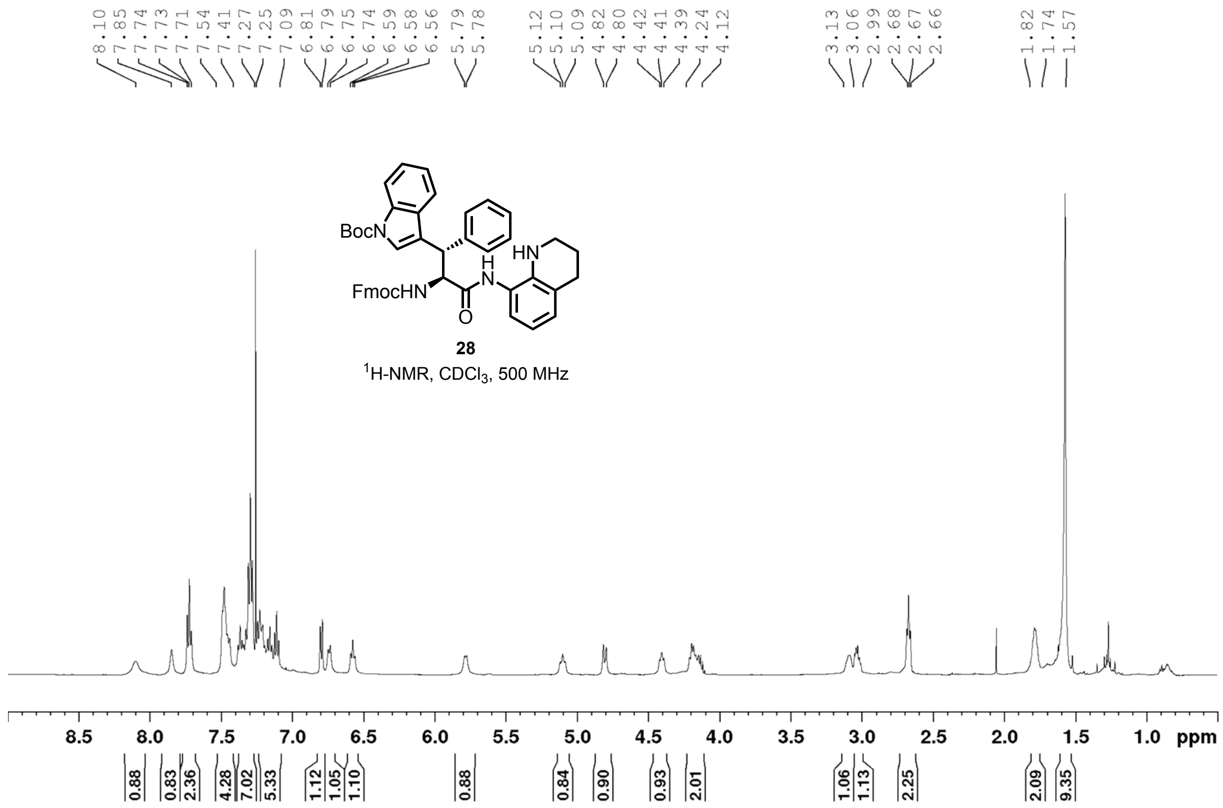
8.10 7.85 7.74 7.73 7.71 7.54 7.41 7.27 7.25 7.09 6.81 6.79 6.75 6.74 6.59 6.58 6.56 5.79 5.78 5.12 5.10 5.09 4.82 4.80 4.42 4.41 4.39 4.24 4.12 3.13 3.06 2.99 2.68 2.67 2.66 1.82 1.74 1.57
BocN
FmocHN
HN
H
N
O
28
^{1}H-NMR, $CDCl_3$, 500 MHz
8.5 8.0 7.5 7.0 6.5 6.0 5.5 5.0 4.5 4.0 3.5 3.0 2.5 2.0 1.5 1.0 ppm
0.88 0.83 2.36 4.28 7.02 5.33 1.12 1.05 1.10 0.88 0.84 0.90 0.93 2.01 1.06 1.13 2.25 2.09 9.35

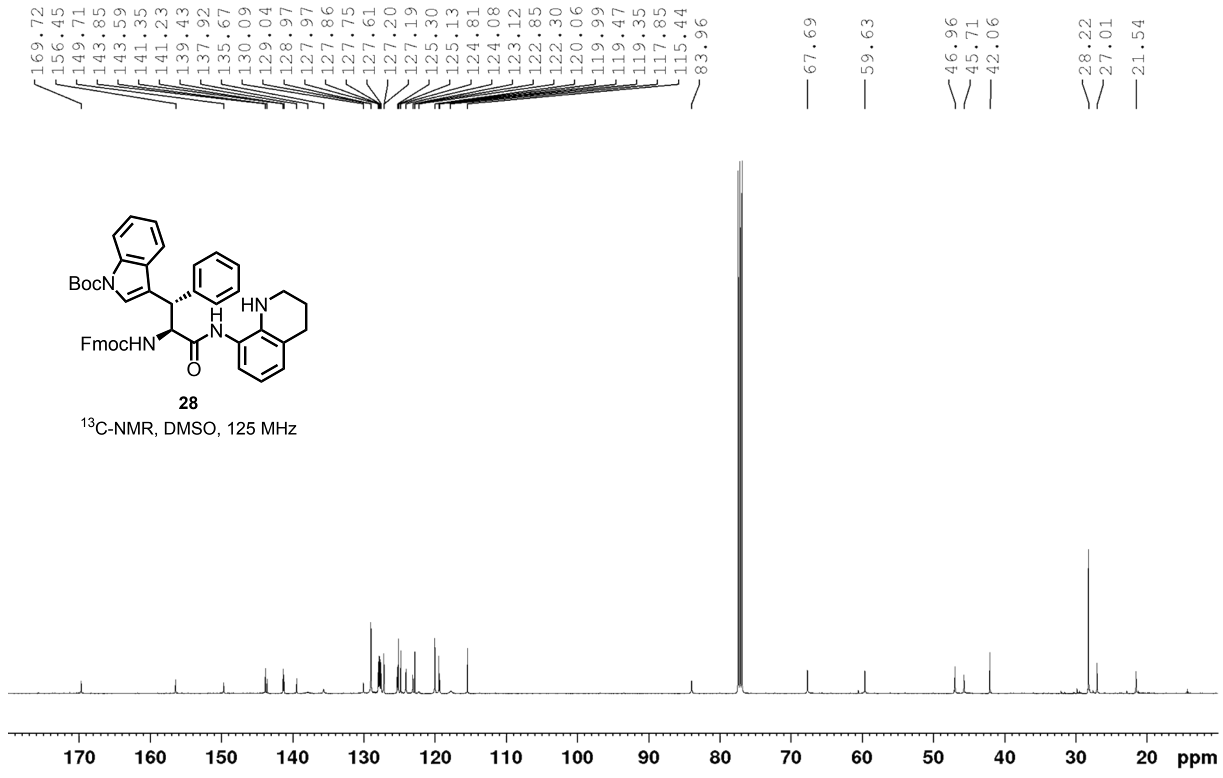
169.72 156.45 149.71 143.85 143.59 141.35 141.23 139.43 137.92 135.67 130.09 129.04 128.97 127.97 127.86 127.75 127.61 127.20 127.19 125.30 125.13 124.81 124.08 123.12 122.85 122.30 120.06 119.99 119.47 119.35 117.85 115.44 83.96 67.69 59.63 46.96 45.71 42.06 28.22 27.01 21.54
BocN
FmocHN
HN
H
N
O
28
^{13}C-NMR, DMSO, 125 MHz
170 160 150 140 130 120 110 100 90 80 70 60 50 40 30 20 ppm

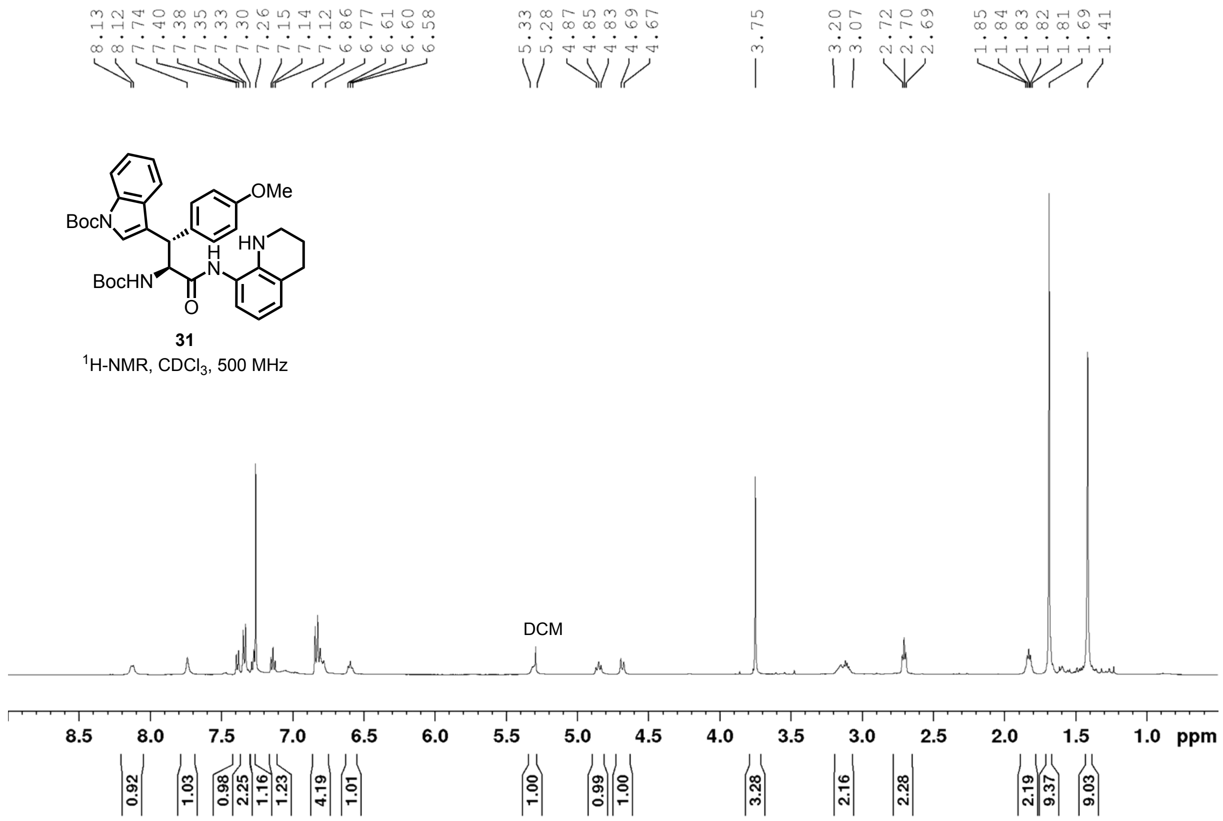
8.13 8.12 7.74 7.40 7.38 7.35 7.33 7.30 7.26 7.15 7.14 7.12 6.86 6.77 6.61 6.60 6.58
5.33 5.28 4.87 4.85 4.83 4.69 4.67
3.75
3.20 3.07 2.72 2.70 2.69
1.85 1.84 1.83 1.82 1.81 1.69 1.41
OMe
BocN
HN
H
N
BocHN
O
31
1H-NMR, CDCl3, 500 MHz
DCM
8.5 8.0 7.5 7.0 6.5 6.0 5.5 5.0 4.5 4.0 3.5 3.0 2.5 2.0 1.5 1.0 ppm
0.92 1.03 0.98 2.25 1.16 1.23 4.19 1.01 1.00 0.99 1.00 3.28 2.16 2.28 2.19 9.37 9.03

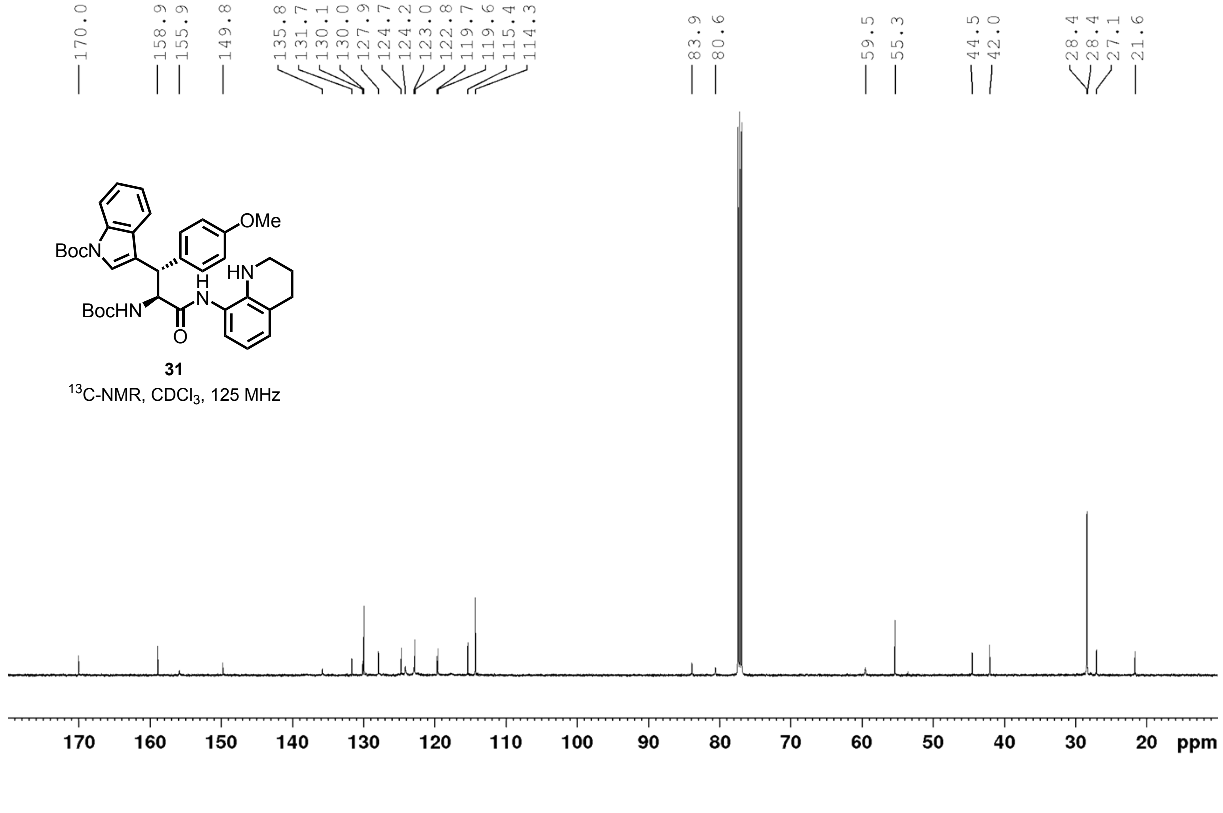
170.0
158.9 155.9
149.8
135.8 131.7 130.1 130.0 127.9 124.7 124.2 123.0 122.8 119.7 119.6 115.4 114.3
83.9 80.6
59.5 55.3
44.5 42.0
28.4 28.4 27.1 21.6
OMe
BocN
HN
H
N
BocHN
O
31
13C-NMR, CDCl3, 125 MHz
170 160 150 140 130 120 110 100 90 80 70 60 50 40 30 20 ppm

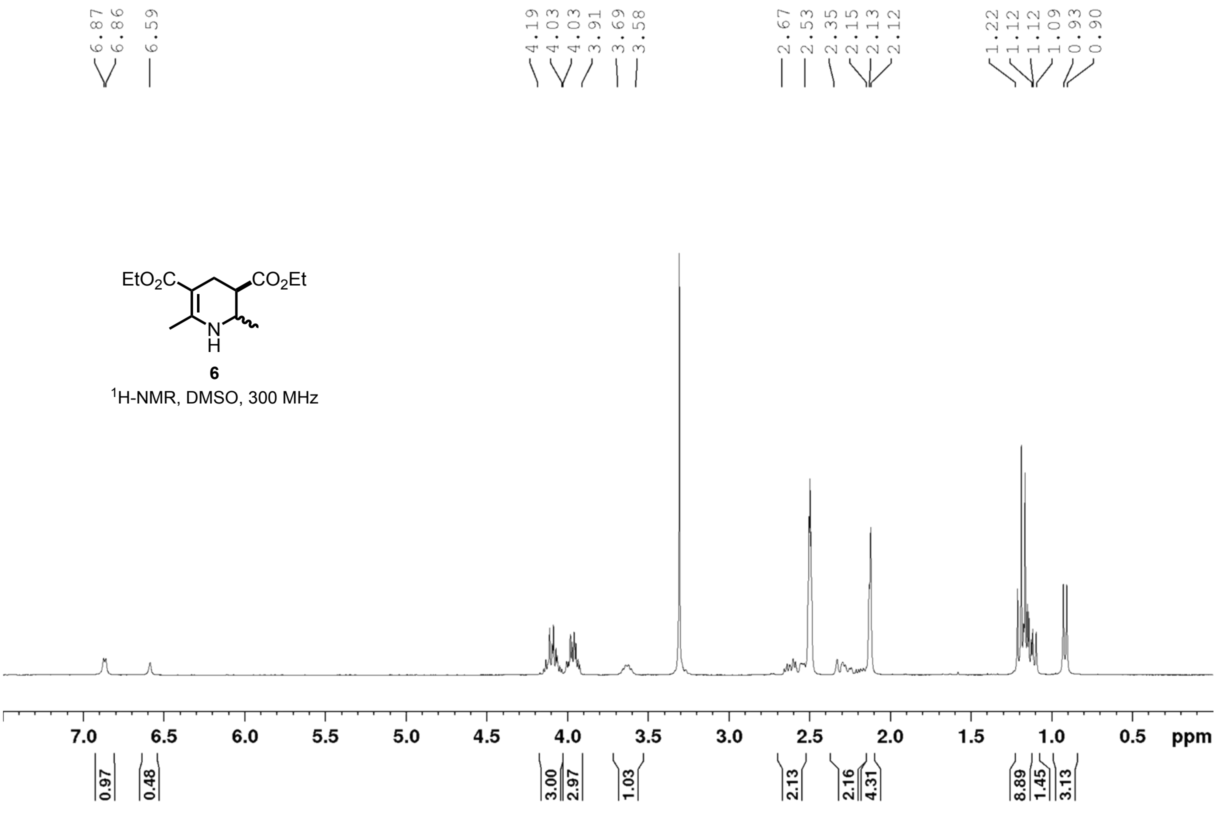
6.87
6.86
6.59
4.19
4.03
4.03
3.91
3.69
3.58
2.67
2.53
2.35
2.15
2.13
2.12
1.22
1.12
1.12
1.09
0.93
0.90
EtO_2C
CO_2Et
N
H
6
^{1}H-NMR, DMSO, 300 MHz
7.0
6.5
6.0
5.5
5.0
4.5
4.0
3.5
3.0
2.5
2.0
1.5
1.0
0.5
ppm
0.97
0.48
3.00
2.97
1.03
2.13
2.16
4.31
8.89
1.45
3.13

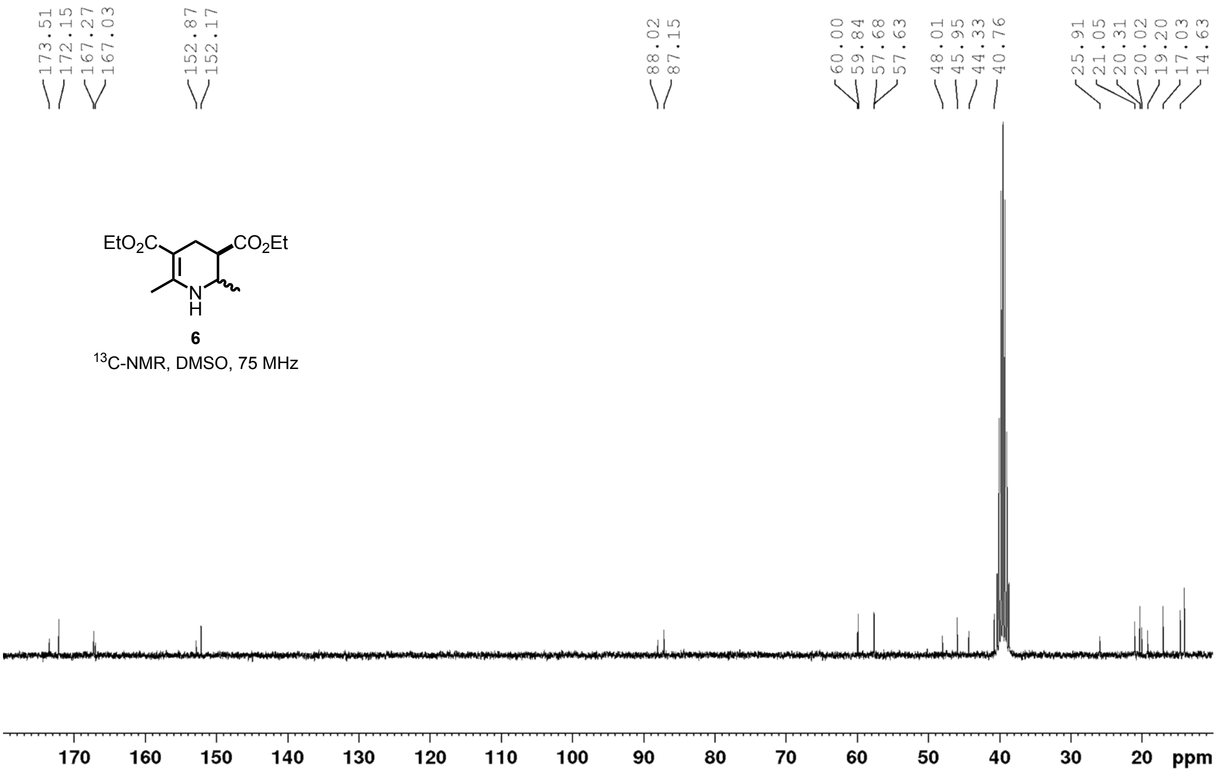
173.51
172.15
167.27
167.03
152.87
152.17
88.02
87.15
60.00
59.84
57.68
57.63
48.01
45.95
44.33
40.76
25.91
21.05
20.31
20.02
19.20
17.03
14.63
EtO_2C
CO_2Et
N
H
6
^{13}C-NMR, DMSO, 75 MHz
170
160
150
140
130
120
110
100
90
80
70
60
50
40
30
20
ppm

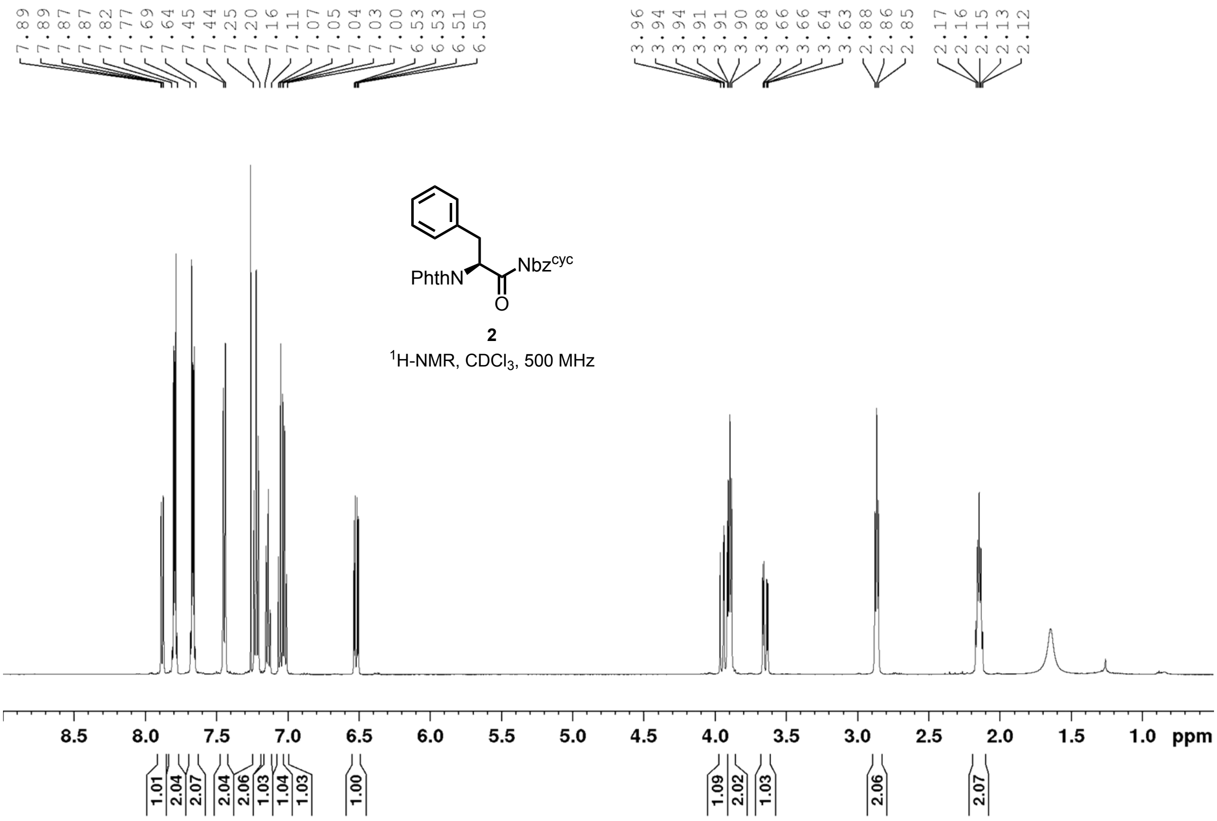
7.89 7.89 7.87 7.87 7.82 7.77 7.69 7.64 7.45 7.44 7.25 7.20 7.16 7.11 7.07 7.05 7.04 7.03 7.00 6.53 6.53 6.51 6.50
3.96 3.94 3.94 3.91 3.91 3.90 3.88 3.66 3.66 3.64 3.63 2.88 2.86 2.85 2.17 2.16 2.15 2.13 2.12
PhthN
Nbz^{cyc}
O
2
^{1}H-NMR, $CDCl_3$, 500 MHz
8.5 8.0 7.5 7.0 6.5 6.0 5.5 5.0 4.5 4.0 3.5 3.0 2.5 2.0 1.5 1.0 ppm
1.01 2.04 2.07 2.04 2.06 1.03 1.04 1.03 1.00 1.09 2.02 1.03 2.06 2.07

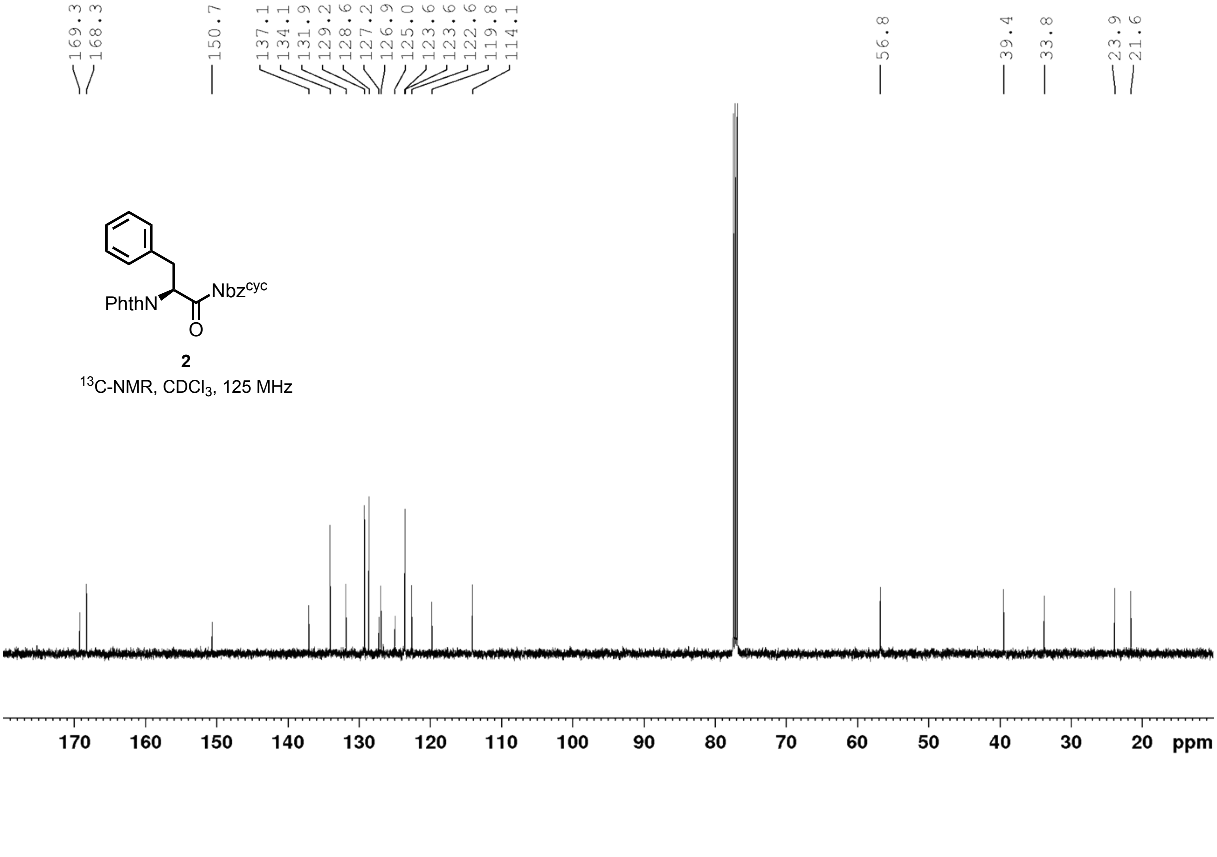
169.3 168.3 150.7 137.1 134.1 131.9 129.2 128.6 127.2 126.9 125.0 123.6 123.6 122.6 119.8 114.1 56.8 39.4 33.8 23.9 21.6
PhthN
Nbz^{cyc}
O
2
^{13}C-NMR, $CDCl_3$, 125 MHz
170 160 150 140 130 120 110 100 90 80 70 60 50 40 30 20 ppm

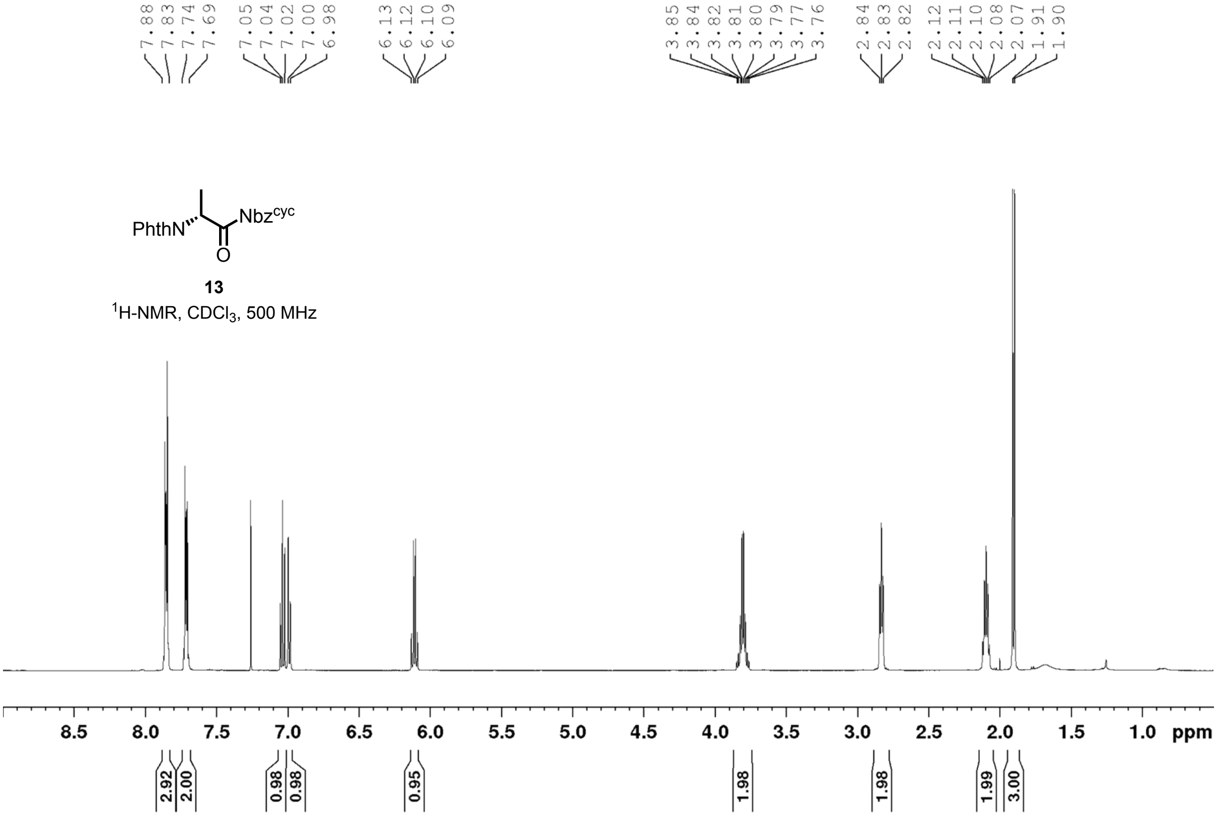
7.88
7.83
7.74
7.69
7.05
7.04
7.02
7.00
6.98
6.13
6.12
6.10
6.09
3.85
3.84
3.82
3.81
3.80
3.79
3.77
3.76
2.84
2.83
2.82
2.12
2.11
2.10
2.08
2.07
1.91
1.90
PhthN
Nbzcyc
O
13
1H-NMR, CDCl3, 500 MHz
8.5 8.0 7.5 7.0 6.5 6.0 5.5 5.0 4.5 4.0 3.5 3.0 2.5 2.0 1.5 1.0 ppm
2.92
2.00
0.98
0.98
0.95
1.98
1.98
1.99
3.00

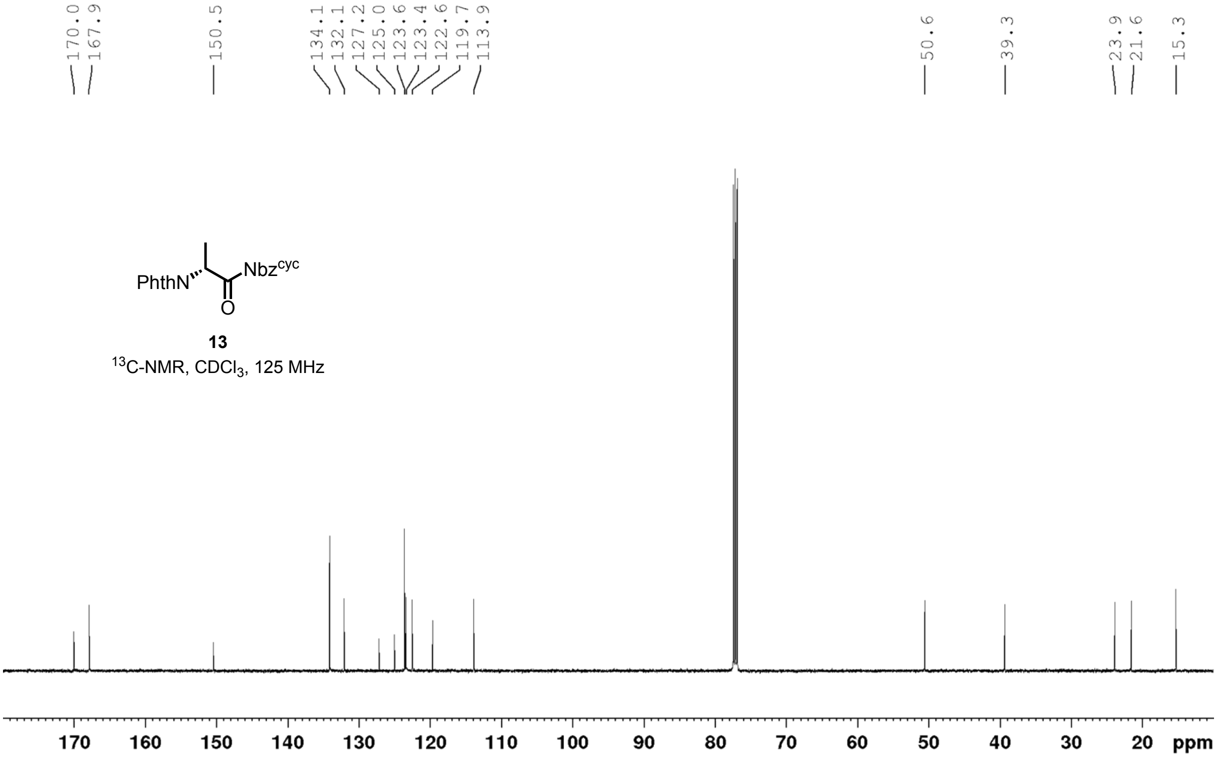
170.0
167.9
150.5
134.1
132.1
127.2
125.0
123.6
123.4
122.6
119.7
113.9
50.6
39.3
23.9
21.6
15.3
PhthN
Nbzcyc
O
13
13C-NMR, CDCl3, 125 MHz
170 160 150 140 130 120 110 100 90 80 70 60 50 40 30 20 ppm

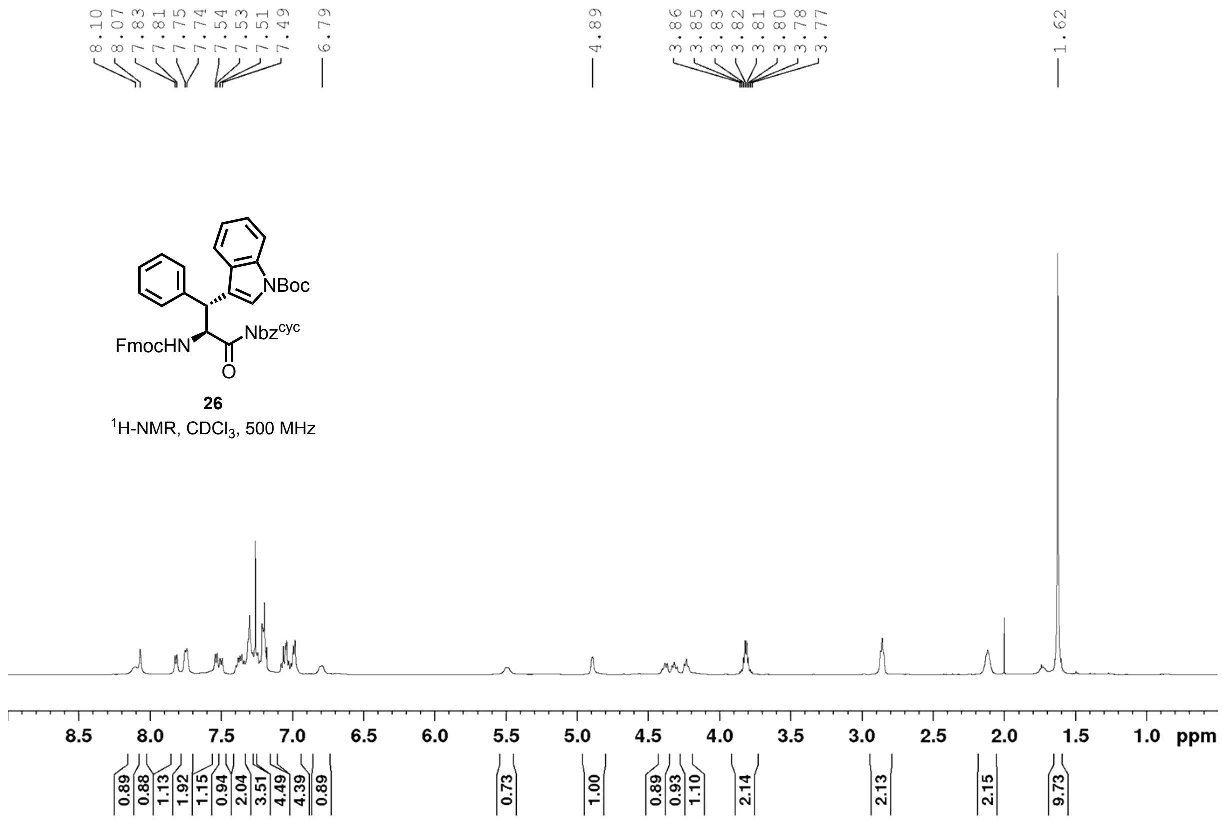
8.10 8.07 7.83 7.81 7.75 7.74 7.54 7.53 7.51 7.49 6.79
4.89
3.86 3.85 3.83 3.82 3.81 3.80 3.78 3.77
1.62
NBoc
FmocHN
Nbz^cyc
O
26
^{1}H-NMR, $CDCl_3$, 500 MHz
8.5 8.0 7.5 7.0 6.5 6.0 5.5 5.0 4.5 4.0 3.5 3.0 2.5 2.0 1.5 1.0 ppm
0.89 0.88 1.13 1.92 1.15 0.94 2.04 3.51 4.49 4.39 0.89 0.73 1.00 0.89 0.93 1.10 2.14 2.13 2.15 9.73

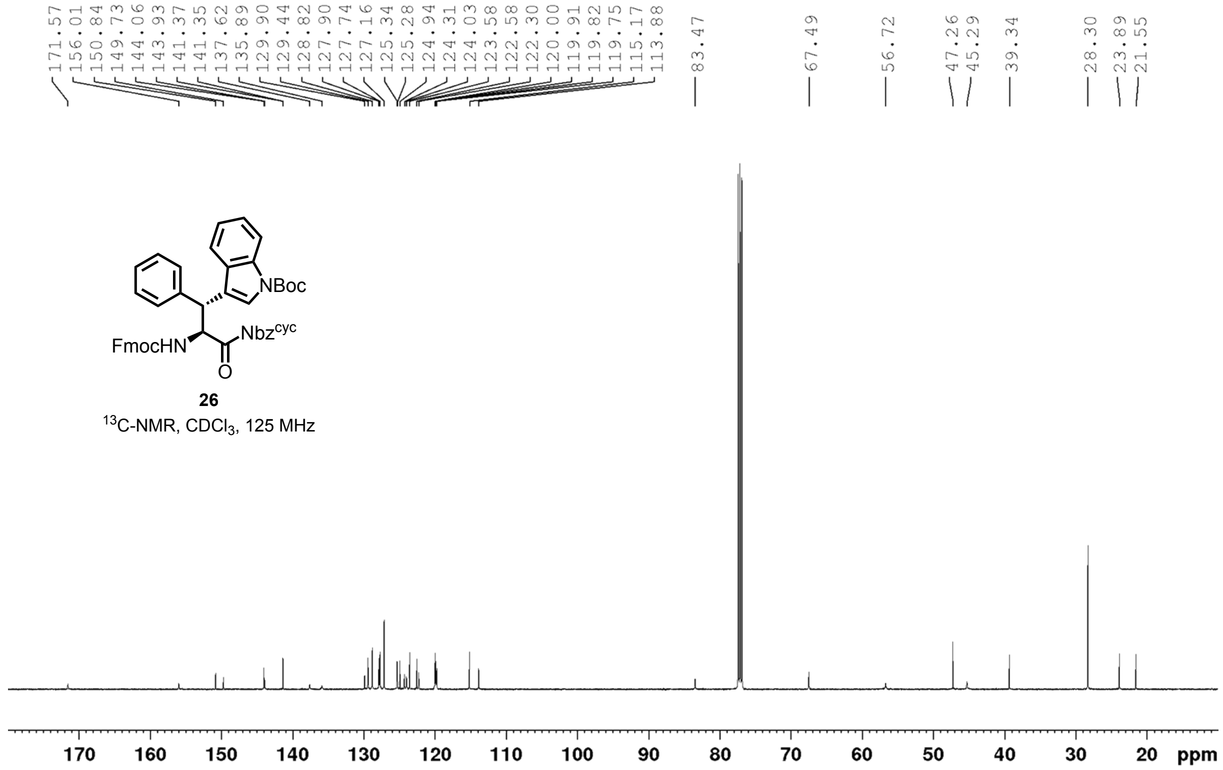
171.57 156.01 150.84 149.73 144.06 143.93 141.37 141.35 137.62 135.89 129.90 129.44 128.82 127.90 127.74 127.16 125.34 125.28 124.94 124.31 124.03 123.58 122.58 122.30 120.00 119.91 119.82 119.75 115.17 113.88
83.47
67.49
56.72
47.26 45.29
39.34
28.30 23.89 21.55
NBoc
FmocHN
Nbz^cyc
O
26
^{13}C-NMR, $CDCl_3$, 125 MHz
170 160 150 140 130 120 110 100 90 80 70 60 50 40 30 20 ppm

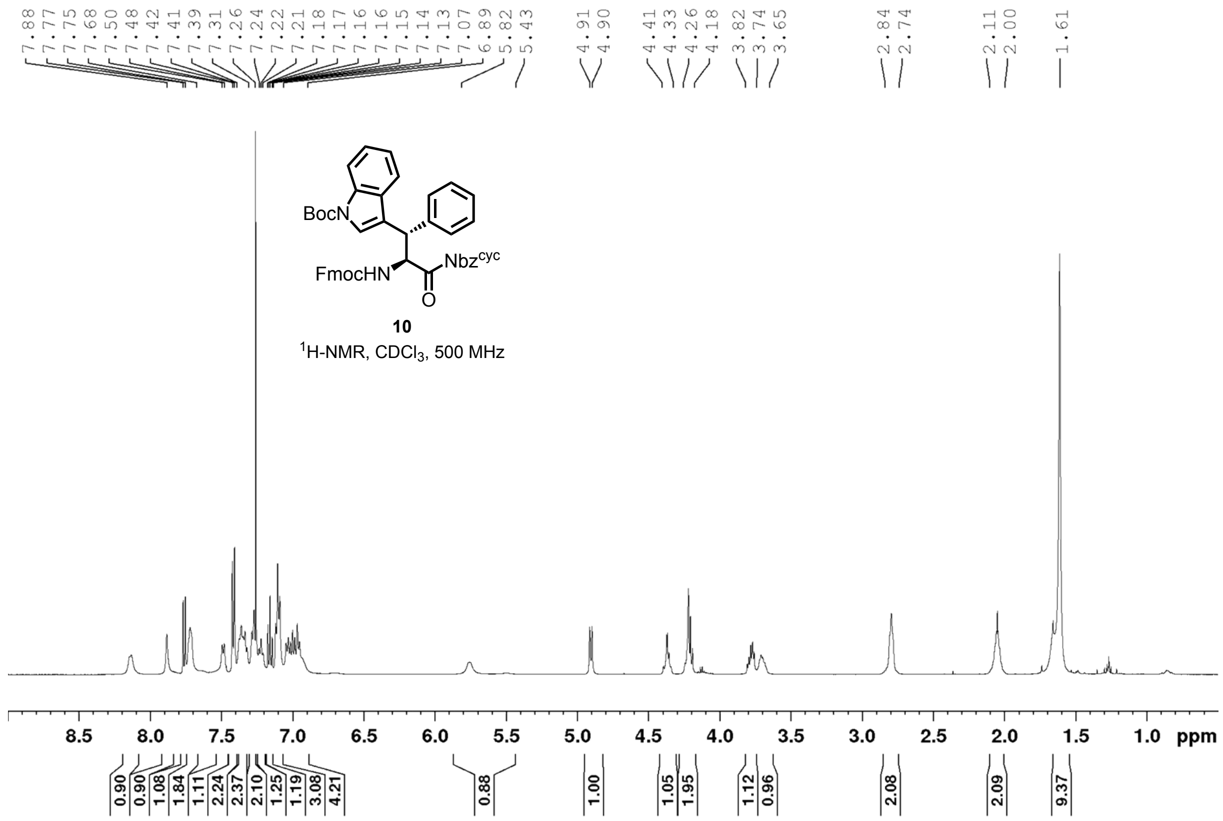
7.88 7.77 7.75 7.68 7.50 7.48 7.42 7.41 7.39 7.31 7.26 7.24 7.22 7.21 7.18 7.17 7.16 7.16 7.15 7.14 7.13 7.07 6.89 5.82 5.43
4.91 4.90 4.41 4.33 4.26 4.18 3.82 3.74 3.65 2.84 2.74 2.11 2.00 1.61
BocN
FmocHN
Nbz^cyc
O
10
1H-NMR, CDCl3, 500 MHz
8.5 8.0 7.5 7.0 6.5 6.0 5.5 5.0 4.5 4.0 3.5 3.0 2.5 2.0 1.5 1.0 ppm
0.90 0.90 1.08 1.84 1.11 2.24 2.37 2.10 1.25 1.19 3.08 4.21 0.88 1.00 1.05 1.95 1.12 0.96 2.08 2.09 9.37

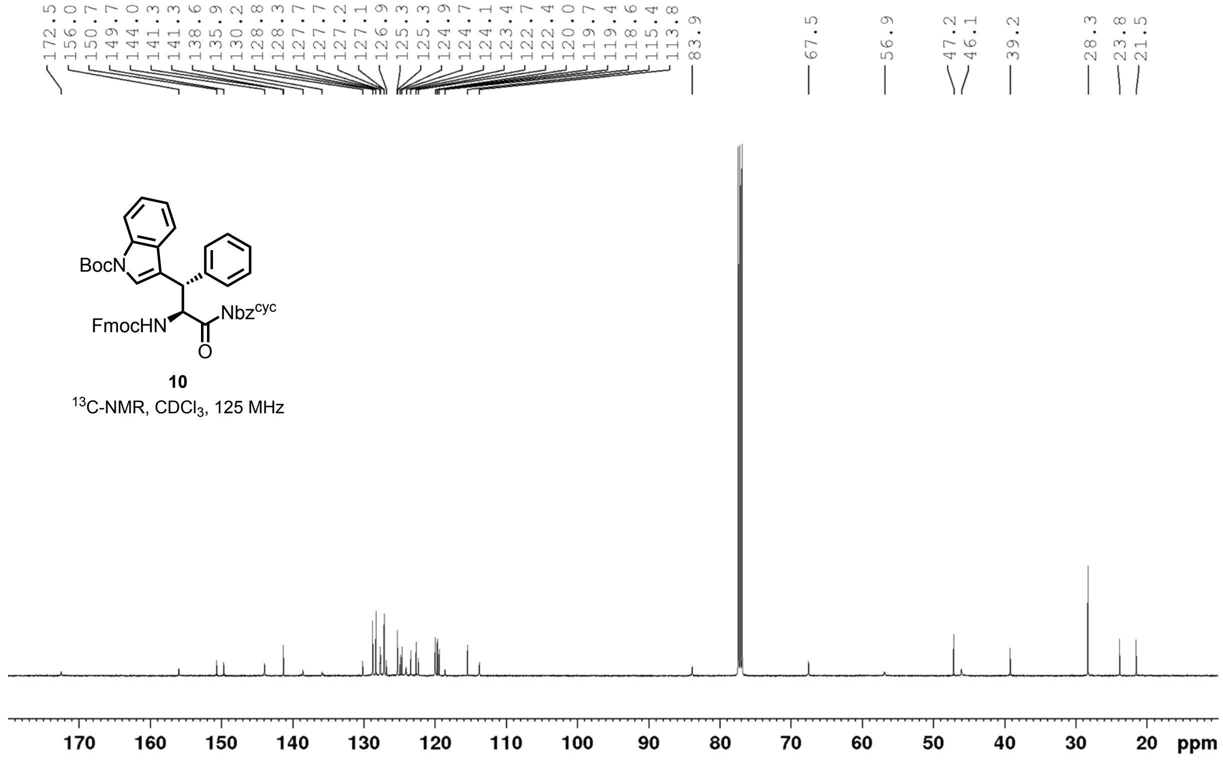
172.5 156.0 150.7 149.7 144.0 141.3 141.3 138.6 135.9 130.2 128.8 128.3 127.7 127.2 127.1 126.9 125.3 125.3 124.9 124.7 124.1 123.4 122.7 122.4 120.0 119.7 119.4 118.6 115.4 113.8 83.9
67.5 56.9 47.2 46.1 39.2 28.3 23.8 21.5
BocN
FmocHN
Nbz^cyc
O
10
13C-NMR, CDCl3, 125 MHz
170 160 150 140 130 120 110 100 90 80 70 60 50 40 30 20 ppm

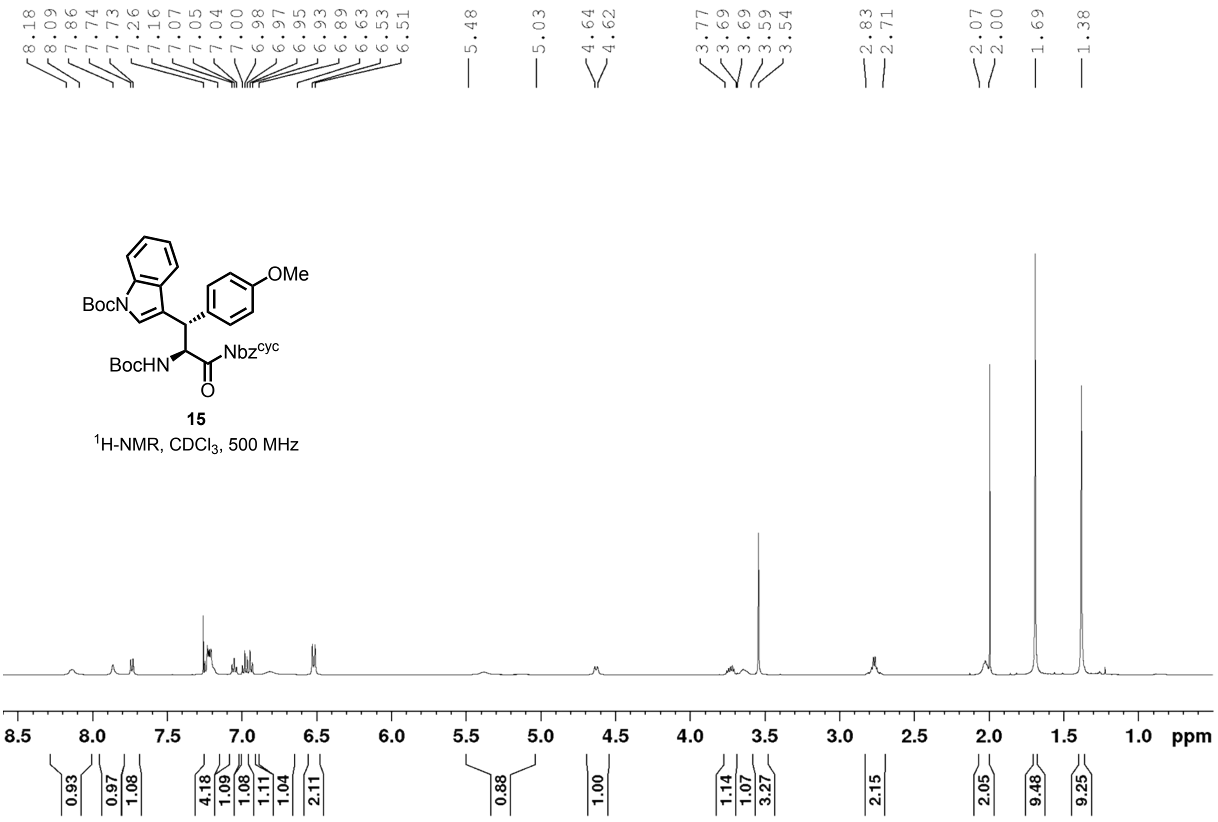
8.18 8.09 7.86 7.74 7.73 7.26 7.16 7.07 7.05 7.04 7.00 6.98 6.97 6.95 6.93 6.89 6.63 6.53 6.51 5.48 5.03 4.64 4.62 3.77 3.69 3.69 3.59 3.54 2.83 2.71 2.07 2.00 1.69 1.38
OMe
BocN
BocHN
Nbz^cyc
O
15
1H-NMR, CDCl3, 500 MHz
8.5 8.0 7.5 7.0 6.5 6.0 5.5 5.0 4.5 4.0 3.5 3.0 2.5 2.0 1.5 1.0 ppm
0.93 0.97 1.08 4.18 1.09 1.08 1.11 1.04 2.11 0.88 1.00 1.14 1.07 3.27 2.15 2.05 9.48 9.25

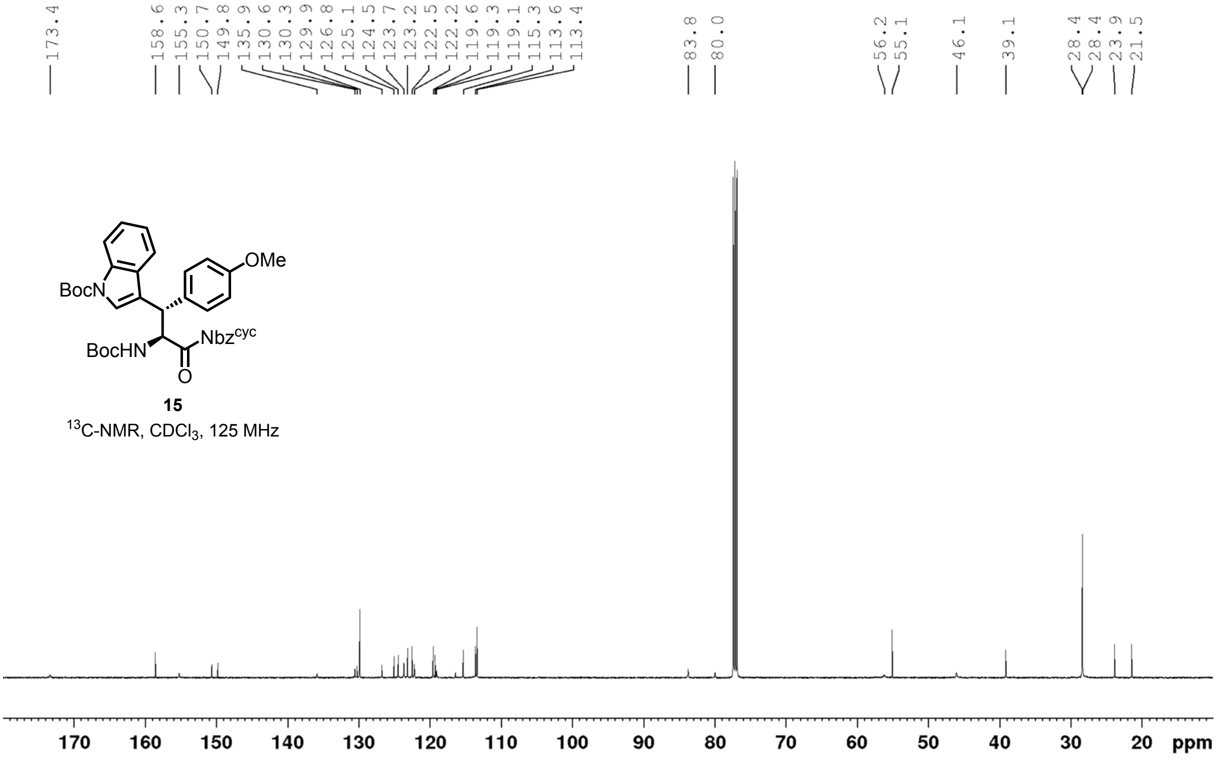
173.4 158.6 155.3 150.7 149.8 135.9 130.6 130.3 129.9 126.8 125.1 124.5 123.7 123.2 122.5 122.2 119.6 119.3 119.1 115.3 113.6 113.4 83.8 80.0 56.2 55.1 46.1 39.1 28.4 28.4 23.9 21.5
OMe
BocN
BocHN
Nbz^cyc
O
15
13C-NMR, CDCl3, 125 MHz
170 160 150 140 130 120 110 100 90 80 70 60 50 40 30 20 ppm

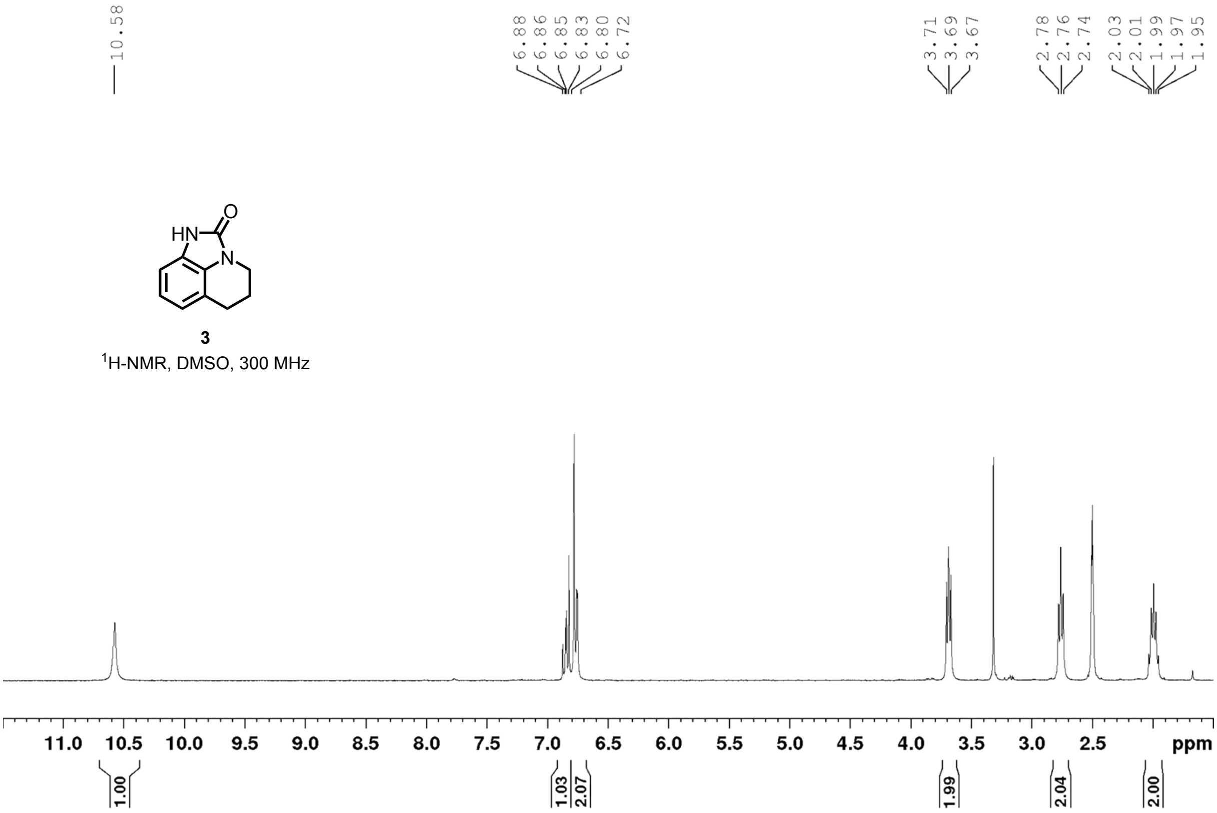

3

^{1}H-NMR, DMSO, 300 MHz

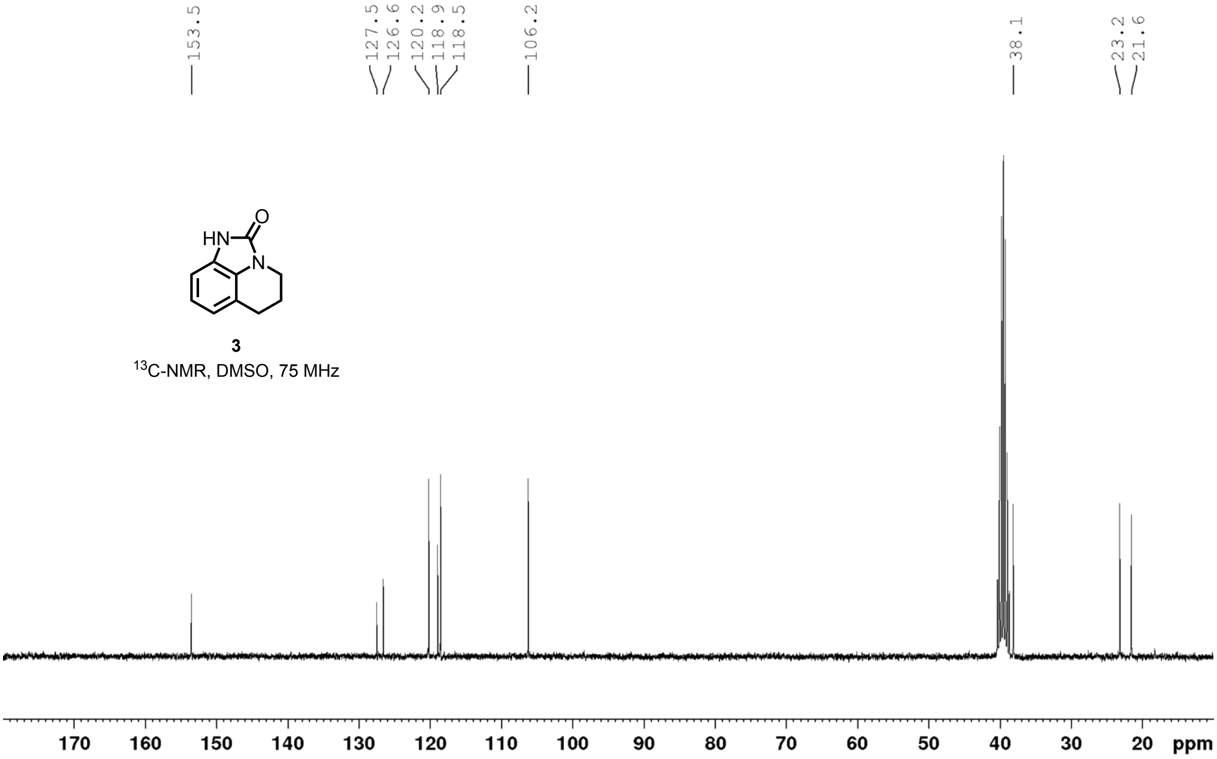

3

^{13}C-NMR, DMSO, 75 MHz

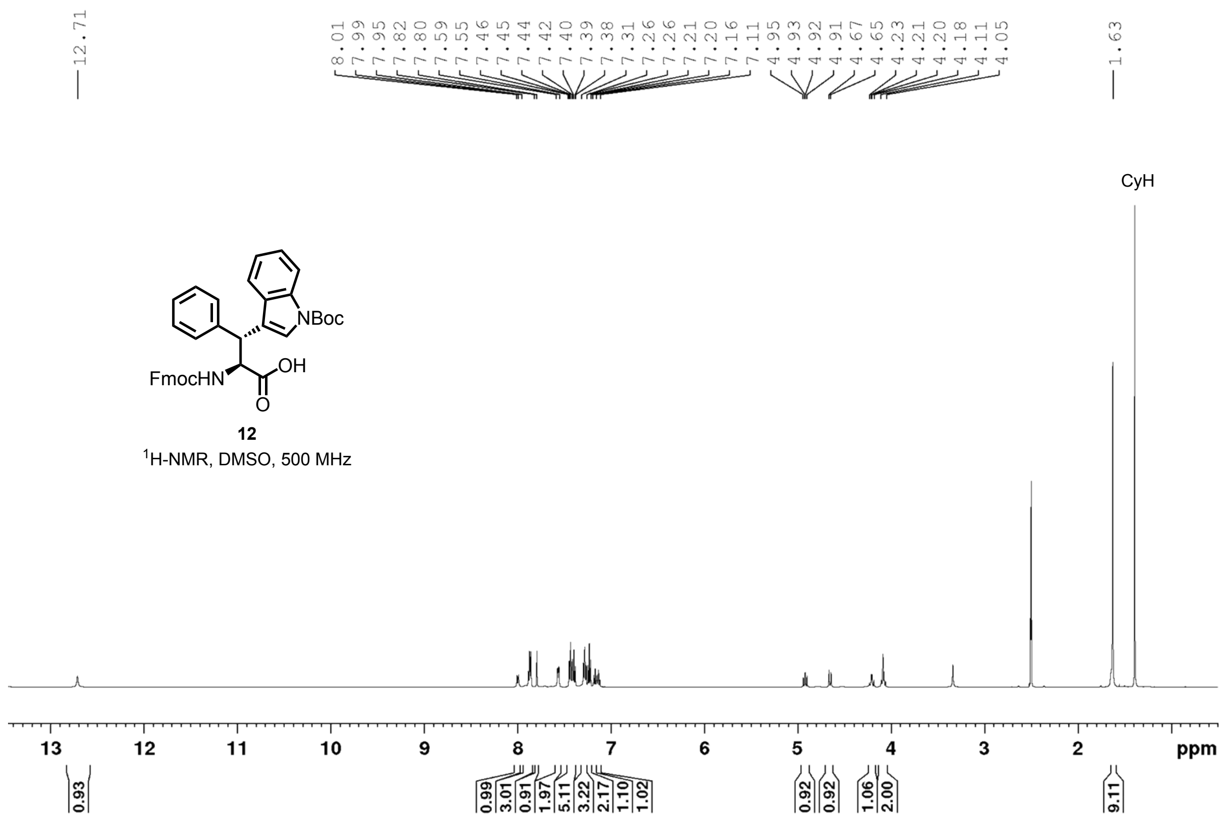

12.71
8.01 7.99 7.95 7.82 7.80 7.59 7.55 7.46 7.45 7.44 7.42 7.40 7.39 7.38 7.31 7.26 7.26 7.21 7.20 7.16 7.11
4.95 4.93 4.92 4.91 4.67 4.65 4.23 4.21 4.20 4.18 4.11 4.05
1.63
CyH
NBoc
OH
FmocHN
O
12
1H-NMR, DMSO, 500 MHz
13 12 11 10 9 8 7 6 5 4 3 2 ppm
0.93
0.99 3.01 0.91 1.97 5.11 3.22 2.17 1.10 1.02
0.92 0.92
1.06 2.00
9.11

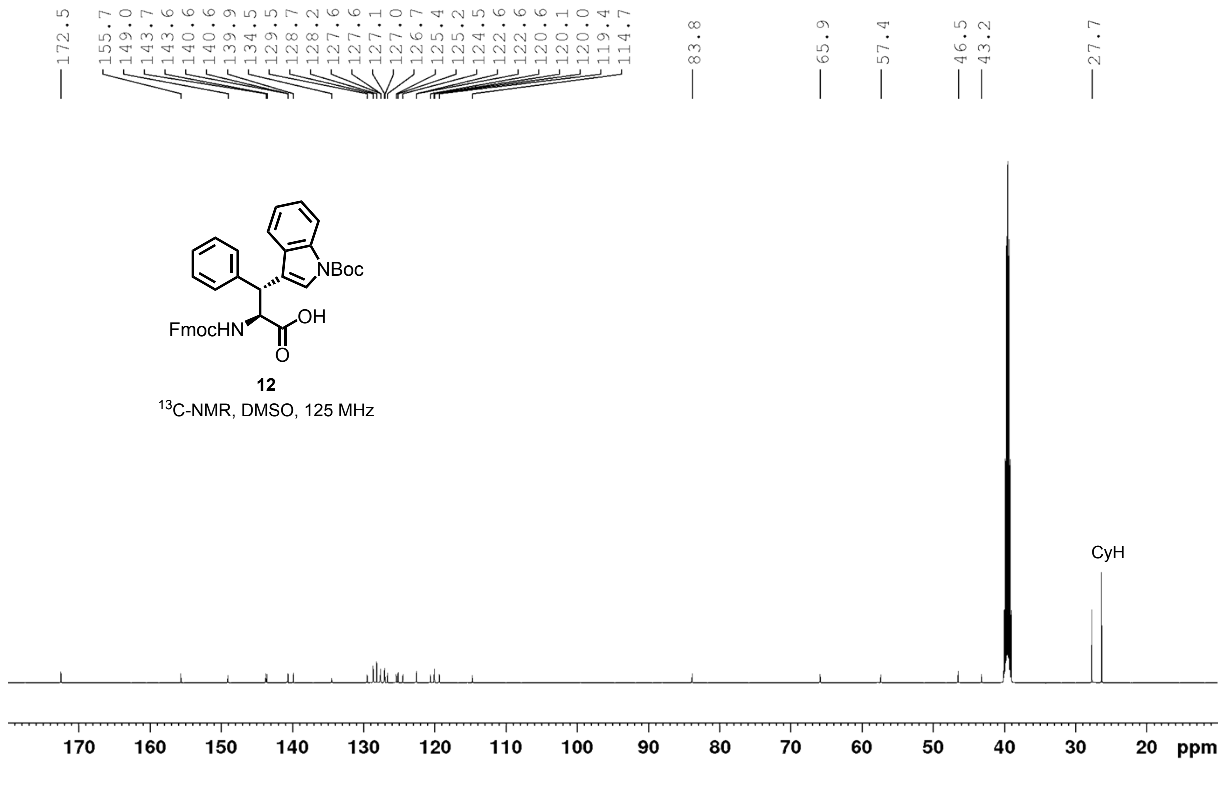

172.5
155.7 149.0 143.7 143.6 140.6 140.6 139.9 134.5 129.5 128.7 128.2 127.6 127.6 127.1 127.0 126.7 125.4 125.2 124.5 122.6 122.6 120.6 120.1 120.0 119.4 114.7
83.8
65.9
57.4
46.5
43.2
27.7
NBoc
OH
FmocHN
O
12
13C-NMR, DMSO, 125 MHz
CyH
170 160 150 140 130 120 110 100 90 80 70 60 50 40 30 20 ppm

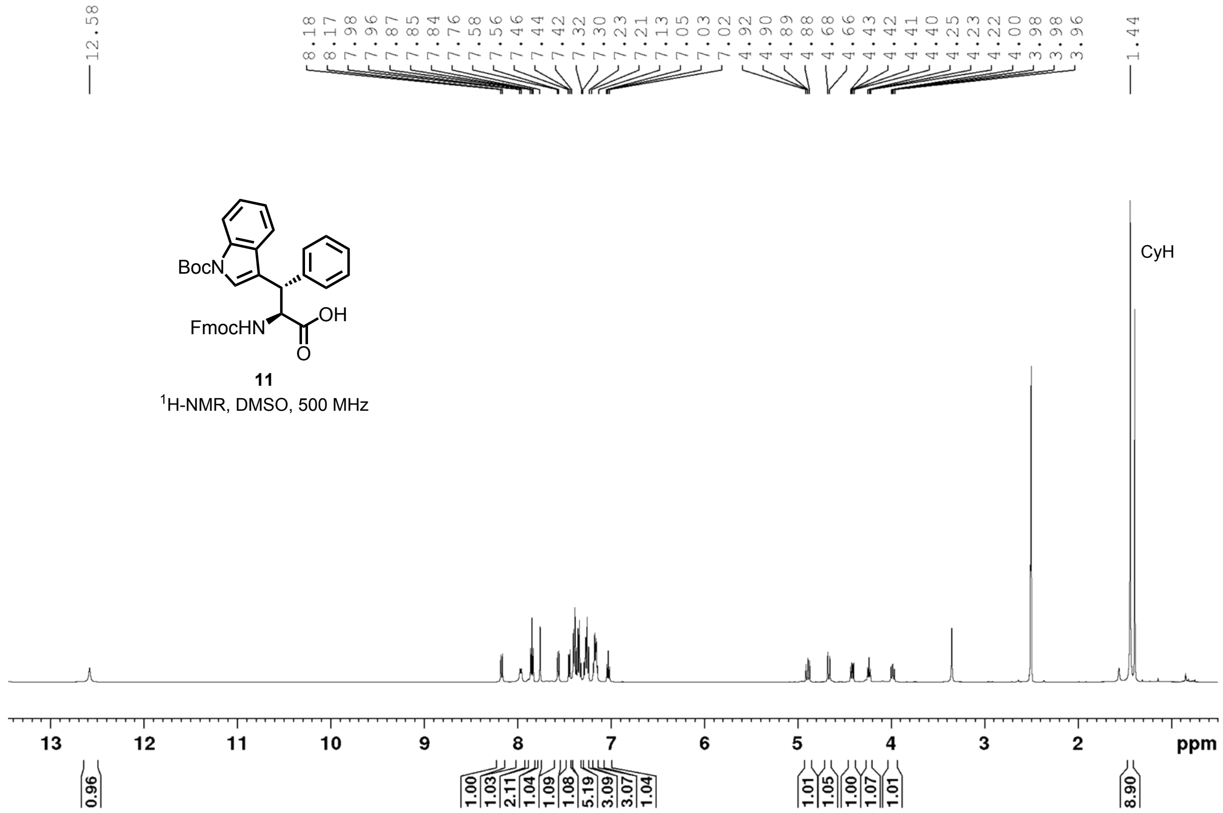
12.58
8.18 8.17 7.98 7.96 7.87 7.85 7.84 7.76 7.58 7.56 7.46 7.44 7.42 7.32 7.30 7.23 7.21 7.13 7.05 7.03 7.02
4.92 4.90 4.89 4.88 4.68 4.66 4.43 4.42 4.41 4.40 4.25 4.23 4.22 4.00 3.98 3.98 3.96
1.44
BocN
FmocHN
OH
O
11
1H-NMR, DMSO, 500 MHz
CyH
13 12 11 10 9 8 7 6 5 4 3 2 ppm
0.96
1.00 1.03 2.11 1.04 1.09 1.08 5.19 3.09 3.07 1.04
1.01 1.05 1.00 1.07 1.01
8.90

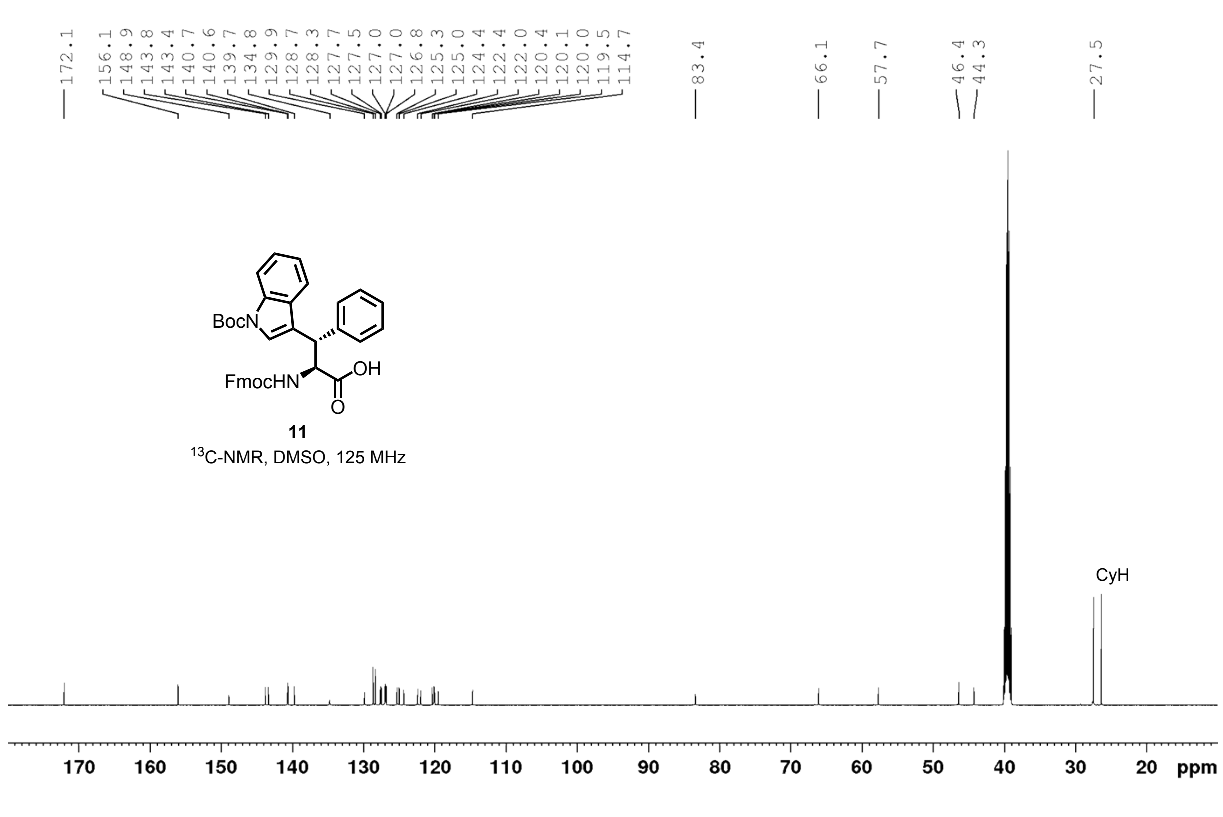
172.1
156.1 148.9 143.8 143.4 140.7 140.6 139.7 134.8 129.9 128.7 128.3 127.7 127.5 127.0 127.0 126.8 125.3 125.0 124.4 122.4 122.0 120.4 120.1 120.0 119.5 114.7
83.4
66.1
57.7
46.4 44.3
27.5
BocN
FmocHN
OH
O
11
13C-NMR, DMSO, 125 MHz
CyH
170 160 150 140 130 120 110 100 90 80 70 60 50 40 30 20 ppm

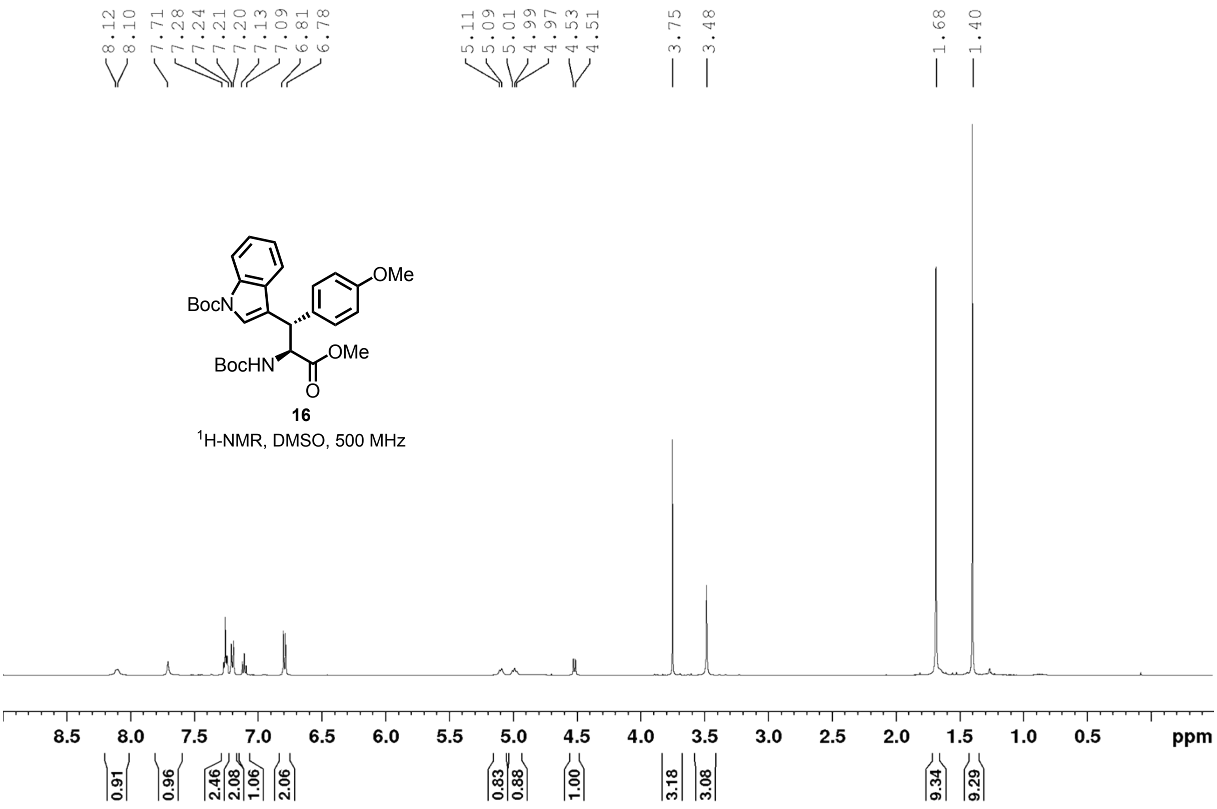

16

^{1}H-NMR, DMSO, 500 MHz

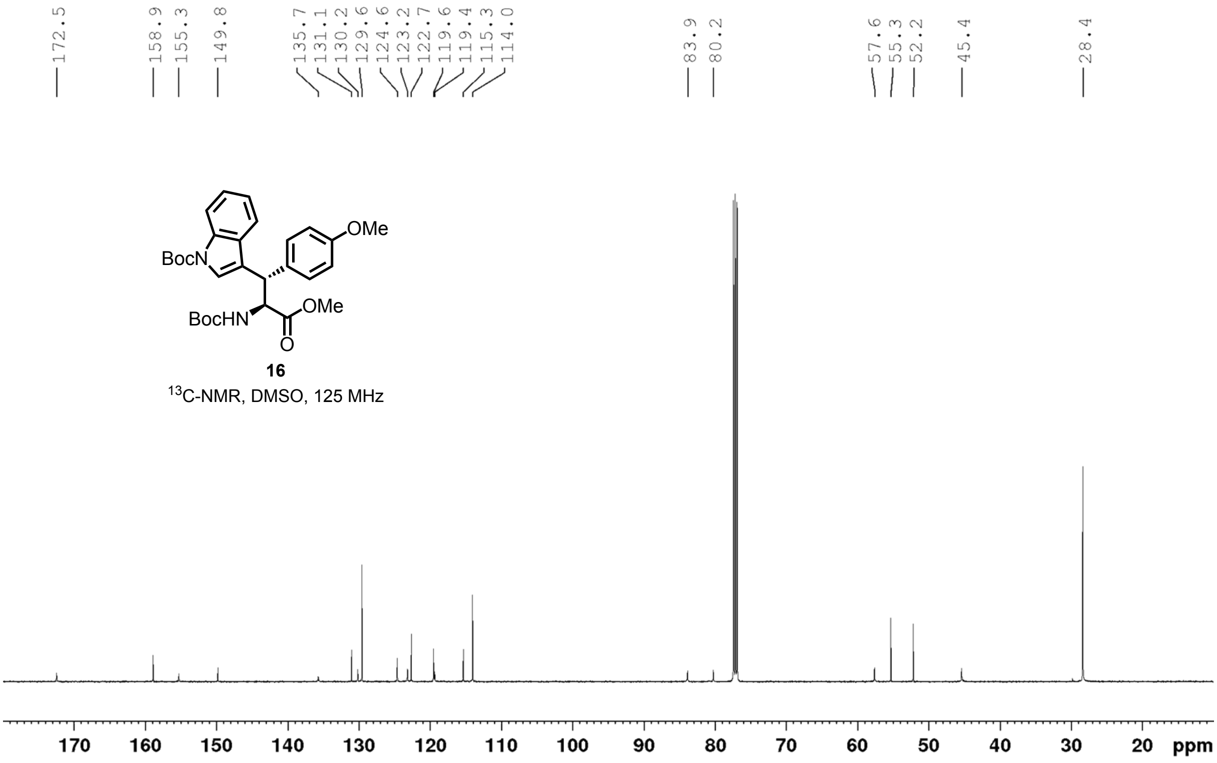

16

^{13}C-NMR, DMSO, 125 MHz

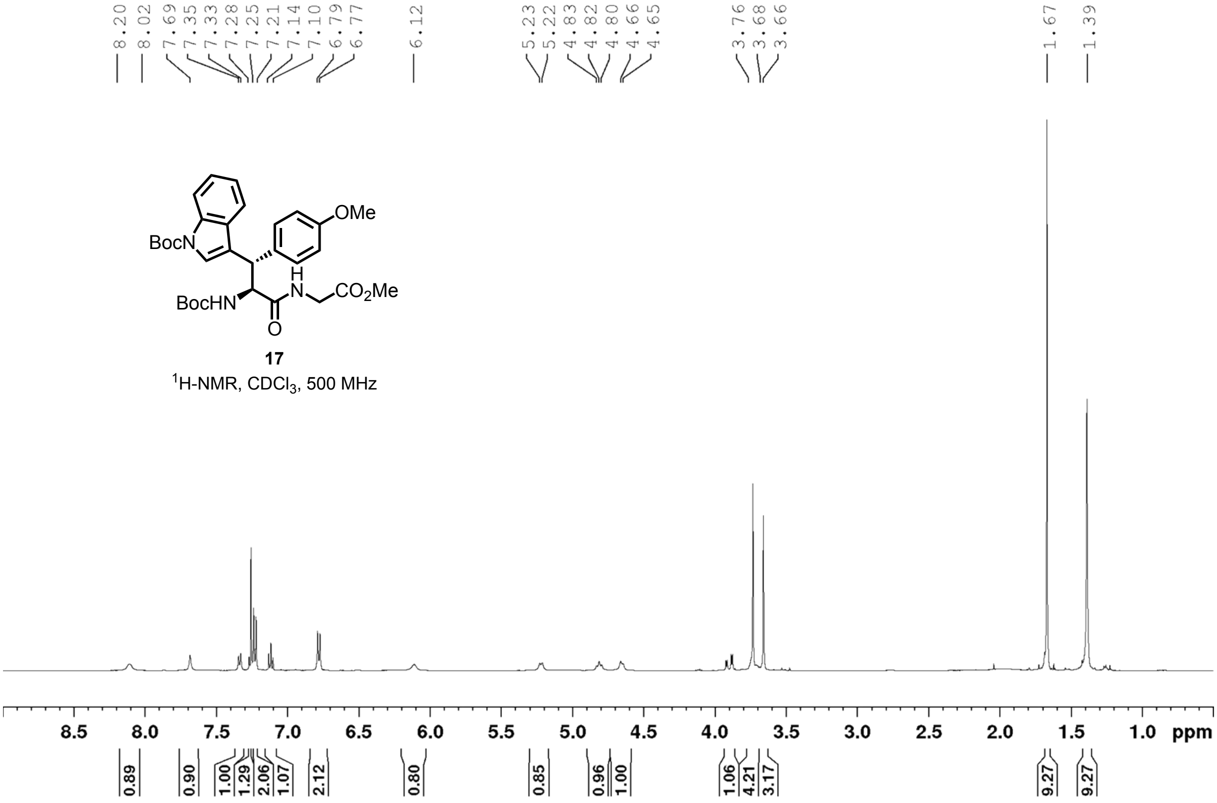

17
^{1}H-NMR, $CDCl_3$, 500 MHz

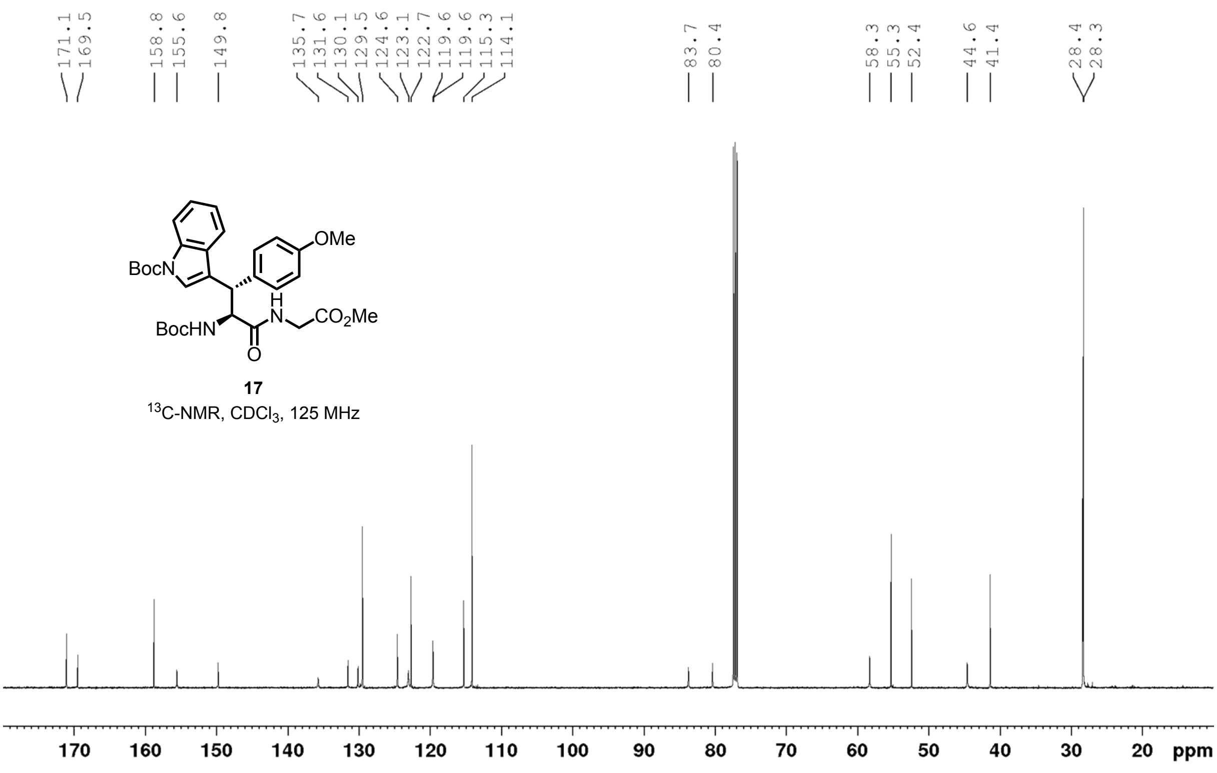

17
^{13}C-NMR, $CDCl_3$, 125 MHz

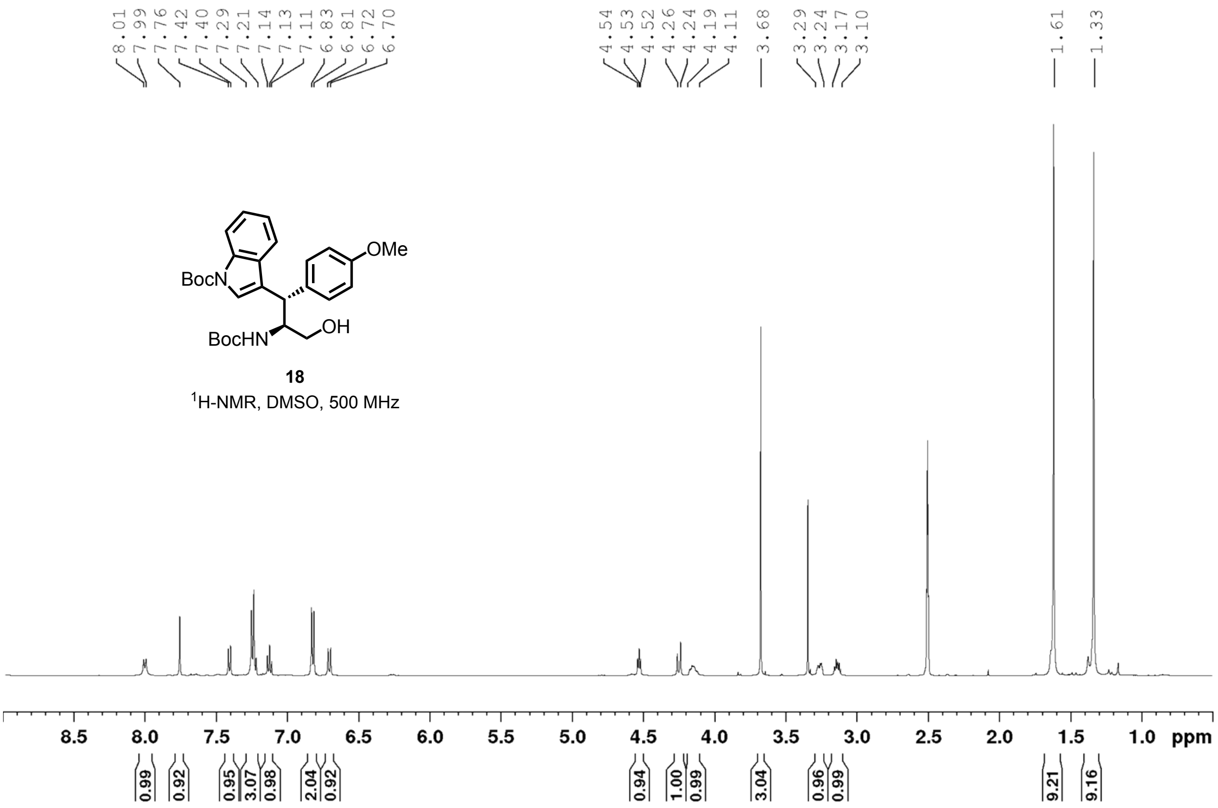

18

^{1}H-NMR, DMSO, 500 MHz

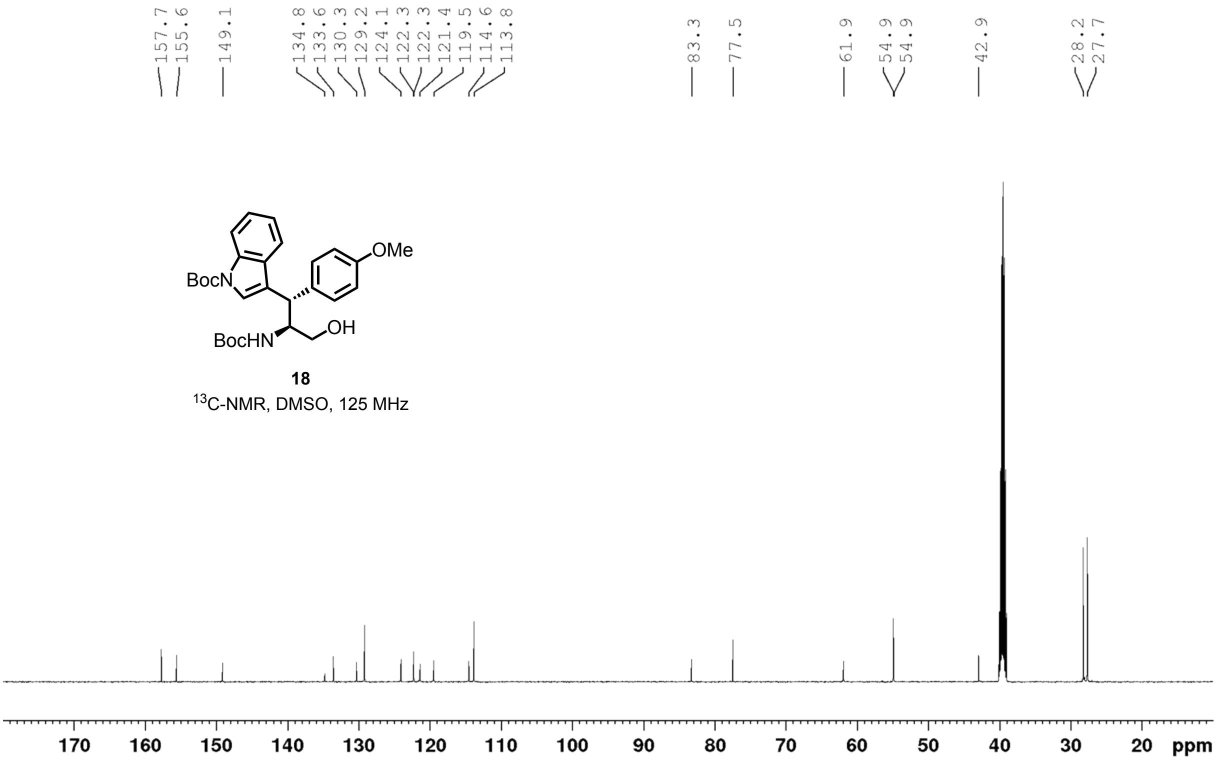

18

^{13}C-NMR, DMSO, 125 MHz

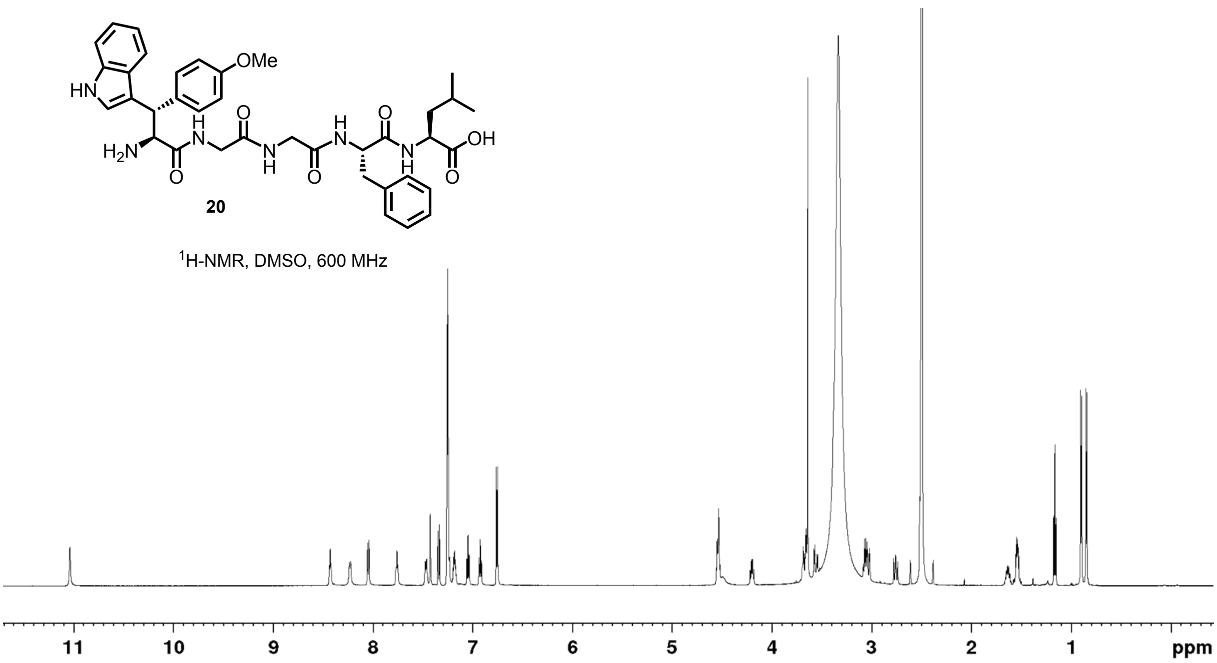
OMe
HN
H
N
O
O
OH
H2N
N
H
20
1H-NMR, DMSO, 600 MHz
11
10
9
8
7
6
5
4
3
2
1
ppm

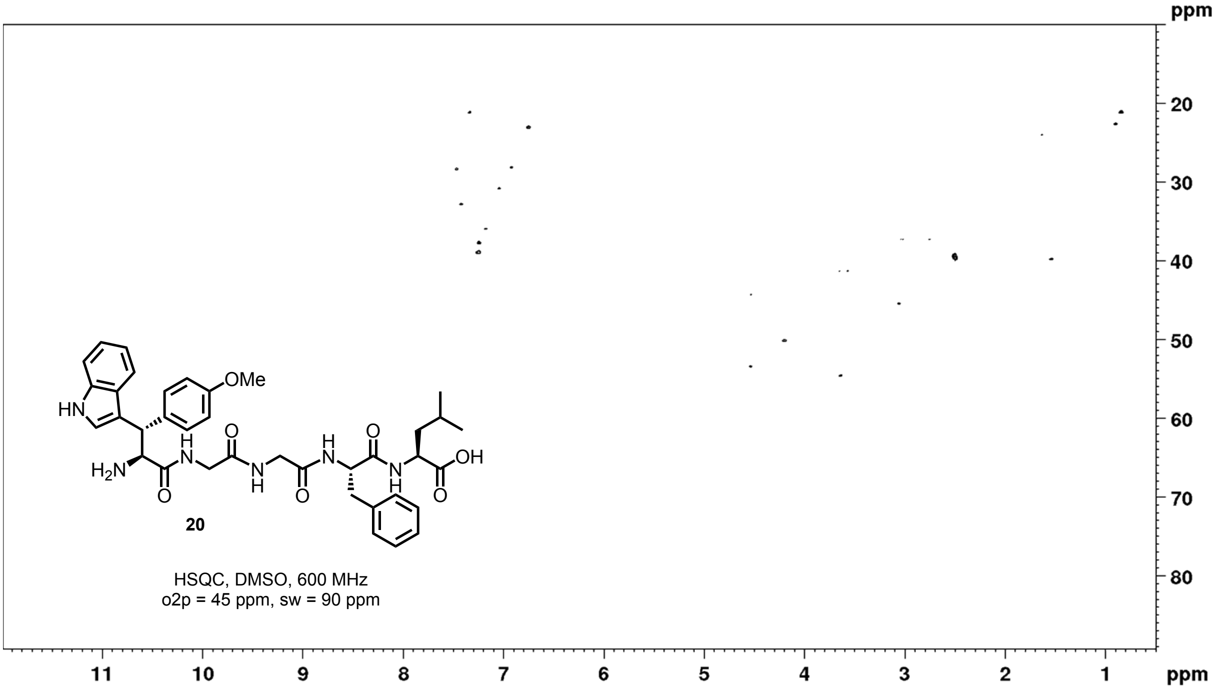
OMe
HN
H2N
OH
20
HSQC, DMSO, 600 MHz
o2p = 45 ppm, sw = 90 ppm
ppm
20
30
40
50
60
70
80
11
10
9
8
7
6
5
4
3
2
1

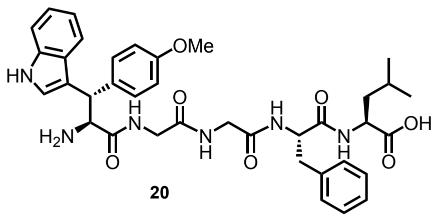

20

TOCSY, DMSO, 600 MHz

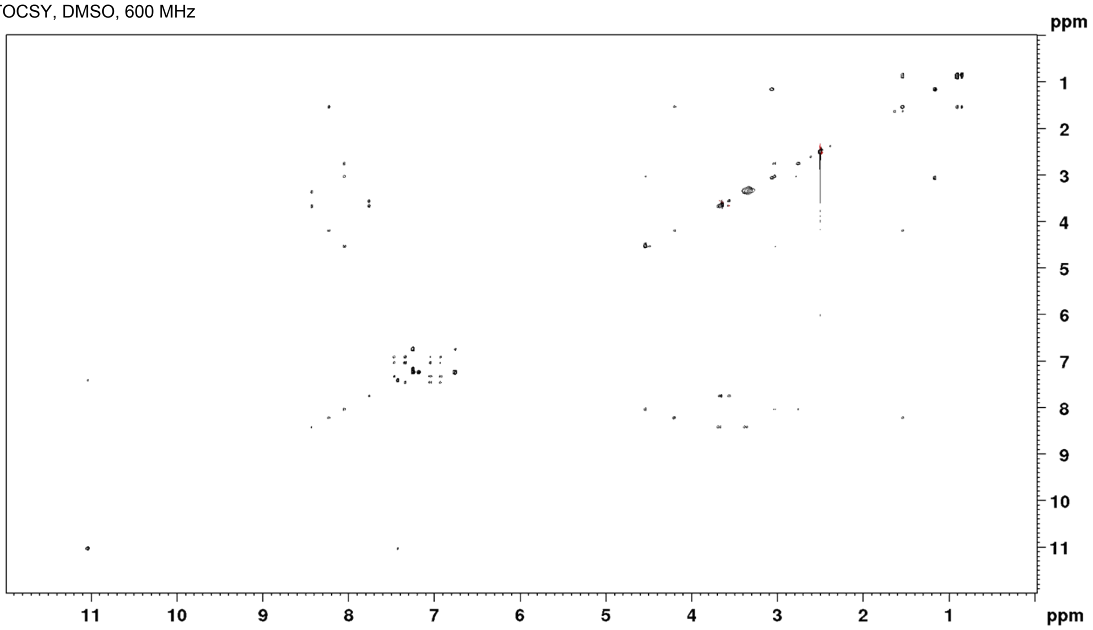

NLYIQ XLKDG GPSSG RPPPS

21

20mer Trp cage, X = Wrf

^{1}H-NMR, 280 K, 50 mM phosphate buffer pH 7.4/TFE-d_2/D_2O 60/30/10, 600 MHz

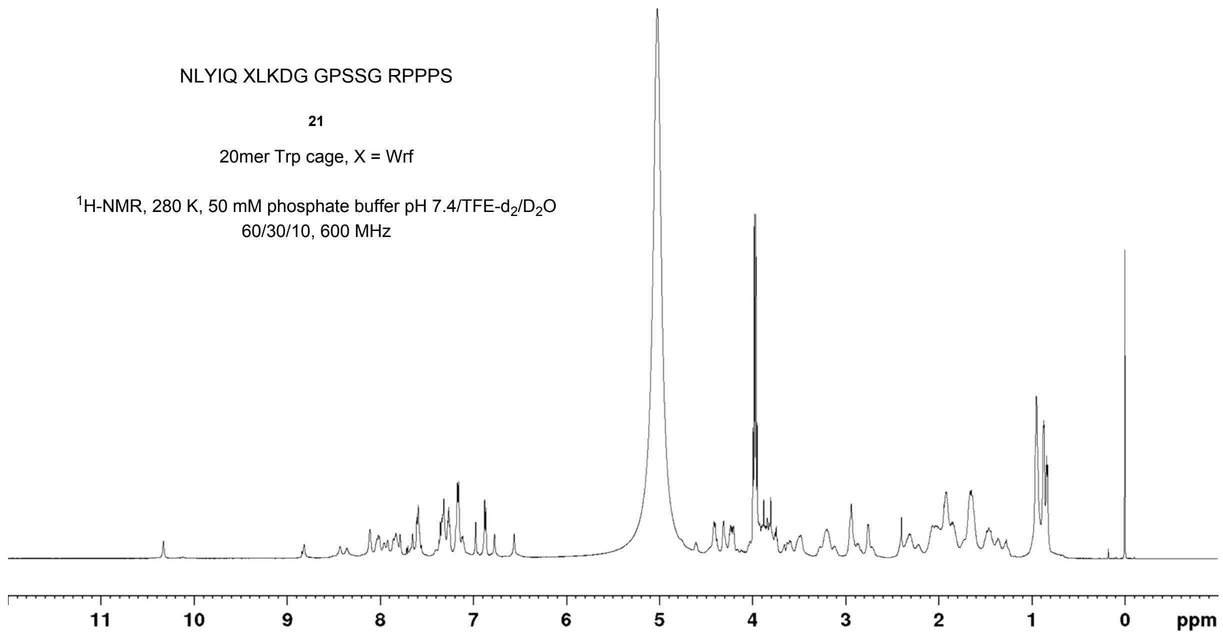

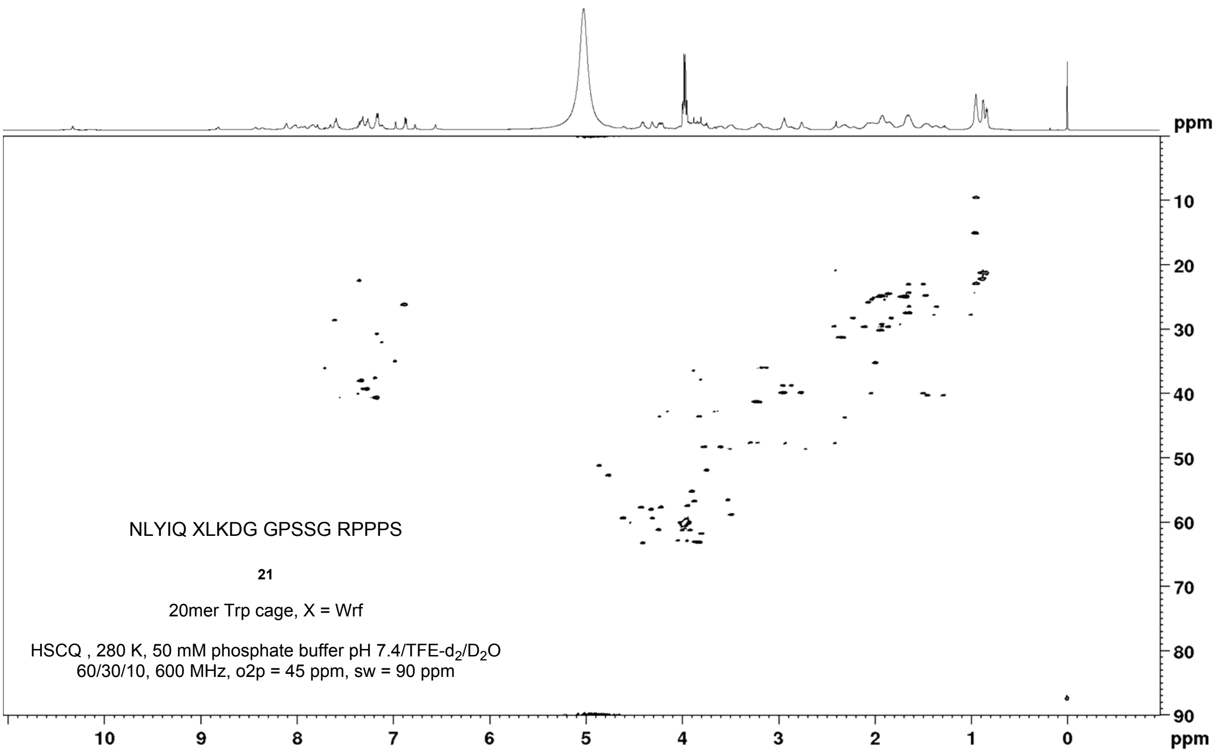
ppm
10
20
30
40
50
60
70
80
90
NLYIQ XLKDG GPSSG RPPPS
21
20mer Trp cage, X = Wrf
HSCQ , 280 K, 50 mM phosphate buffer pH 7.4/TFE-d2/D2O
60/30/10, 600 MHz, o2p = 45 ppm, sw = 90 ppm
10 9 8 7 6 5 4 3 2 1 0
ppm

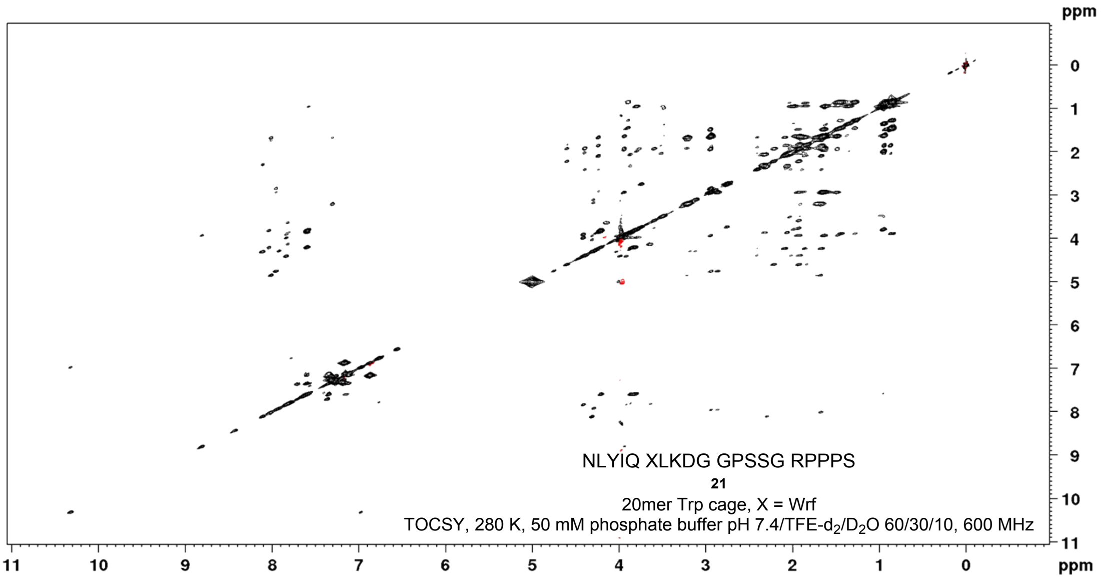
ppm
0 1 2 3 4 5 6 7 8 9 10 11
NLYIQ XLKDG GPSSG RPPPS
21
20mer Trp cage, X = Wrf
TOCSY, 280 K, 50 mM phosphate buffer pH 7.4/TFE-d2/D2O 60/30/10, 600 MHz
11 10 9 8 7 6 5 4 3 2 1 0
ppm

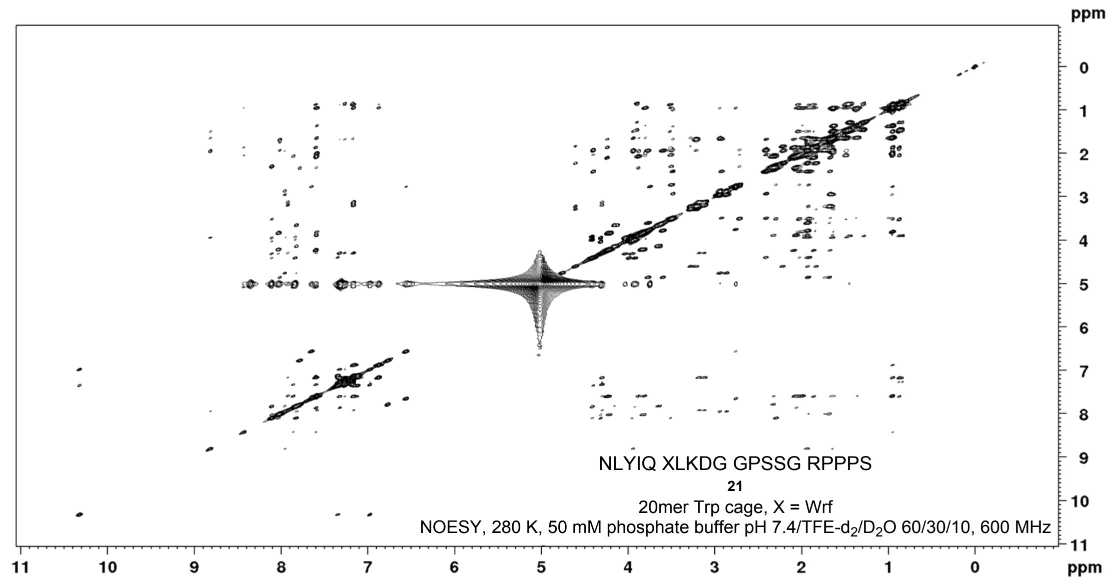

NLYIQ XLKDG GPSSG RPPPS
21
20mer Trp cage, X = Wrf
NOESY, 280 K, 50 mM phosphate buffer pH 7.4/TFE-d2/D2O 60/30/10, 600 MHz
ppm

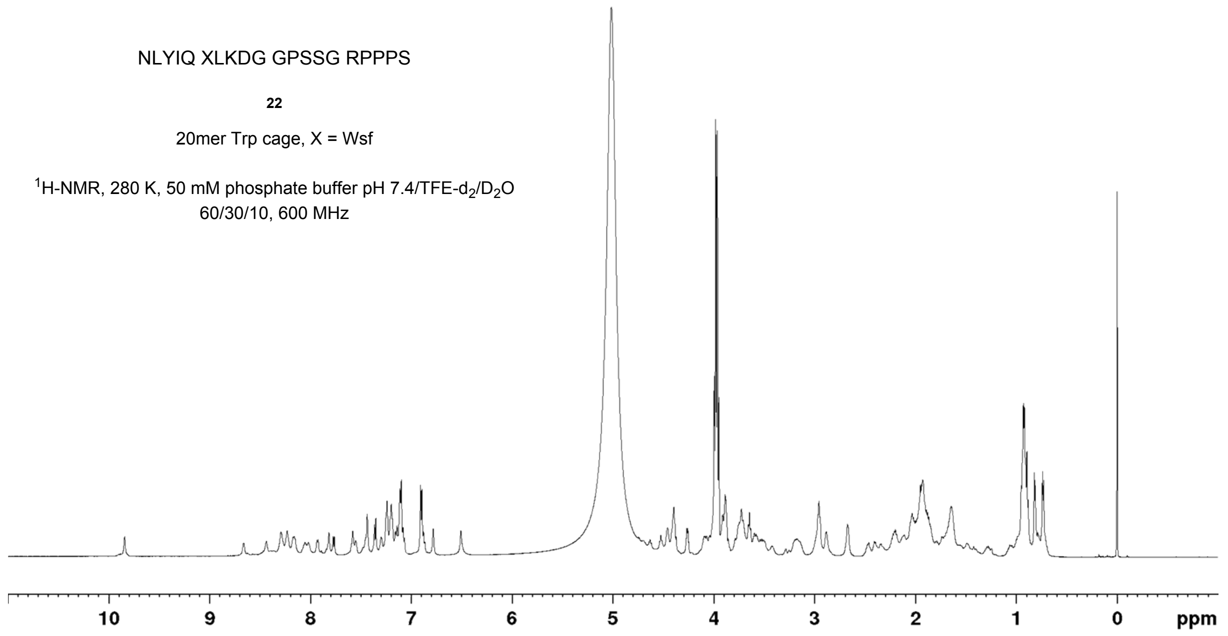

NLYIQ XLKDG GPSSG RPPPS
22
20mer Trp cage, X = Wsf
1H-NMR, 280 K, 50 mM phosphate buffer pH 7.4/TFE-d2/D2O 60/30/10, 600 MHz
ppm

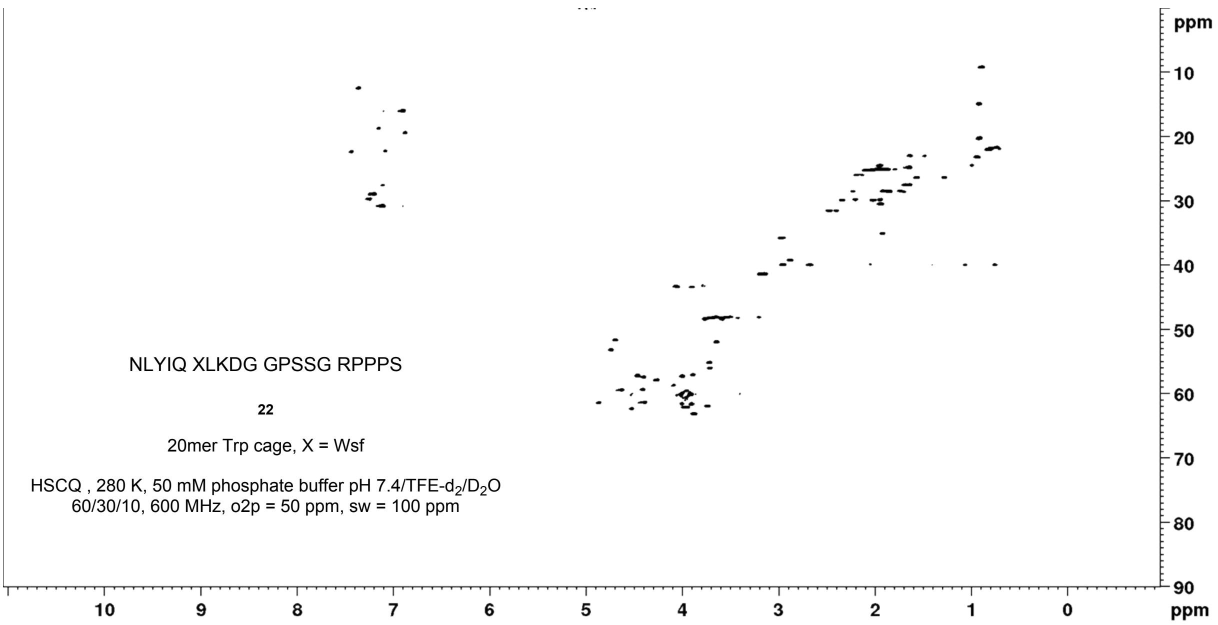
ppm
10
20
30
40
50
60
70
80
90
10
9
8
7
6
5
4
3
2
1
0
ppm
NLYIQ XLKDG GPSSG RPPPS
22
20mer Trp cage, X = Wsf
HSCQ , 280 K, 50 mM phosphate buffer pH 7.4/TFE-d2/D2O
60/30/10, 600 MHz, o2p = 50 ppm, sw = 100 ppm

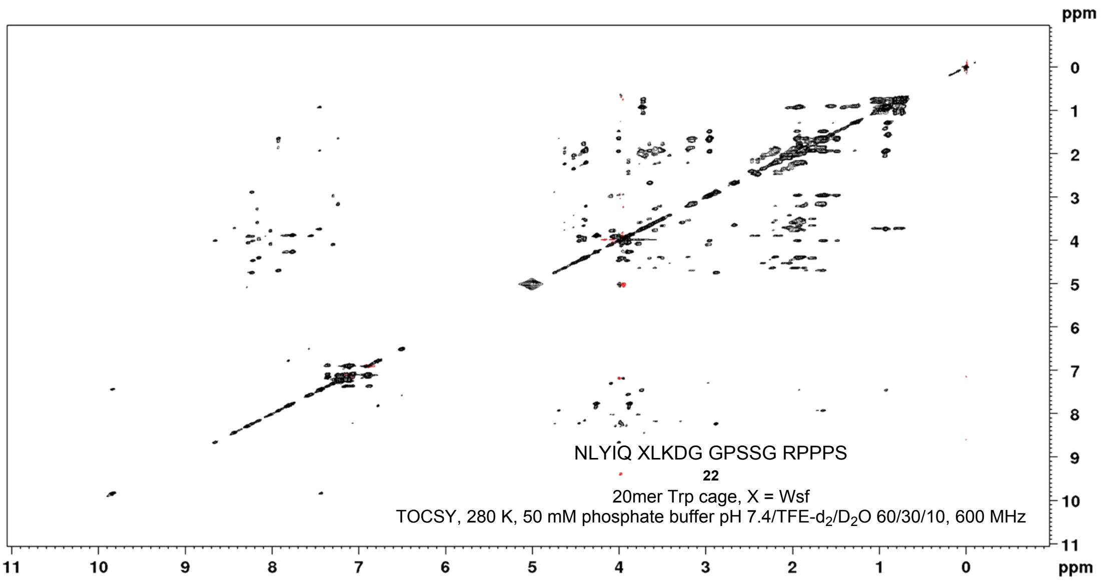
ppm
0
1
2
3
4
5
6
7
8
9
10
11
NLYIQ XLKDG GPSSG RPPPS
22
20mer Trp cage, X = Wsf
TOCSY, 280 K, 50 mM phosphate buffer pH 7.4/TFE-d2/D2O 60/30/10, 600 MHz
11
10
9
8
7
6
5
4
3
2
1
0
ppm

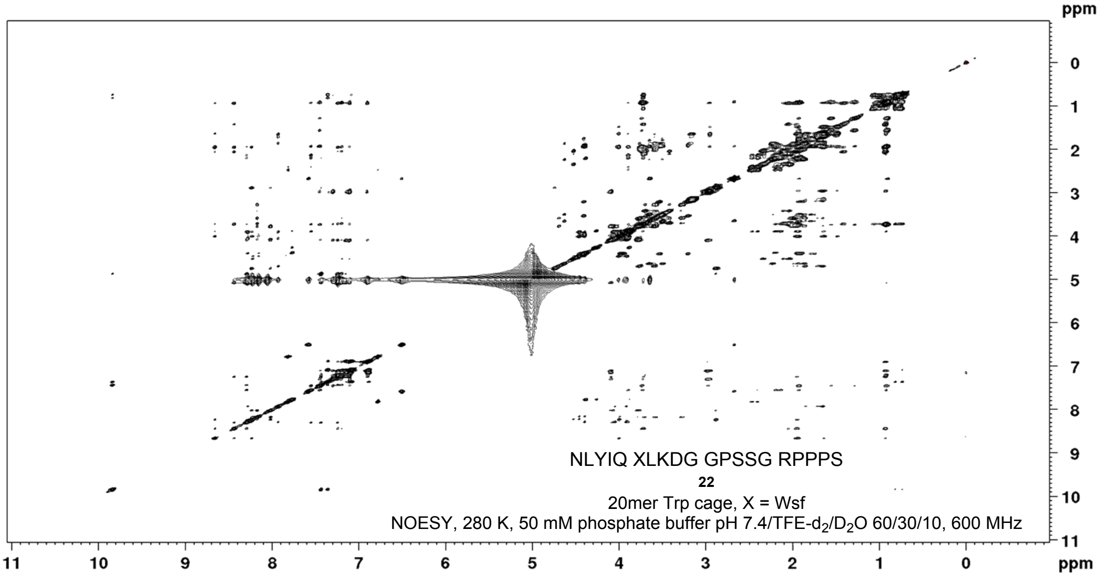

NLYIQ XLKDG GPSSG RPPPS

22

20mer Trp cage, X = Wsf

NOESY, 280 K, 50 mM phosphate buffer pH 7.4/TFE-d_2/D_2O 60/30/10, 600 MHz
